# Difunctional oxidatively cleavable alkenyl boronates: application to cellular peroxide sensing from a fluorophore–quencher pair†

**DOI:** 10.1039/d5cc00090d

**Published:** 2025-01-27

**Authors:** Brittany M. Klootwyk, Grace M. Fleury, Savannah Albright, Alexander Deiters, Paul E. Floreancig

**Affiliations:** a Department of Chemistry, University of Pittsburgh Pittsburgh Pennsylvania 15260 USA florean@pitt.edu

## Abstract

This manuscript describes the development of difunctional alkenyl boronates that contain an oxidatively releasable cargo and an amine for attaching to groups that can improve physical properties or enhance cellular targeting. The design is applied to a FRET-based system that delivers a selective fluorescence response in oxidatively stressed cells.

## Abstract 2

This manuscript details a difunctional oxidatively cleavable linker for applications in drug release. H_2_O_2_ promotes fluorophore liberation from a FRET pair, which leads to a selective fluorescent response in oxidatively stressed cancer cells.

## Graphical abstract

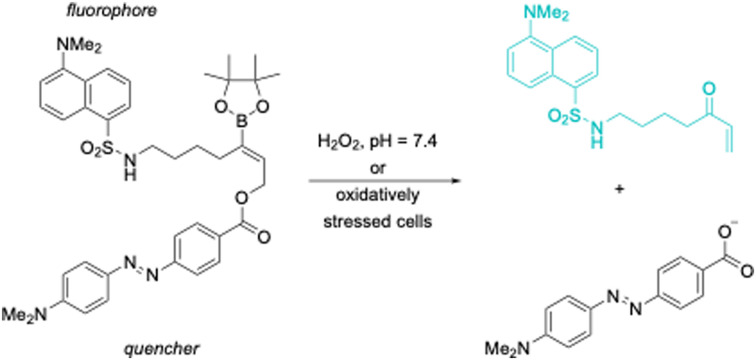

Numerous medical conditions, including cancer, arthritis, neurodegeneration, viral infection, diabetes, and reperfusion injury, lead to oxidatively stressed cells.^1^ Oxidative stress results in heightened concentrations of hydrogen peroxide, which can be exploited for the release of probes or drugs from boron-containing precursors.^2^ Borylated benzylic carbamates and carbonates, which undergo a 1,6-elimination upon reacting with H_2_O_2_, have been utilized extensively for this purpose, as illustrated (Scheme 1) by the oxidation of 1 to release the positron emitting tomography probe 2 along with quinone methide 3.^3^ We have developed alternatives to the borylated benzylic group to promote oxidative release, with the borylated allyloxy (BAO) group releasing compounds through a 1,4-elimination^4^ and the α-boryl ether promoting oxidative 1,2-elimination.^5^ These species show heightened reactivity in comparison to benzylic variants, thereby allowing for their attachment through stable ether and acetal linkages. The peroxide-mediated cleavage of 4 to release the pederin analogue 5 and acrolein (6) provides a powerful example of the utility of the design.^4*c*^ Compound 4 is a potent cytotoxin toward cancer cell lines while being far less toxic toward non-cancerous cells and acrolein, when released through a slow, controlled oxidative cleavage, proved to be non-toxic.

**Scheme 1:**
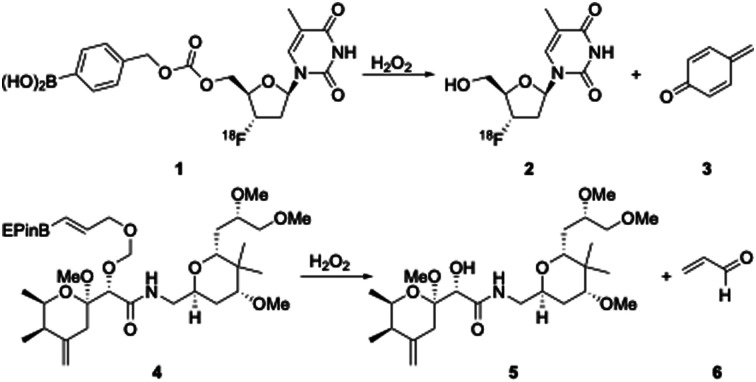
Probe and drug release through boronate oxidation. EPin = tetraethyl pinacol.

The selectivity that results from using boron-containing prodrugs might be enhanced by incorporating an additional functional group that could be used for attachment to antibodies,^6^ nanoparticles,^7^ or cell delivery agents.^8^

Difunctionalized arylboronates have been reported by Shabat^9^ for oxidative signal amplification through dendrimer breakdown, and by Spring,^10^ who reported an oxidatively labile antibody-directed conjugate. We became intrigued by the possibility of increasing the functional group content on the BAO subunit to enhance its utility while retaining its high oxidative cleavage rate. BAO groups can be functionalized (Scheme 2) on the alkene, where 7 will release an alcohol and form 8, or at the allylic position, where 9 will release the alcohol and 10.

**Scheme 2:**
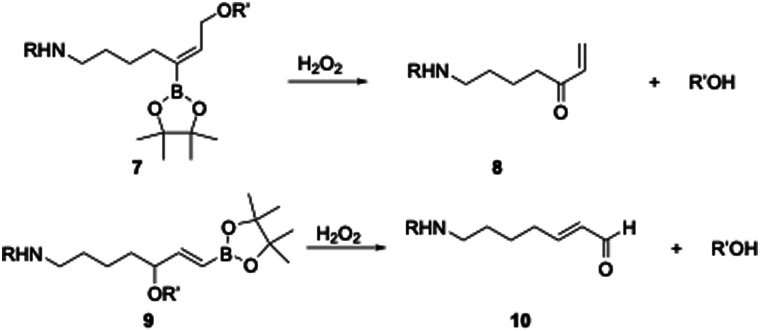
Oxidative carbon–oxygen bond cleavage from functionalized BAO groups. R can be a cell-targeting group, R′OH can be a drug or biological probe.

This manuscript describes the synthesis of difunctionalized BAO groups inspired by 7 and 9, and their functionalization with a FRET pair. The peroxide-mediated cleavage of these molecules leads to a time-dependent fluorescence response. Cellular studies show that these probes can distinguish oxidatively stressed cancer cell lines from non-cancerous cells. Cleavage in non-cancerous cells can be initiated through the addition of exogenous H_2_O_2_.

The synthesis of the vinyl-substituted linker is shown in Scheme 3. Hydroxymethylation of 6-chloro-1-hexyne (11) followed by chloride displacement by phthalimide provides propargylic alcohol 12. Removal of the phthalate group and sulphonylation with dansyl chloride delivered the fluorescent sulphonamide 13. Hydroboration under Carretero's conditions^11^ produced vinyl boronate 14. Acylation of 14 proved to be unexpectedly challenging due to the electron-withdrawing effect of the boronate group, though Katritzky's acyl benzotriazole reagent 15^12^ proved to be an excellent agent to complete the synthesis of the fluorophore–quencher paired ester 16.

**Scheme 3:**
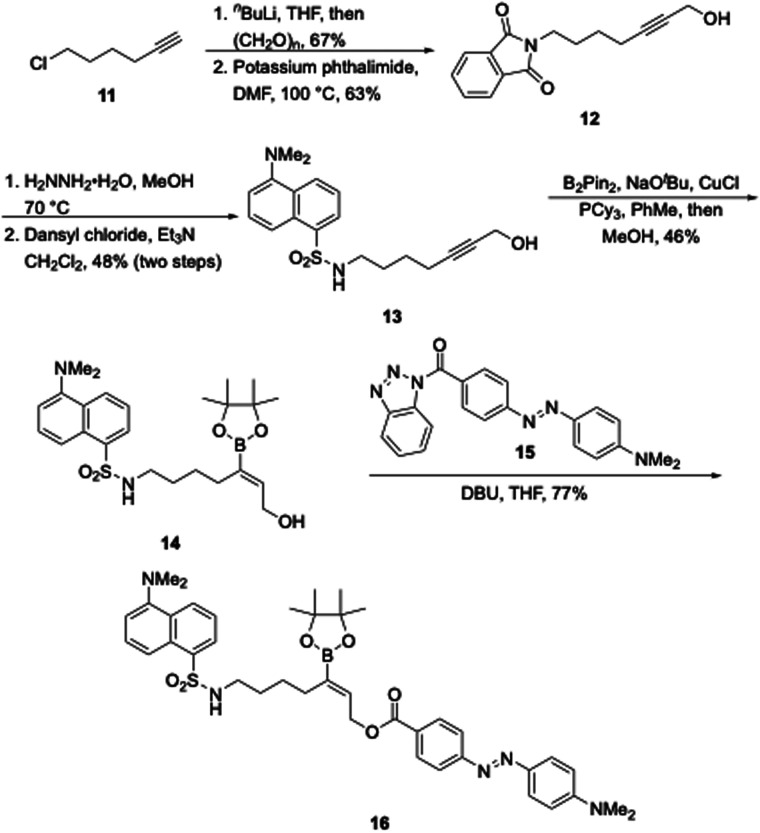
Synthesis of a vinyl-substituted oxidatively cleavable latent fluorophore.

Substitution at the allylic position offers additional possibilities for linker synthesis, including cross-metathesis^4*c*,13^ or alkyne hydroboration, in cases where a substrate is not compatible with copper-mediated borylation. The synthesis of the allylic-substituted linker is shown in Scheme 4. Sulphonylation of 6-amino-1-hexanol (17) with dansyl chloride provided the fluorescent sulphonamide 18. A Swern oxidation followed by the addition of lithium trimethylsilyl acetylide followed by silyl cleavage produced propargyl alcohol 19. Acylation with 15 and a subsequent hydroboration with pinacol borane in the presence of Cp_2_Zr(H)Cl^14^ completed the synthesis of 20.

**Scheme 4:**
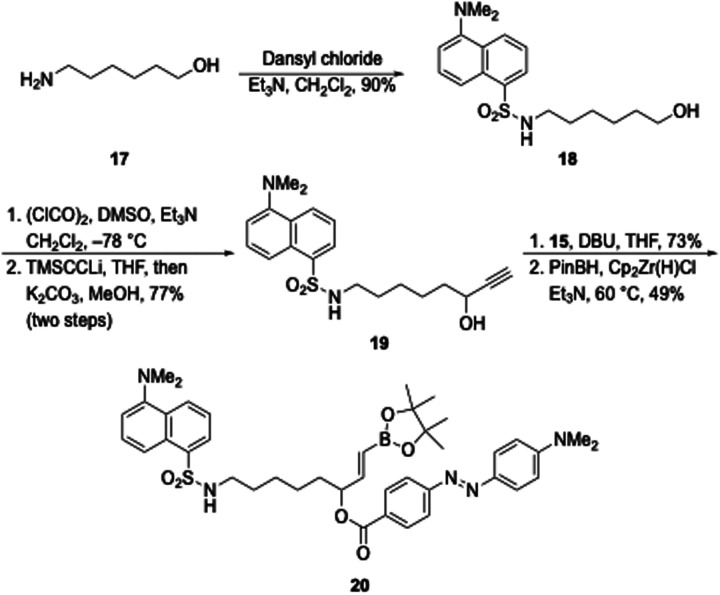
Synthesis of an allylic-substituted latent fluorophore.

The oxidative release of compounds 16 and 20 was initially monitored by ^1^H NMR spectroscopy (Scheme 5). Compound 16 was dissolved in DMSO-d6 and pH = 7.4 buffer and compound **20** was dissolved in CD_3_CN and pH = 7.4 buffer (1 mM final concentration for both) and treated with H_2_O_2_·urea (30 equiv.) at 37 °C. Compound 16 reacted to completion within 10 min to form 21 in 89% yield, as determined by comparison to an internal standard, along with acid 22. Similarly, 20 reacted to completion in 11 min to form 23 in 90% yield accompanied by 22. The negative control compound 24 was unreactive toward the reaction conditions, thereby confirming that the cleavage was dependent upon boronate oxidation.

**Scheme 5:**
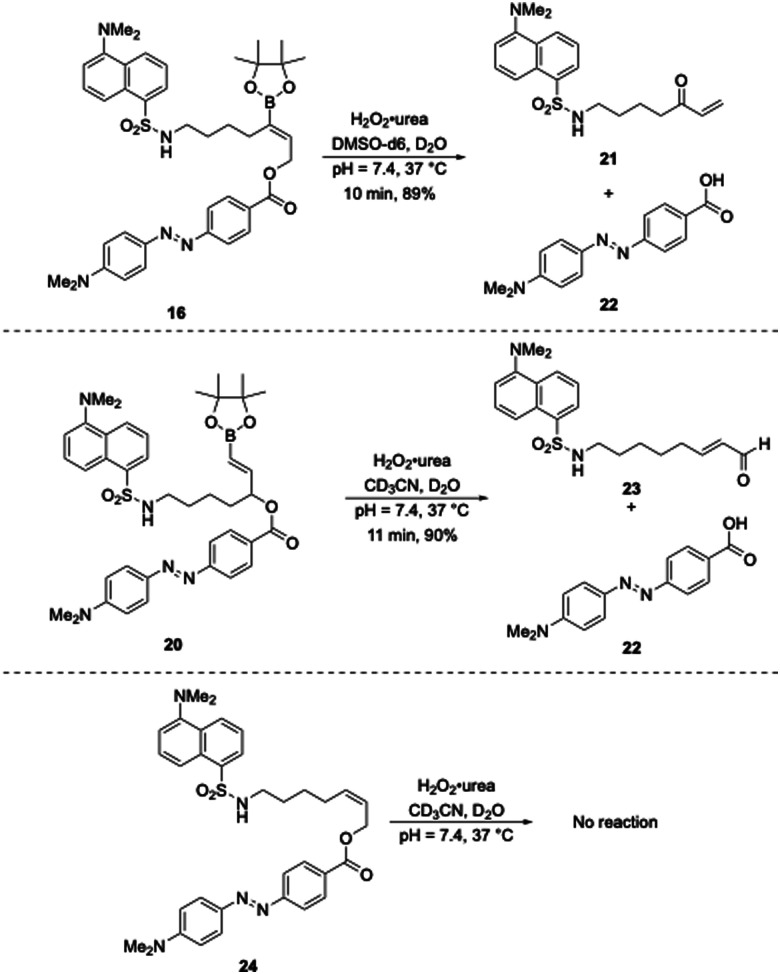
Oxidative release of the latent fluorophores monitored by ^1^H NMR.

The results of the NMR study led us to pursue a fluorescence-based assay to confirm that these processes could be viable at lower substrate and peroxide concentrations. Exposing 16 (final concentration = 200 μM) to H_2_O_2_·urea (30 equiv.) in DMSO and pH = 7.4 buffer at 37 °C showed a time-dependent increase in fluorescence until reaching a maximum within 3 h (Fig. 1A). A few aspects of this study were notable. Compound 21 showed an upward drift over time, which could arise from enone oxidation (see below). The fluorescence intensity did not meet the values that were seen from a 200 μM solution of 21, and the curve changes its shape at approximately 45% conversion. However when we tested the fluorescence intensity of a 1 : 1 mixture of 21 and 22 (200 μM of each) we observed good agreement with the maximum that was achieved in the release experiment. This indicates that bimolecular quenching accounts for the discrepancies from the initially expected results. Further evidence for intermolecular quenching arose from a variation on Blackmond's similar [“excess”] experiment^15^ in which the experiment commenced with 111 μM 16 and 91 μM 21 (the approximate concentrations of each at the point of the reaction in which the curve shape changed). This experiment showed a higher final fluorescence intensity, supporting our hypothesis of intermolecular quenching by 21. No release was observed from 24 in the presence of H_2_O_2_ or from 16 in the absence of H_2_O_2_.

**Fig. 1:**
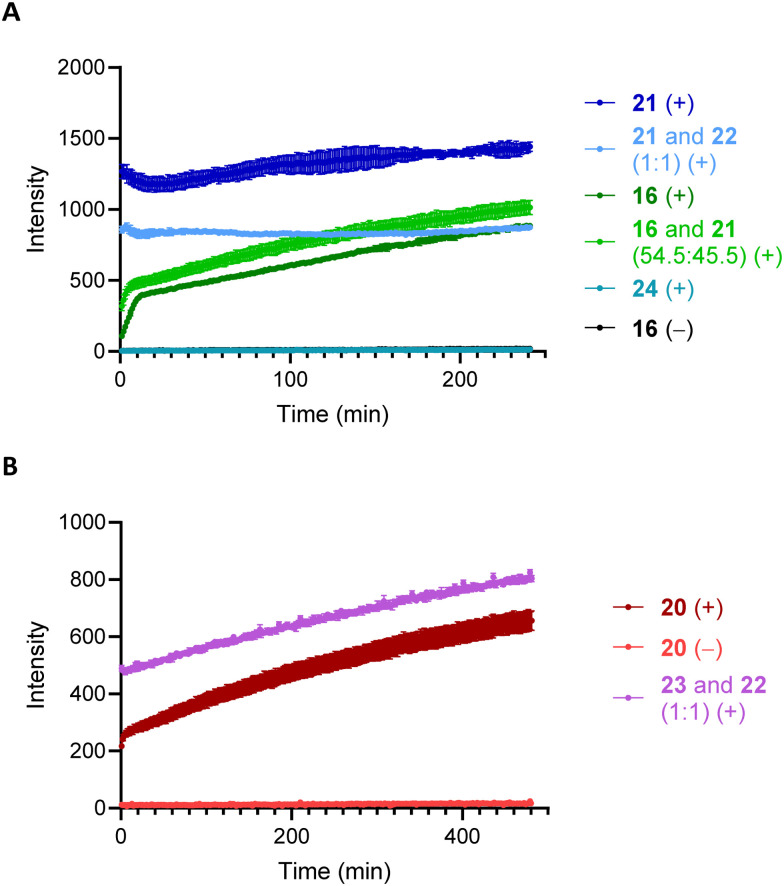
Fluorescence intensity of 16 (A) and 20 (B) in the presence (+) and absence (−) of H_2_O_2_ (6 mM) in DMSO and pH 7.4 buffer (200 μM initial substrate concentration) at 37 °C. The excitation wavelength was 335 nm and the emission wavelength was 534 nm.

The cleavage of 20 also proceeded efficiently, showing a similar pattern to the cleavage of 16. Identifying a precise time for complete release was not possible because the independently synthesized α,β-unsaturated aldehyde 23 showed an increase in fluorescence under the cleavage conditions. We postulate that the enal partially quenches fluorescence from the dansyl, in analogy to studies showing fluorescence quenching by unsaturated carbonyl groups.^16^ Prolonged exposure to H_2_O_2_ can result in nucleophilic epoxidation and a reduction in quenching. Epoxidation can occur under the cleavage conditions and that the epoxide shows no drift in a fluorescence study under the reaction conditions.^17^

Our previous study of oxidatively cleavable pederin-based prodrugs guided our selection of cell lines for monitoring *in vitro* fluorophore release. The previous study showed that the prodrug required exogenous H_2_O_2_ in non-cancerous HEK293T cells to promote a cytotoxic response, promoted a moderate cytotoxic response in U87 glioblastoma cells that was enhanced by exogenous H_2_O_2_, and was potently cytotoxic in B16 melanoma cells in the absence or presence of exogenous H_2_O_2_. We hypothesized that this trend correlated with the levels of endogenous of H_2_O_2_ in the cells, with stronger cytotoxic responses correlating to higher peroxide concentrations.

The results of the cellular studies are shown in Fig. 2. We selected compound 16 for these studies with compound 24 as the negative control. The cells were exposed to solutions of 16 or 24 in media (20 μM) for 45 min, then the media was exchanged with PBS buffer containing either H_2_O or H_2_O_2_ (100 μM). The responses of the cells were observed after an additional 45 min incubation through a DAPI filter that allows for absorption at 395 nm and emission at 460 nm. These wavelengths are not ideal for observing the release of 21, which has an absorption *λ*_max_ of 330 nm and an emission *λ*_max_ of 500 nm. Despite the non-optimal wavelengths for absorption and emission and the relatively short incubation times, we observed notable fluorescence in U87 and B16 cells in the absence of exogenous H_2_O_2_ (Fig. 2A, images were pseudocolored to cyan). As expected, the response appeared stronger upon the addition of H_2_O_2_, though the large error in the peroxide treated B16 cells rendered the difference statistically non-significant (Fig. 2B). Fluorescence was not visible in HEK cells in the absence of added H_2_O_2_ but appeared when H_2_O_2_ was added, indicating that the lack of an inherent fluorescent response did not result from 16 being impermeable in these cells.

**Fig. 2:**
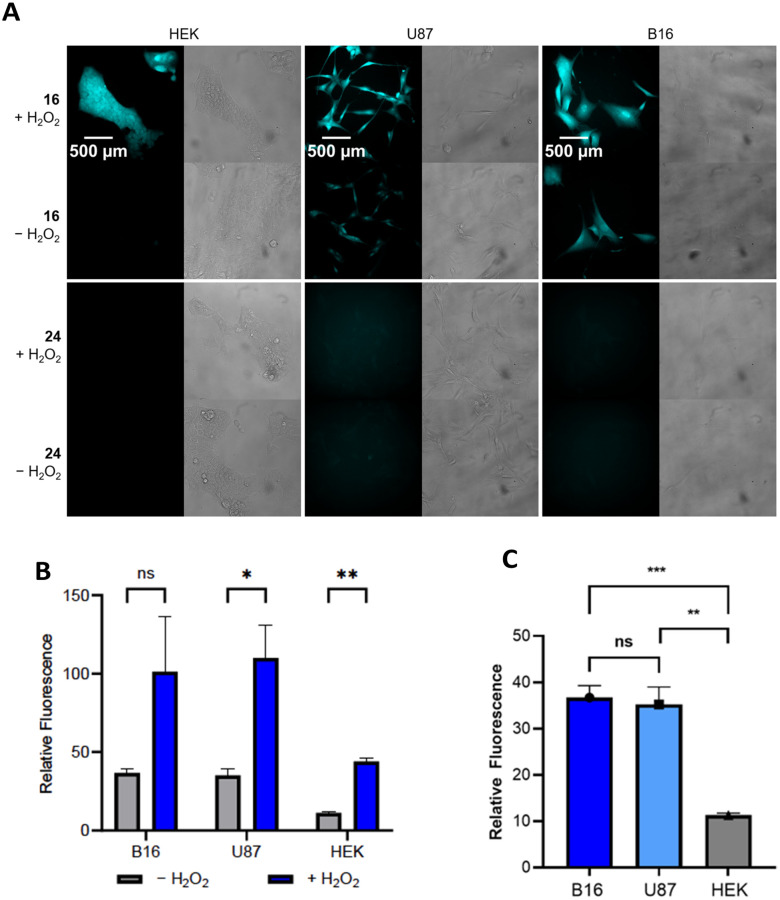
Fluorescence response of cells to 16 and 24. Cells were incubated with the compound in media (20 μM) for 45 min at 37 °C, then the media was removed and replaced either with buffer (−) or a solution of H_2_O_2_ (+). (A) Images of cells after being exposed to 16 or 24 for 45 min. Fluorescence was imaged on a Zeiss Axio Observer Z1 using a 40x objective and DAPI (Chroma filter 49028) filter (*e*_x_ = 395 nm, *e*_m_ = 460 nm). Gray images are brightfield. (B) Relative responses of the cells after being exposed to 16 and 24 after 45 min in the absence and presence of exogenous H_2_O_2_. See the ESI† for details on the calculations of relative fluorescence. (C) Comparison of the relative fluorescence for the cell lines in the absence of exogenous H_2_O_2_. * = *P* < 0.05, ** = *P* < 0.001, *** = *P* < 0.005, ns = not statistically different.

The negative control 24 produced no fluorescence in any cell line, which is consistent with the cleavages being initiated by peroxide rather than a non-specific process. This allowed for a quantitative comparison of the responses of the different cell lines toward 16 against their responses to 24 (Fig. 2B). Although the fluorescence in the response of HEK cells in the absence of exogenous H_2_O_2_ was not visually apparent, a minor response was detected through imaging. This is expected since most cells produce at least low concentrations of H_2_O_2_.^18^ Oxidative stress also leads cells to produce low levels of hypochlorite and peroxynitrite,^19^ and these agents are significantly more effective at boronate oxidation than H_2_O_2_.^20^ Therefore the responses could be augmented by the actions of these agents, though the use of DMSO as a co-solvent for the studies reduces hypochlorite.^21^ The responses of the U87 and B16 cells were approximately equivalent and notably higher than the response of the HEK cells (Fig. 2C).

We have shown the synthesis and peroxide-mediated cleavage of two difunctional vinyl boronate linkers that are suitable for drug delivery and release applications. Differential functionalization of the linkers is facile, as demonstrated by the attachment of the dansyl/dabcyl FRET pair. NMR studies showed a rapid and efficient cleavage of the vinyl boronates to release the dabcyl group. The cleavage was monitored at sub-millimolar concentrations through fluorescence spectroscopy and confirmed the expected time-dependent loss of quenching. Exposing the boronate-containing FRET pair to oxidatively stressed cancer cells resulted in a substantially greater fluorescent response in comparison to results with non-oxidatively stressed epithelial cells. These results support the capacity to apply the linkers to the synthesis of antibody–drug conjugates, nanoparticles, and cell-permeating agents that utilize oxidative stress for site-selective drug release.

This work was supported by funding from the National Institutes of Health (R01GM127153 to P. E. F. and A. D., R01GM140655 to P. E. F., and R01GM145086 to A. D.). G. M. F. was supported through a McKeever Award. S. A. was supported through an Andrew Mellon Predoctoral Fellowship.

## Data availability

The data supporting this article have been included as part of the ESI.†

## Conflicts of interest

There are no conflicts to declare.

## Supplementary Material

CC-061-D5CC00090D-s001
