# Supplementary material for: Difunctional oxidatively cleavable alkenyl boronates: application to cellular peroxide sensing from a fluorophore–quencher pair
Source: Chem Commun (Camb). 2025 Jan 27;61(16):3375–8. doi: 10.1039/d5cc00090d (PMC11784462; doi:10.1039/d5cc00090d)
Supplement: CC-061-D5CC00090D-s001 [file CC-061-D5CC00090D-s001.pdf]

Supplementary Information (SI) for ChemComm.
This journal is © The Royal Society of Chemistry 2025

Supporting Information

Difunctional oxidatively cleavable alkenyl boronates: application to cellular peroxide sensing from a fluorophore-quencher pair

Brittany M. Klootwyk, Grace M. Fleury, Savannah Albright, Alexander Deiters and Paul E. Floreancig*

Department of Chemistry

University of Pittsburgh

Pittsburgh, Pennsylvania 15260, United States

florean@pitt.edu

Table of Contents

General Experimental S3

Experimental Procedures and Characterization S4

^{1}H NMR Release Studies S13

Fluorescence Assays S21

Cell Culture and Fluorescence Micrography S22

^{1}H, ^{13}C, and ^{11}B NMR Spectra S23

References S46

General Experimental

Proton (^1H) NMRs were recorded on Bruker Avance spectrometers at 300, 400, 500, and 600 MHz. Carbon (^{13}C) NMRs were recorded on Bruker Avance spectrometers at 100, 125, 151, and 176 MHz. Note that carbons that are attached to boron are broadened through quadrupolar relaxation[1] to the extent that they cannot be seen except in highly concentrated samples. The chemical shifts are reported in parts per million (ppm) on the delta (δ) scale. The solvent peak was used as a reference value, for ^1H NMR: $CDCl_3$ = 7.26 ppm, CD_3CN = 1.94 ppm, for ^{13}C NMR: $CDCl_3$ = 77.2 ppm, CD_3CN = 1.3 ppm and 118.3 ppm. Boron (^{11}B) NMRs were recorded on Bruker Avance spectrometers at 128 or 160 MHz and the chemical shifts were referenced to the IUPAC-approved unified scale.[2] The coupling data are reported as follows: s = singlet; d = doublet; t = triplet; q = quartet; m = multiplet. High resolution mass spectra were collected on a ThermoFisher Q-Exactive Orbitrap instrument.

All distillations were performed under N_2 unless otherwise stated. Methylene chloride and acetonitrile were distilled from calcium hydride. Tetrahydrofuran was distilled over sodium/benzophenone. Methanol was distilled from ground calcium sulfate. Analytical TLC was performed on E. Merck pre-coated (25 mm) silica gel 60 F254 plates. Visualization was done under UV (254 nm) and by staining with p-anisaldehyde stain. Flash chromatography was done using SiliCycle SiliaFlash P60 40-63µm 60 Å silica gel. Reagent grade ethyl acetate, methyl tert-butyl ether, diethyl ether, acetonitrile, dichloromethane, and hexanes (commercial mixture) were purchased from Fisher Scientific and were used as-is for chromatography. All reactions were performed in flame-dried glassware under a positive pressure of Ar with magnetic stirring unless noted otherwise. All reactions that required heating were warmed with sand baths.

Boronate compounds should be rapidly purified by column chromatography to avoid decomposition. We found that using boric acid-treated silica gel helped to limit product degradation. Boric acid impregnated silica gel was generated following a previously reported literature procedure.[3] Silica gel (300 mL) was mixed with boric acid (28.0 g) and ethanol (550 mL) for two hours at room temperature. The silica gel was filtered and washed with ethanol three times (200 mL). The silica gel was dried overnight on a vacuum filtration setup and then dried in a 100 °C oven for 48 hours.

Experimental Procedures and Characterization Data

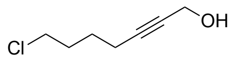

7-Chlorohept-2-yn-1-ol (S1)

A solution of 6-chloro-1-hexyne (7.287 g, 62.50 mmol) in anhydrous diethyl ether (63 mL) was cooled to –78 °C. Over 15 min, *n*BuLi (2.5 M in hexanes, 30 mL, 75 mmol) was added to the solution dropwise. The reaction was stirred for an additional 15 min at –78 °C. Paraformaldehyde (2.440 g, 81.25 mmol) was quickly added as a single portion and then the reaction was stirred vigorously at rt for 3 h. The reaction was quenched with saturated ammonium chloride (aqueous, 100 mL), extracted with ethyl acetate (3x50 mL), dried over magnesium sulfate, and filtered. After concentration of the crude liquid, the alkyne was purified via column chromatography (10-30% EtOAc in hexanes) to give **S1** as a liquid (6.14 g, 67%).

^{1}H NMR (400 MHz, $CDCl_3$) δ 4.24 (t, *J* = 2.1 Hz, 2H), 3.56 (t, *J* = 6.6 Hz, 2H), 2.27 (tt, *J* = 7.0, 2.1 Hz, 2H), 1.88 (m, 2H), 1.66 (overlapping m, 3H)
^{13}C NMR (100 MHz, $CDCl_3$) δ 85.7, 79.1, 51.5, 44.6, 31.6, 25.8, 18.2
HRMS (ESI) *m/z* calcd. for $C_7H_{12}OCl$ $[M+H]^+$ 147.0571, found 147.0571
IR (ATR, neat) 3328, 2937, 2870, 1433, 1301, 1132, 1008, 727, 648 cm^{-1}

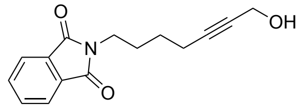

2-(7-Hydroxyhept-5-yn-1-yl)isoindoline-1,3-dione (12)

Potassium phthalimide (0.644 g, 3.479 mmol) was added to a solution of **S1** (0.400 g, 2.32 mmol) in DMF (6 mL). The reaction was heated to 100 °C and stirred overnight. After cooling to rt, a solution of diethyl ether and water (1:1 by volume, 20 mL) was added. The layers were separated, and the organic layer was washed with ether (10 mL) three times. The combined organic layers were washed twice with water (100 mL), dried over $MgSO_4$, filtered, and concentrated. Phthalimide **12** (0.376 g, 63%) was isolated as a white solid after column chromatography (40% EtOAc in hexanes).

^{1}H NMR (500 MHz, $CDCl_3$) δ 7.84 (dd, *J* = 5.3, 3.1 Hz, 2H), 7.71 (dd, *J* = 5.4, 3.0 Hz, 2H), 4.23 (m, 2H), 3.72 (t, *J* = 7.2 Hz, 2H), 2.28 (t, *J* = 7.0 Hz, 2H), 1.81 (quintet, *J* = 7.4 Hz, 2H), 1.56 (quintet, *J* = 7.3 Hz, 2H)
^{13}C NMR (126 MHz, $CDCl_3$) δ 168.6, 134.1, 132.2, 123.4, 85.7, 79.3, 51.5, 37.6, 27.6, 25.6, 18.4
HRMS (ESI) *m/z* calcd. for $C_{15}H_{16}O_3N$ $[M+H]^+$ 258.1125, found 258.1123
IR (ATR, neat) 3459, 2917, 2850, 1769, 1705, 1467, 1437, 1396, 1372, 1239, 1020, 908, 719, 530 cm^{-1}
Melting Point: 84 – 86 °C

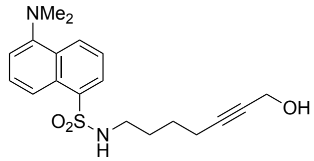

5-(Dimethylamino)-*N*-(7-hydroxyhept-5-yn-1-yl)naphthalene-1-sulfonamide (13)

$NH_2NH_2 \cdot H_2O$ (0.28 mL, 0.19 g, 3.0 mmol, 51% solution in H_2O) was added to a solution of **12** (0.585 g, 2.27 mmol) in dry CH_3OH (23 mL). The reaction was heated to reflux at 70 °C. The resulting solution was stirred for 2 h. The reaction was cooled to room temperature and concentrated *in vacuo*. Azeotropic drying with benzene afforded the crude amine which was carried forward without further purification.

The amine was dissolved in distilled CH_2Cl_2 (7.6 mL) and cooled to 0 °C. Triethylamine (0.48 mL, 0.35 g, 3.4 mmol) was added to the solution. After 10 min, dansyl chloride (0.920 g, 3.41 mmol) was added in a single portion and the reaction was warmed to rt and stirred overnight. The mixture was cooled to 0 °C and quenched with saturated aqueous $NaHCO_3$ (10 mL). The aqueous layer was extracted three times with CH_2Cl_2 (10 mL) and the combined organic layers dried over $MgSO_4$, filtered, and concentrated *in vacuo*. The resulting oil was purified via column chromatography (10% then 20% EtOAc in hexanes) to afford the desired product as a clear, viscous, vibrant green oil (0.39 g, 48% over two steps).

^{1}H NMR (500 MHz, $CDCl_3$) δ 8.54 (d, J = 8.5 Hz, 1H), 8.30 (d, J = 8.7 Hz, 1H), 8.24 (dd, J = 7.3, 0.9 Hz, 1H), 7.54 (m, 2H), 7.19 (d, J = 7.5 Hz, 1H), 4.85 (t, J = 6.1 Hz, 1H), 4.18 (app br s, 2H), 2.91 (overlapping m and s, 8H), 2.07 (tt, J = 6.8, 2.1 Hz, 2H), 1.90 (br s, 1H), 1.50 (quintet, J = 7.2 Hz, 2H), 1.38 (quintet, J = 7.2 Hz, 2H)
^{13}C NMR (126 MHz, $CDCl_3$) δ 152.1, 134.9, 130.6, 129.8, 128.5, 123.4, 118.9, 115.4, 85.6, 79.2, 77.4, 51.4, 45.6, 43.0, 28.8, 25.3, 18.2
HRMS (ESI) *m/z* calcd. for $C_{19}H_{25}O_3N_2S$ $[M+H]^+$ 361.1580, found 361.1582
IR (ATR, neat) 3494, 3292, 2941, 2857, 2834, 2788, 2249, 1573, 1454, 1406, 1310, 1230, 1201, 1141, 1074, 1009, 910, 790, 729, 625, 571, 538, 498 cm^{-1}

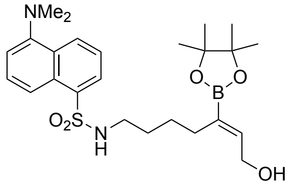

(*Z*)-5-(Dimethylamino)-*N*-(7-hydroxy-5-(4,4,5,5-tetramethyl-1,3,2-dioxaborolan-2-yl)hept-5-en-1-yl)naphthalene-1-sulfonamide (14)

A flask charged with copper (I) chloride (0.017 g, 0.17 mmol), tricyclohexylphosphine (0.047 g, 0.17 mmol), sodium *t*-butoxide (0.088 g, 0.92 mmol), and bis(pinacolato)diboron (0.254 g, 1.00 mmol) was purged with Ar (g). Toluene (1.2 mL) was added and the heterogenous mixture was cooled to 0 °C. A solution of alcohol **13** (0.300 g, 0.832 mmol) in toluene (1.1 mL) was added slowly then the reaction was warmed to rt. The reaction was stirred for 1 h, cooled to 0 °C, then quenched with methanol (0.17 mL, 0.13 g, 4.2 mmol) and stirred for five min. The contents of the flask were loaded directly onto a short silica plug (40% hexanes in EtOAc) and flushed to remove insoluble material. The crude material was concentrated under reduced pressure then purified via column chromatography (10% MTBE in CH_2Cl_2) to yield **14** (0.186 g, 46%) as a vibrant green oil. The regioisomeric vinyl boronate **S2** (0.049 g) was also isolated as a vibrant green oil as a mixture with other compounds.

14
^{1}H NMR (500 MHz, $CDCl_3$) δ 8.55 (d, J = 8.3 Hz, 1H), 8.31 (d, J = 8.6 Hz, 1H), 8.24 (dd, J = 7.3, 1.1 Hz, 1H), 7.53 (m, 2H), 7.19 (d, J = 7.5 Hz, 1H), 6.36 (t, J = 6.1 Hz, 1H), 4.80 (t, J = 6.4 Hz, 1H), 4.13 (d, J = 6.1 Hz, 2H), 2.91 (overlapping m and s, 8H), 1.98 (t, J = 7.5 Hz, 2H), 1.35 (quintet, J = 7.0 Hz, 2H), 1.25 (overlapping m and s, 14H)
^{11}B NMR (160 MHz, $CDCl_3$) δ 30.5
^{13}C NMR (126 MHz, $CDCl_3$) δ 143.9, 135.1, 130.4, 130.0, 129.83, 129.76, 128.4, 123.4, 119.0, 115.4, 83.7, 59.5, 45.6, 43.0, 29.8, 29.0, 27.9, 26.6, 24.9
HRMS (ESI) *m/z* calcd. for $C_{25}H_{38}O_5N_2BS$ $[M+H]^+$ 489.2589, found 489.2589
IR (ATR, neat) 3496, 3294, 2977, 2938, 2866, 1632, 1574, 1456, 1406, 1371, 1307, 1230, 1203, 1141, 1074, 910, 856, 790, 731, 685, 624, 571, 538, 498 cm^{-1}

S2

^{1}H NMR ($CDCl_3$, 500 MHz) δ 8.54 (d, *J* = 8.5 Hz, 1H), 8.26 (m, 2H), 7.55 (m, 2H), 7.19 (d, *J* = 7.4 Hz, 1H), 6.24 (t, *J* = 7.3 Hz, 1H), 4.62 (br t, 1H), 4.16 (d, *J* = 4.0 Hz, 2H), 2.88 (overlapping m and s, 8H), 2.01 (m, 2H), 1.26 (s, 12H)

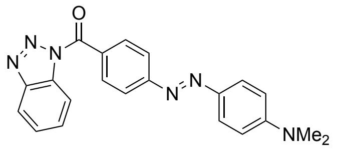

(*E*)-(1*H*-Benzo[*d*][1,2,3]triazol-1-yl)(4-((4-(dimethylamino)phenyl)diazenyl)phenyl)methanone (15)

Triethylamine (0.15 mL, 0.11 g, 1.04 mmol) was added to a solution of dabcyl acid (0.200 g, 0.743 mmol) and 1-(methanesulfonyl)-1H-benzotriazole (0.146 g, 0.743 mmol) in THF (3.72 mL). The reaction was heated to 70 °C and stirred for 5 h. The reaction was cooled to rt overnight, without stirring, to allow for precipitation of the desired product. The precipitate was filtered by vacuum filtration and to afford the desired product as a dark red-purple powder (0.082 g, 30%) that was used without further purification. Characterization data matches that of published literature values.[4]

^{1}H NMR (300 MHz, $CDCl_3$) δ 8.41 (d, *J* = 8.3 Hz, 1H), 8.37 (d, *J* = 8.7 Hz, 2H), 8.18 (d, *J* = 8.1 Hz, 1H), 7.97 (dd, *J* = 16.6, 8.9 Hz, 4H), 7.72 (t, *J* = 7.7 Hz, 1H), 7.56 (t, *J* = 7.7 Hz, 1H), 6.77 (d, *J* = 9.2 Hz, 2H), 3.12 (s, 6H)

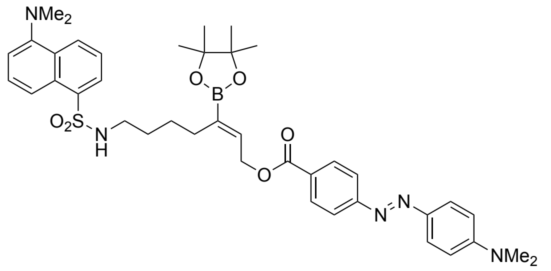

(*Z*)-7-((5-(Dimethylamino)naphthalene)-1-sulfonamido)-3-(4,4,5,5-tetramethyl-1,3,2-dioxaborolan-2-yl)hept-2-en-1-yl 4-((*E*)-(4-(dimethylamino)phenyl)diazenyl)benzoate (16).

A solution of boryl allyl alcohol **14** (0.035 g, 0.072 mmol) in THF (1.8 mL) was added to a flask charged with dabcyl derivative **15** (0.028 g, 0.076 mmol) under argon at rt. DBU (11 µL, 0.011 g, 0.076 mmol) was added and the reaction stirred for 24 h. The reaction was concentrated under reduced pressure. The crude material was purified via flash chromatography (30% EtOAc in hexanes with boric acid-treated silica gel) to yield the red-orange oil (0.041 g, 77%).

^{1}H NMR (600 MHz, $CDCl_3$) δ 8.54 (d, *J* = 8.4 Hz, 1H), 8.32 (d, *J* = 8.6 Hz, 1H), 8.25 (dd, *J* = 7.3, 1.1 Hz, 1H), 8.14 (d, *J* = 8.7 Hz, 2H), 7.91 (d, *J* = 9.2 Hz, 2H), 7.86 (d, *J* = 8.7 Hz, 2H), 7.53 (m, 2H), 7.17 (d, *J* = 7.5 Hz, 1H), 6.76 (d, *J* = 9.3 Hz, 2H), 6.42 (t, *J* = 6.1 Hz, 1H), 4.91 (t, 6.6 Hz, 1H), 4.86 (d, *J* = 6.2 Hz, 2H), 3.11 (s, 6H), 2.94 (q, *J* = 6.8 Hz, 2H), 2.88 (s, 6H), 2.09 (t, *J* = 7.6 Hz, 2H), 1.41 (quintet, *J* = 7.0 Hz, 2H), 1.33 (m, 2H), 1.26 (s, 12H)
^{11}B NMR (193 MHz, $CDCl_3$) δ 29.9
^{13}C NMR (151 MHz, $CDCl_3$) δ 166.3,156.2, 153.0, 143.9, 138.6, 135.1, 130.8, 130.7, 130.4, 130.2, 130.0, 129.8, 129.7, 128.4, 125.7, 123.4, 122.1, 119.0 (broad, B – C), 115.3, 111.6, 83.8, 61.7, 45.6, 42.9, 40.4, 29.0, 28.1, 26.4, 24.9
HRMS (ESI) *m/z* calcd. for $C_{40}H_{51}O_6N_5BS$ $[M+H]^+$ 740.3648, found 740.3651
IR (ATR, neat) 3297, 2976, 2930, 2864, 1713, 1598, 1519, 1444, 1421, 1363, 1312, 1266, 1134, 1093, 1009, 945, 860, 824, 791, 775, 736, 697, 625, 571, 540, 493 cm^{-1}
Melting Point: 63 – 66 °C

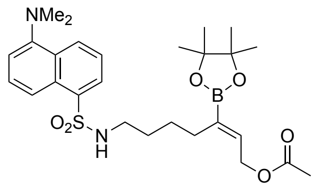

(*Z*)-7-((5-(Dimethylamino)naphthalene)-1-sulfonamido)-3-(4,4,5,5-tetramethyl-1,3,2-dioxaborolan-2-yl)hept-2-en-1-yl acetate (S3)

Acetic anhydride (3 µL, 0.003 g, 0.03 mmol) was added to a solution of boryl alcohol **14** (0.008 g, 0.016 mmol) in CH_2Cl_2 (0.115 mL). DMAP (35 µL, 0.18 mg, 0.0015 mmol, 43 mM in CH_2Cl) was added and then the reaction was stirred at rt for 30 min. The reaction mixture was loaded directly onto a normal phase SiO_2 column for purification (40% hexanes in Et_2O) to yield the desired product as a vibrant green oil (0.0065 g, 83 %).

^{1}H NMR (500 MHz, $CDCl_3$) δ 8.54 (d, J = 8.6 Hz, 1H), 8.30 (d, J = 8.7 Hz, 1H), 8.24 (dd, J = 7.3, 1.0 Hz, 1H), 7.53 (m, 2H), 7.18 (d, J = 7.5 Hz, 1H), 6.28 (t, J = 6.3 Hz, 1H), 4.83 (t, J = 6.2 Hz, 1H), 4.60 (d, J = 6.0 Hz, 2H), 2.90 (overlapping m and s, 8H), 2.04 (s, 3H), 2.01 (t, J = 7.6 Hz, 2H), 1.38 (quintet, J = 7.1 Hz, 2H), 1.27 (overlapping quintet and s, 14H)
^{11}B NMR (160 MHz, $CDCl_3$) δ 30.5
^{13}C NMR (126 MHz, $CDCl_3$) δ 171.0, 138.5, 135.1, 130.5, 130.1 129.8, 129.7, 128.5, 128.4, 123.4, 119.0 (broad, B – C), 115.3, 83.8, 61.2, 45.6, 43.0, 29.1, 28.0, 26.5, 24.9, 21.1
HRMS (ESI) *m/z* calcd. for $C_{27}H_{40}O_6N_2BS$ $[M+H]^+$ 531.2695, found 531.2701
IR (ATR, neat) 3293, 2977, 2938, 2866, 2788, 1738, 1636, 1574, 1456, 1407, 1374, 1358, 1311, 1230, 1142, 1091, 1073, 1043, 947, 912, 856, 790, 734, 684, 625, 571, 538, 498 cm^{-1}

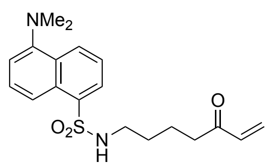

5-(Dimethylamino)-*N*-(5-oxohept-6-en-1-yl)naphthalene-1-sulfonamide (21)

A solution of acetate **S3** (0.018 g, 0.034 mmol) in CH_3CN (0.9 mL) was heated to 37 °C. PBS buffer (pH = 7.0, 0.9 mL) was added. After 5 minutes, hydrogen peroxide urea (0.032 g, 0.34 mmol) was added. The reaction was stirred for 2 h then cooled to room temperature and quenched with dimethyl sulfide (26 µL, 0.022 g, 0.34 mmol). The solution was concentrated *in vacuo* then redissolved in CH_2Cl_2 and extracted three times (3 mL). The organic layers were dried, filtered, then concentrated. The crude material was purified through column chromatography (40% EtOAc in hexanes) to yield the desired enone as a vibrant green oil (0.008 g, 67%). *Note*: The enone is prone to *decomposition when neat* and was kept under an inert atmosphere, in solution, and the vial wrapped in aluminum foil during storage.

^{1}H NMR (500 MHz, $CDCl_3$) δ 8.54 (d, J = 8.5 Hz, 1H), 8.28 (d, J = 8.6 Hz, 1H), 8.24 (dd, J = 7.3, 1.2 Hz, 1H), 7.55 (overlapping, dd, J = 8.4, 7.4 Hz, 2H), 7.19 (d, J = 7.5 Hz, 1H), 6.26 (dd, J = 17.6, 10.5 Hz, 1H), 6.14 (dd, J = 17.8, 1.1 Hz, 1H), 5.79 (dd, J = 10.6, 1.1 Hz, 1H), 4.70 (t, J = 6.2 Hz, 1H), 2.89 (overlapping m and s, 8H), 2.45 (t, J = 7.0 Hz, 2H), 1.53 (quintet, J = 7.4 Hz, 2H), 1.42 (quintet, J = 7.3 Hz, 2H)
^{13}C NMR (126 MHz, $CDCl_3$) δ 200.2, 152.1, 136.4, 134.7, 130.5, 130.0, 129.7, 129.6, 128.5, 128.2, 123.2, 118.7, 115.2, 45.5, 43.0, 38.6, 29.1, 20.4
HRMS (ESI) *m/z* calcd. for $C_{19}H_{25}O_3N_2S$ $[M+H]^+$ 361.1580, found 361.1585
IR (ATR, neat) 3294, 2940, 2869, 2832, 2787, 1674, 1612, 1574, 1455, 1405, 1321, 1143, 1073, 791, 626, 571 cm^{-1}

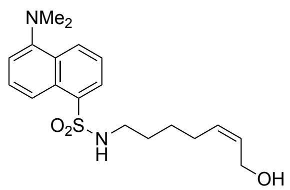

(*Z*)-5-(Dimethylamino)-*N*-(7-hydroxyhept-5-en-1-yl)naphthalene-1-sulfonamide 5-(dimethylamino)-*N*-(5-oxohept-6-en-1-yl)naphthalene-1-sulfonamide (*E*)-4-((4-(dimethylamino)phenyl)diazenyl)benzoate (S4)

A solution of $Ni(OAc)_2 \cdot 4H_2O$ (0.041 g, 0.17 mmol) in EtOH (0.85 mL) was placed under an H_2 atmosphere and immediately charged with Solution A (0.19 mL, 0.21 mmol of $NaBH_4$ and 0.021 mmol of NaOH). After 3 min, TMEDA (0.050 mL, 0.039 g, 0.33 mmol) was added. After an additional 3 min, a solution of alkyne **13** (0.150 g, 0.416 mmol) in EtOH (0.85 mL) was added. The reaction was stirred for 1 h then was quenched with H_2O (5 mL) and extracted with CH_2Cl_2 (10 mL) three times. The combined organic layers were dried over $MgSO_4$, filtered, and concentrated *in vacuo*. The resulting oil was purified via column chromatography (9% MTBE in CH_2Cl_2) to afford the desired product as a vibrant green oil (0.054 g, 36%).
Solution A was prepared as follows: NaOH (0.05 mL, 2 M in water) was added to a 1 N solution of $NaBH_4$ (0.04 g, 1.06 mmol) in EtOH (0.95 mL).

^{1}H NMR (500 MHz, $CDCl_3$) δ 8.54 (d, *J* = 8.5 Hz, 1H), 8.29 (d, *J* = 8.6 Hz, 1H), 8025 (dd, *J* = 7.3, 1.0 Hz, 1H), 7.55 (m, 2H), 7.19 (d, *J* = 7.5 Hz, 1H), 5.53 (m, 1H), 5.31 (m, 1H), 4.76 (t, *J* = 6.4 Hz, 1H), 4.07 (d, *J* = 6.9 Hz, 2H), 2.89 (overlapping m and s, 8H), 1.90 (q, *J* = 7.2 Hz, 2H), 1.37 (quintet, *J* = 7.2 Hz, 2H), 1.24 (m, 2H)
^{13}C NMR (126 MHz, $CDCl_3$) δ 152.1, 134.9, 132.2, 130.5, 130.0, 129.8, 129.1, 128.5, 123.4, 118.9, 115.3, 58.5, 45.6, 43.2, 29.0, 26.7, 26.3
HRMS (ESI) *m/z* calcd. for $C_{19}H_{27}O_3N_2S$ $[M+H]^+$ 363.1737, found 363.1742
IR (ATR, neat) 3501, 3293, 2936, 2856, 2785,1573, 1455, 1406, 1311, 1231, 1201, 1141, 1074, 910, 790, 730, 624, 571 cm^{-1}

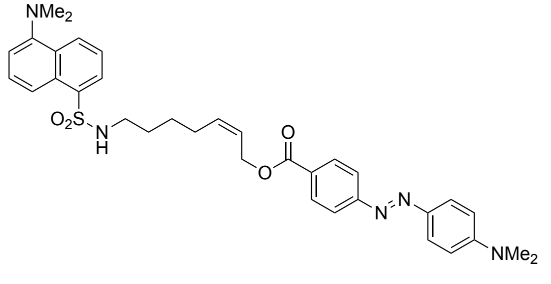

(*Z*)-7-((5-(Dimethylamino)naphthalene)-1-sulfonamido)hept-2-en-1-yl 4-((*E*)-(4-(dimethylamino)phenyl)diazenyl)benzoate (24)

A flask with crude dabcyl chloride (0.276 mmol) was purged with argon and charged with CH_2Cl_2 (1.8 mL). A solution of allylic alcohol **S4** (0.050 g, 0.14 mmol) in CH_2Cl_2 (1.0 mL) was added followed by pyridine (0.045 mL, 0.044 g, 0.56 mmol) and DMAP (0.004 g, 0.03 mmol). The reaction was stirred at rt overnight. After quenching with saturated aq. NH_4Cl (3 mL) and extracting three times with CH_2Cl_2 (5 mL), the organic layers were dried, filtered, and concentrated under reduced pressure. The crude material was purified via column chromatography (40% EtOAc in hexanes) to yield the desired product as an orange solid (0.036 g, 42%).

^{1}H NMR (500 MHz, $CDCl_3$) δ 8.54 (d, *J* = 8.5 Hz, 1H), 8.30 (d, *J* = 8.6 Hz, 1H), 8.26 (dd, *J* = 7.3, 0.9 Hz, 1H), 8.12 (d, *J* = 8.6 Hz, 2H), 7.91 (d, *J* = 9.0 Hz, 2H), 7.86 (d, *J* = 8.6 Hz, 2H), 7.18 (d, *J* = 7.0 Hz, 1H), 6.76 (d, *J* = 9.2 Hz, 2H), 5.61 (m, 1H), 5.48 (m, 1H), 4.76 (overlapping d and t, 3H), 3.11 (s, 6H), 2.90 (overlapping m and s, 8H), 2.02 (q, *J* = 7.0 Hz, 2H), 1.41 (quintet, *J* = 7.2 Hz, 2H), 1.28 (m, 2H)

^{13}C NMR (126 MHz, $CDCl_3$) δ 166.3, 156.1, 153.1, 143.9, 135.0, 134.7, 130.7, 130.6, 130.5, 130.3, 129.82, 129.78, 125.7, 124.1, 123.4, 122.1, 118.9, 115.3, 111.6, 60.8, 45.6, 43.1, 40.4, 29.8, 29.1, 27.0, 26.2
HRMS (ESI) *m/z* calcd. for $C_{34}H_{40}O_4N_5S$ $[M+H]^+$ 614.2796, found 614.2808
IR (ATR, neat) 3294, 2933, 2860, 1710, 1597, 1519,1444, 1422, 1396, 1361, 1311, 1266, 1201, 1133, 1093, 1011, 944, 909, 852, 822, 790, 775, 729, 696, 624, 571, 540, 498 cm^{-1}

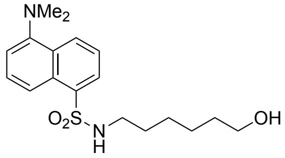

5-(Dimethylamino)-*N*-(6-hydroxyhexyl)naphthalene-1-sulfonamide (18)

6-Amino-1-hexanol (0.234 g, 2.00 mmol) was dissolved in distilled CH_2Cl_2 (6.7 mL) and cooled to 0 °C. Triethylamine (0.29 mL, 0.21 g, 2.1 mmol) was added to the solution. After 10 min, dansyl chloride (0.566 g, 2.10 mmol) was added in a single portion and the reaction was warmed to rt and stirred to completion. After 1 h, the mixture was cooled to 0 °C and quenched with saturated aqueous $NaHCO_3$ (10 mL). The aqueous layer was extracted three times with CH_2Cl_2 (10 mL) and the combined organic layers dried over $MgSO_4$, filtered, and concentrated *in* vacuo to yield an oil. The resulting oil was purified via column chromatography (50% EtOAc in hexanes) to afford the desired product as a bright green oil (0.630 g, 90%).

^{1}H NMR (500 MHz, $CDCl_3$) δ 8.53 (d, *J* = 8.5 Hz, 1H), 8.31 (d, *J* = 8.6 Hz, 1H), 8.24 (d, *J* = 7.3 Hz, 1H), 7.53 (m, 2H), 7.18 (d, *J* = 7.6 hz, 1H), 4.95 (br m, 1H, NH), 3.50 (td, *J* = 6.7, 1.7 Hz, 2H), 2.88 (overlapping m, 8H), 1.36 (m, 4H), 1.15 (m, 4H)
^{13}C NMR (126 MHz, $CDCl_3$) δ 152.1, 135.0, 130.5, 130.0, 129.8, 129.7, 128.5, 123.3, 118.9, 115.3, 62.7, 45.5, 43.2, 32.4, 29.5, 26.1, 25.1
HRMS (ESI) *m/z* calcd. for $C_{18}H_{27}O_3N_2S$ $[M+H]^+$ 351.1737, found 351.1738
IR (ATR, neat) 3510, 3292, 2935, 2860, 2789, 1574, 1455, 1406, 1309, 1140, 1061, 909, 789, 728, 623, 571 cm^{-1}

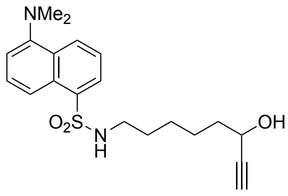

5-(Dimethylamino)-*N*-(6-hydroxyoct-7-yn-1-yl)naphthalene-1-sulfonamide (19)

A solution of DMSO (0.98 mL, 1.07 g, 13.73 mmol) in CH_2Cl_2 (30 mL) was added to a flask under Ar and cooled to –78 °C. Oxalyl chloride (0.60 mL, 0.885 g, 6.972 mmol) was added dropwise and the reaction stirred for 20 min. A solution of alcohol **18** (1.49 g, 4.25 mmol) in CH_2Cl_2 (13 mL) was added at –78 °C and stirred for an additional 20 min. Triethylamine (3.5 mL, 2.5 g, 24.7 mmol) was added dropwise and the reaction stirred for five min. The dry ice/acetone bath was removed warm the mixture to rt. The reaction was quenched with water (50 mL) and extracted with CH_2Cl_2 (3x, 50 mL). The organic layers were dried over $MgSO_4$, filtered, and concentrated to afford the crude bright green oil (**S5**) which was used after drying under vacuum in the next step without further purification.

^{1}H NMR (300 MHz, $CDCl_3$) δ 9.66 (t, *J* = 1.6 Hz, 1H), 8.54 (d, *J* = 8.5 Hz, 1H), 8.26 (m, 2H), 7.55 (m, 2H), 7.19 (d, *J* = 7.0 Hz, 1H), 4.58 (t, *J* = 6.3 Hz, 1H), 3.10 (quartet, *J* = 7.2 Hz, 1H), 2.89 (overlapping m and s, 8H), (td, *J* = 7.2, 1.5 Hz, 2H), 1.41 (m, apparent 6H), 1.20 (m, apparent 3H)

A solution of trimethylsilyl acetylene (1.51 mL, 1.04 g, 10.63 mmol) in THF (30 mL) was cooled to –78 °C and then *n*BuLi (4.30 mL, 10.6 mmol, 2.5 M solution in hexanes) was added. The reaction was stirred for 30 min then a solution of crude aldehyde **S5** in THF (13 mL) was added. The reaction was warmed to rt and stirred for 2 h. Potassium carbonate (5.88 g, 42.5 mmol) and CH_3OH (17.0 mL, 13.6 g, 425 mmol) were added, then the reaction was concentrated *in vacuo* to remove most of the THF. Additional CH_3OH (70 mL) was added to redissolve the residue, and the reaction was stirred for 2 h at rt. The heterogeneous mixture was quenched with water (100 mL) and extracted with EtOAc (3 x 100 mL). The combined organic layers were washed with brine and then dried over $MgSO_4$, filtered, and concentrated under reduced pressure. The crude mixture was purified by column chromatography (40% EtOAc in hexanes) to afford the desired product as a bright lime green oil (1.22 g, 77% over two steps).

^{1}H NMR (500 MHz, $CDCl_3$) δ 8.54 (d, *J* = 9.0 Hz, 1H), 8.29 (d, *J* = 8.7 Hz, 1H), 8.25 (d, *J* = 7.3 Hz, 1H), 7.57 (t, *J* = 8.1 Hz, 1H), 7.53 (t, *J* = 7.9 Hz, 1H), 7.19 (d, *J* = 7.6 Hz, 1H), 4.66 (t, *J* = 6.0 Hz, 1H), 4.23 (br t, 1H), 2.90 (overlapping m and s, 8H), 2.42 (d, *J* = 2.0 Hz, 1H), 1.88 (br s, 1H), 1.51 (m, 2H), 1.38 (quintet, *J* = 7.2 Hz, 2H), 1.26 (m, 2H), 1.17 (m, 2H)
^{13}C NMR (126 MHz, $CDCl_3$) δ 152.2, 134.9, 130.6, 130.0, 129.9, 129.8, 128.5, 123.4, 118.9, 115.3, 84.9, 73.1, 62.1, 45.6, 43.2, 37.4 29.5, 26.0, 24.4
HRMS (ESI) *m/z* calcd. for $C_{20}H_{27}O_3N_2S$ $[M+H]^+$ 375.1737, found 375.1736
IR (ATR, neat) 3466, 3291, 2939, 2861, 2839, 2785, 1574, 1457, 1405, 1309, 1140, 1072, 909, 790, 729, 624, 570 cm^{-1}

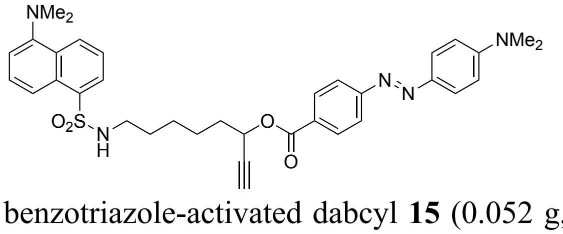

8-((5-(Dimethylamino)naphthalene)-1-sulfonamido)oct-1-yn-3-yl (*E*)-4-((4-(dimethylamino)phenyl)diazenyl)benzoate (S6)

THF (3.4 mL) was added to a flask charged with propargyl alcohol **19** (0.050 g, 0.13 mmol) and benzotriazole-activated dabcyl **15** (0.052 g, 0.14 mmol). DBU (0.021 mL, 0.021 g, 0.14 mmol) was added to the heterogenous mixture and the reaction was stirred for 41 h. The solvent was removed under reduced pressure and the crude mixture purified by column chromatography (40% EtOAc in hexanes) to afford the desired product as a red-orange solid (0.062 g, 73%).

^{1}H NMR (500 MHz, $CDCl_3$) δ 8.53 (d, *J* = 8.5 Hz, 1H), 8.29 (d, *J* = 8.7 Hz, 1H), 8.25 (dd, *J* = 7.3, 1.2 Hz, 1H), 8.12 (d, *J* = 8.7 Hz, 2H), 7.91 (d, *J* = 9.2 Hz, 2H), 7.86 (d, *J* = 8.7 Hz, 2H), 7.56 (dd, *J* = 8.5, 7.6 Hz, 1H), 7.52 (dd, *J* = 8.4, 7.3 Hz, 1H), 7.18 (d, *J* = 7.4 Hz, 1H), 6.76 (d, *J* = 9.4 Hz, 2H), 5.50 (td, *J* = 6.6, 2.1 Hz, 1H), 4.63 (t, *J* = 6.4 Hz, 1H), 3.11 (s, 6H), 2.89 (overlapping q and s, 8H), 2.47 (d, *J* = 2.2 Hz, 1H), 1.75 (m, 2H), 1.38 (m, 4H), 1.22 (m, 2H)
^{13}C NMR (126 MHz, $CDCl_3$) δ 165.3, 156.4, 153.1, 152.2, 143.9, 134.9, 130.9, 130.6, 130.0, 129.9, 129.8, 129.7, 128.5, 125.7, 123.3, 122.2, 118.8, 115.3, 111.6, 81.2, 74.0, 64.2, 45.5, 43.3, 40.4, 34.5, 29.5, 26.0, 24.5
HRMS (ESI) *m/z* calcd. for $C_{35}H_{39}N_5O_4S$ $[M+2H]^{2+}$ 313.6434, found 313.6435
IR (ATR, neat) 3290, 2934, 2861, 2785, 1714, 1598, 1520, 1396, 1363, 1311, 1264, 1135, 1092, 944, 908, 822, 790, 729, 624, 570 cm^{-1}

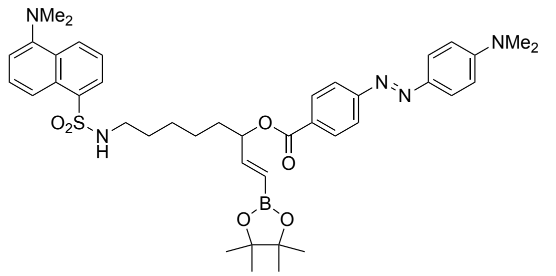

(E)-8-((5-(Dimethylamino)naphthalene)-1-sulfonamido)-1-(4,4,5,5-tetramethyl-1,3,2-dioxaborolan-2-yl)oct-1-en-3-yl 4-((E)-(4-(dimethylamino)phenyl)diazenyl)benzoate (20)

Schwartz' reagent (Cp_2ZrHCl) (0.045 g, 0.176 mmol) and THF (2.0 mL) was added to a flask charged with propargyl ester **S6** (0.551 g, 0.881 mmol) under Ar. Pinacol borane (0.19 mL, 0.17 g, 1.32 mmol) was added followed by triethylamine (0.025 mL, 0.018 g, 0.176 mmol). The reaction was heated to 60 °C and stirred overnight. The reaction was loaded directly onto a column for purification. Column chromatography (2% methyl *t*-butyl ether in CH_2Cl_2) afforded the product as a bright red oil (0.325 g, 49%).

^{1}H NMR (500 MHz, $CDCl_3$) δ 8.55 (app br s, 1H), 8.29 (br d, J = 7.6 Hz, 1H), 8.25 (d, J = 7.4 Hz, 1H), 8.12 (d, J = 8.6 Hz, 2H), 7.92 (d, J = 9.0 Hz, 2H), 7.86 (d, J = 8.6 Hz, 2H), 7.57 (t, J = 8.2 Hz, 1H), 7.52 (t, J = 8.1 Hz, 1H), 7.19 (br d, J = 7.5 Hz, 1H), 6.77 (d, J = 9.6 Hz, 2H), 6.56 (dd, J = 18.0, 5.0 Hz, 1H), 5.64 (dd, J = 18.2, 1.5 Hz, 1H), 5.47 (quartet, J = 5.8 Hz, 1H), 4.53 (t, J = 6.2 Hz, 1H), 3.12 (s, 6H), 2.88 (m, 8H), 1.59 (m, 2H), 1.36 (m, 2H), 1.20 (overlapping s and m, 16H)
^{11}B NMR (193 MHz, $CDCl_3$) δ 30.1
^{13}C NMR (126 MHz, $CDCl_3$) δ 165.6, 156.2, 153.0, 152.2, 150.1, 143.9, 134.9, 130.7, 130.6, 130.3, 130.0, 129.83, 129.76, 128.5, 125.7, 123.3, 122.1, 118.8, 115.3, 111.6, 83.5, 75.3, 45.5, 43.3, 40.1, 33.9, 29.5, 26.3, 25.0, 24.9, 24.6
HRMS (ESI) *m/z* calcd. for $C_{41}H_{53}O_6N_5BS$ $[M+H]^+$ 754.3804, found 754.3807
IR (ATR, neat) 3294, 2979, 2935, 2862, 1708, 1643, 1598, 1519, 1396, 1362, 1320, 1266, 1135, 996, 908, 729, 648, 625 cm^{-1}

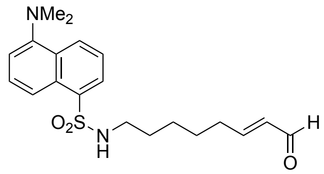

(E)-5-(dimethylamino)-*N*-(8-oxooct-6-en-1-yl)naphthalene-1-sulfonamide (23)

DMSO (23 mL) was added to boronate **20** (0.053 g, 0.070 mmol). The vial was heated to 37 °C. PBS buffer (12 mL, pH = 7.0) was added and the reaction was stirred for 15 min. H_2O_2•urea (0.198 g, 2.109 mmol) was added and the reaction was stirred overnight at 37 °C. The reaction was quenched with H_2O (20 mL) and diluted with CH_2Cl_2 (40 mL). The organic layer was washed with H_2O (20 mL) three times then dried over $MgSO_4$, filtered, and concentrated. The crude material was purified via column chromatography (3% MTBE in CH_2Cl_2) to yield **23** (0.014 g, 54%) as a clear green oil.

^{1}H NMR (600 MHz, $CDCl_3$) δ 9.46 (d, J = 7.9 Hz, 1H), 8.54 (d, J = 8.5 Hz, 1H), 8.27 (d, J = 8.7 Hz, 1H), 8.25 (dd, J = 7.3, 1.1 Hz, 1H), 7.85 (overlapping ddd, 2H), 7.19 (d, J = 7.5 Hz, 1H), 6.71 (dt, J = 15.8, 6.8 Hz, 1H), 6.02 (ddt, J = 15.6, 7.9, 1.4 Hz, 1H), 4.53 (t, J = 6.2 Hz, 1H), 2.89 (overlapping m and s, 8H), 2.15 (apparent q, J = 6.7 Hz, 2H), 1.40 (quintet, J = 7.2 Hz, 2H), 1.31 (quintet, J = 7.6 Hz, 2H), 1.21 (m, 2H)
^{13}C NMR (151 MHz, $CDCl_3$) δ 194.1, 158.2, 152.3, 134.8, 133.2, 130.6, 130.0, 129.9, 129.8, 128.6, 123.4, 118.7, 115.3, 45.5, 43.2, 32.5, 29.4, 27.3, 26.0
HRMS (ESI) *m/z* calcd. for $C_{20}H_{27}O_3N_2S$ $[M+H]^+$ 375.1737, found 375.1739
IR (ATR, neat) 3287, 2928, 2855, 1681, 1574, 1455, 1355, 1315, 1141, 1092, 1073, 974, 791, 624, 571 cm^{-1}

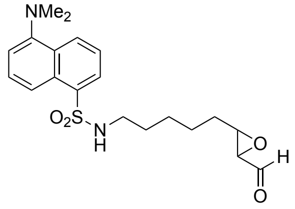

5-(Dimethylamino)-*N*-(5-(3-formyloxiran-2-yl)pentyl)naphthalene-1-sulfonamide (S7)

Boronate **20** (0.050 g, 0.066 mmol) was dissolved in DMSO (20 mL). The vial was heated to 37 °C. PBS buffer (13 mL, pH = 7.0) was added and the reaction was stirred for 15 min. H_2O_2•urea (0.187 g, 1.99 mmol) was added. The reaction was stirred overnight at 45 °C and monitored for both enal and epoxy-aldehyde formation. The reaction was quenched with H_2O (20 mL) and diluted with CH_2Cl_2 (40 mL). The organic layer was washed with H_2O (20 mL) three times then dried over $MgSO_4$, filtered, and concentrated. The crude material was purified via column chromatography (4% MTBE in CH_2Cl_2) to yield the epoxide (0.004 g, 16%) as a light-yellow fluorescent oil.

^{1}H NMR (600 MHz, $CDCl_3$) δ 8.98 (d, *J* = 6.2 Hz, 1H), 8.55 (d, *J* = 8.5 Hz, 1H), 8.28 (d, *J* = 8.7 Hz, 1H), 8.26 (d, *J* = 7.2 Hz, 1H), 7.57 (t, *J* = 8.1 Hz, 1H), 7.53 (t, *J* = 7.9 Hz, 1H), 7.19 (d, *J* = 7.5 Hz, 1H), 4.55 (t, *J* = 6.2 Hz, 1H), 3.09 (m, 1H), 3.05 (dd, *J* = 6.3, 1.9 Hz, 1H), 2.90 (overlapping m and s, 8H), 1.53 (m, 1H), 1.42 (m, 3H), 1.26 (m, 6H)
^{13}C NMR (151 MHz, $CDCl_3$) δ 198.4, 152.2, 134.8, 130.6, 129.9, 129.8, 128.6, 123.4, 118.8, 115.3, 59.1, 56.6, 45.6, 43.2, 31.0, 29.5, 26.1, 25.3
HRMS (ESI) *m/z* calcd. for $C_{20}H_{27}N_2O_4S$ $[M+H]^+$ 391.1686, found 391.1688
IR (ATR, neat) 3298, 2937, 2860, 2832, 2782, 1725, 1575, 1455, 1318, 1143, 1070, 792, 626, 572 cm^{-1}

^{1}H NMR Release Experiments and Spectra

General Procedure for Monitoring Release via ^{1}H NMR Spectroscopy.
The NMR spectrometer (600 MHz) was programmed to acquire a spectrum every 32 scans (2 minutes, 5 seconds) with a relaxation time of 7 seconds and was locked to CD_3CN. Phosphate buffer solutions were prepared with D_2O according to Gomori[5] and the pH was measured before each use. Each sample was charged with 1,2-dimethoxyethane (5.0 µL, 0.0077 mmol, 1.5 M in CD_3CN) or toluene (5 µL, 0.0075 mmol, 1.5 M in d6-DMSO) as the internal standard. All experiments were run at 37 °C and were modeled after Hanna's protocol.[6]

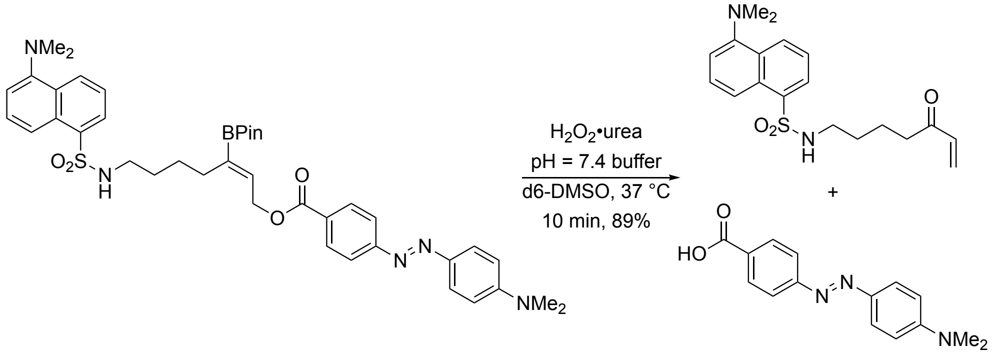

Scheme S1. Oxidative cleavage of **16**.

Toluene (internal standard, 5 µL) was added to boronic ester **16** (0.0009 mmol) in d6-DMSO (500 µL). An initial ^{1}H NMR spectrum was taken to obtain baseline integration values relative to the internal standard. A solution of phosphate buffer (50 µL, 0.1 M in D_2O, pH = 7.4) in D_2O (100 µL) was added to the NMR tube. A second ^{1}H NMR spectrum was taken to observe hydrolysis of the boronic ester to the boronic acid without undesired release. A solution of H_2O_2•urea (0.0025 g, 0.027 mmol) in D_2O (150 µL) was added and the reaction was mixed by inversion twice. The release of enone **21** was monitored by ^{1}H NMR spectroscopy over time, using integration values to quantity product formation and starting material consumption.

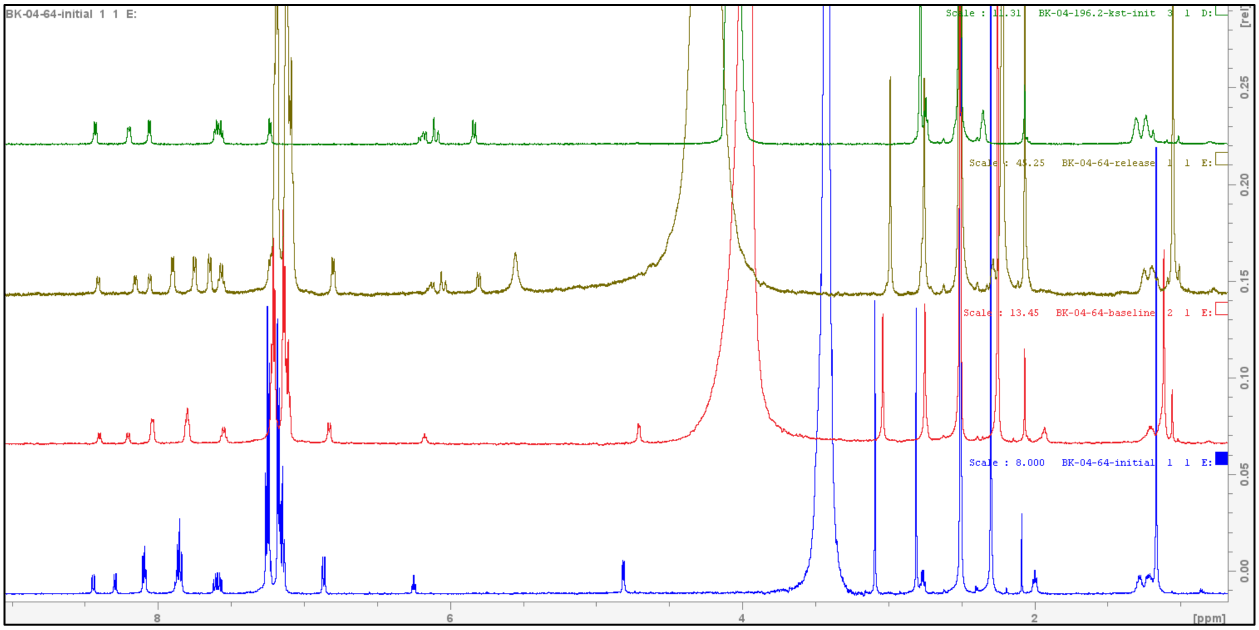

Figure S1. Full spectrum of the oxidative cleavage of **16**. Blue: spectrum of **16** and toluene (internal standard) in d6-DMSO. Red: **16** upon addition of buffer and D_2O. Yellow: spectrum after the addition of H_2O_2•urea after 10 min. Green: **21** in DMSO and buffer in the presence of H_2O_2•urea.

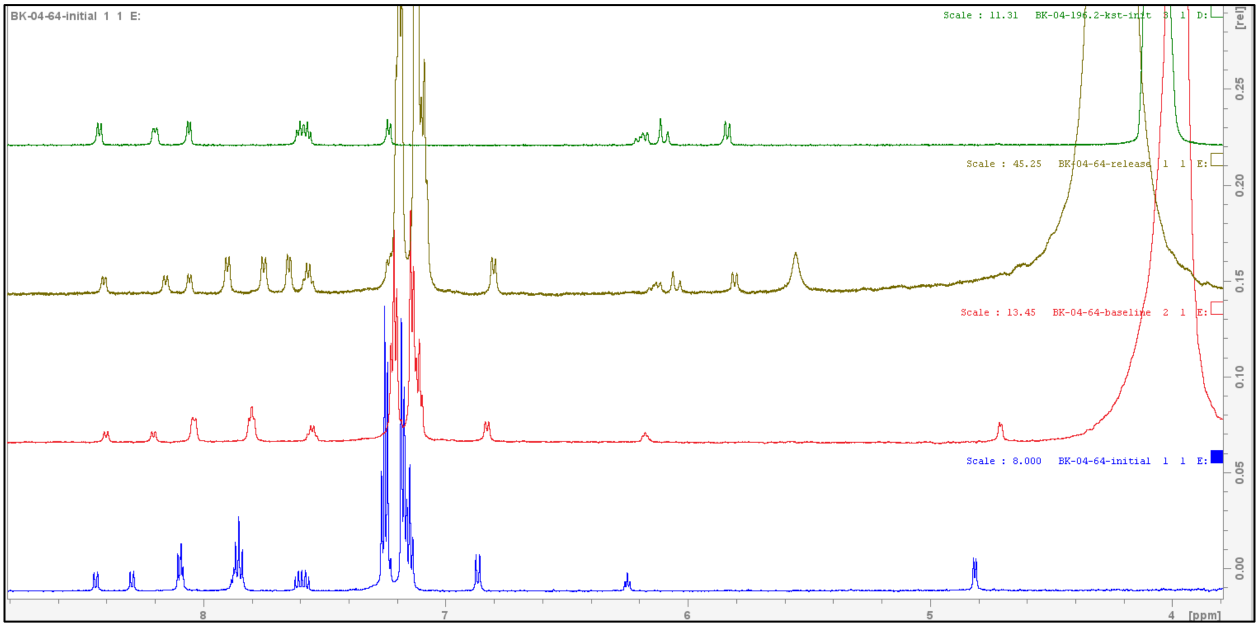

Figure S2. Downfield region of Figure S1. Blue: spectrum of **16** and 1,2-toluene (internal standard) in d6-DMSO. Red: **16** upon addition of buffer and D_2O. Yellow: spectrum after the addition of H_2O_2•urea after 10 min. Green: **22** in DMSO and buffer in the presence of H_2O_2•urea.

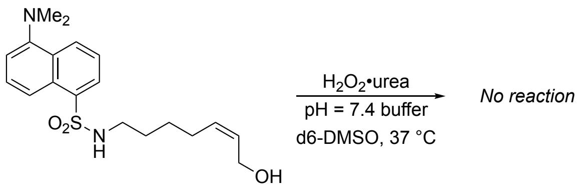

Scheme S2. Lack of reactivity from the negative control.

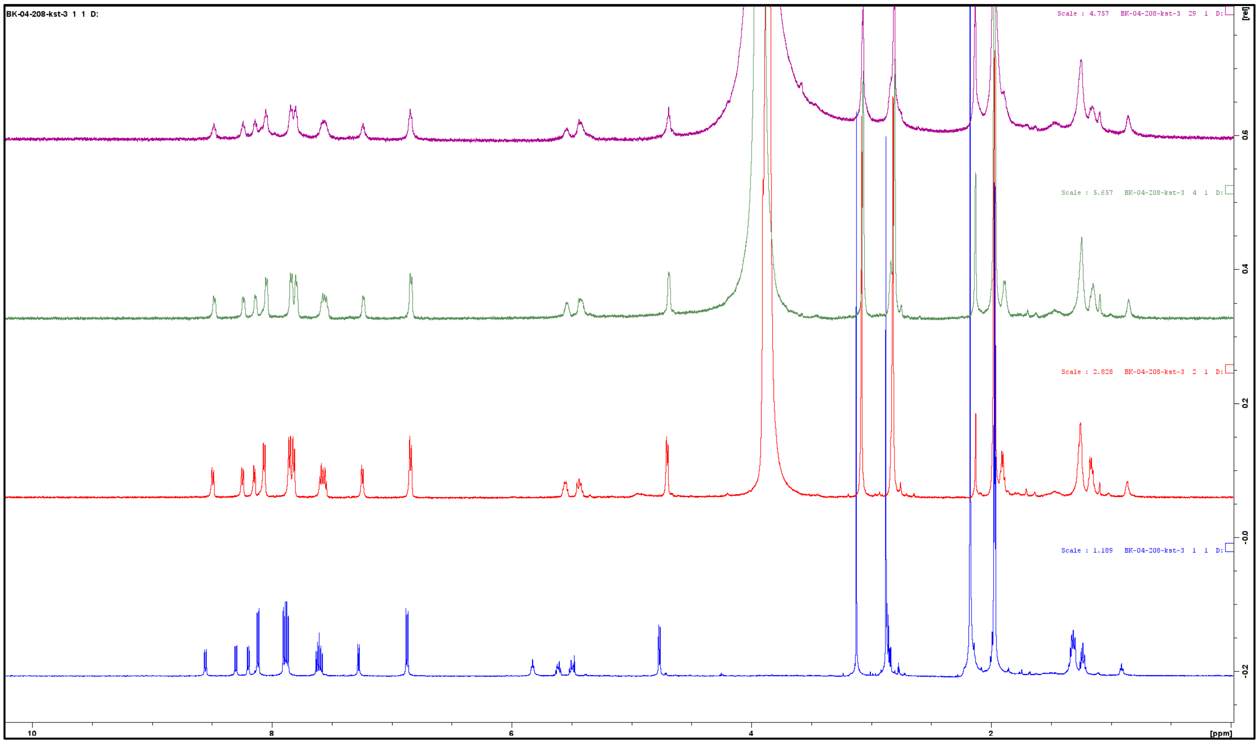

Figure S3. Full spectrum of the lack of reaction of **24**. Blue: Spectrum of substrate **24** in d6-DMSO. Red: **24** upon the addition of buffer and D_2O. Green: Spectrum upon the addition of H_2O_2 after 15 min. Purple: addition of H_2O_2 after 1 h.

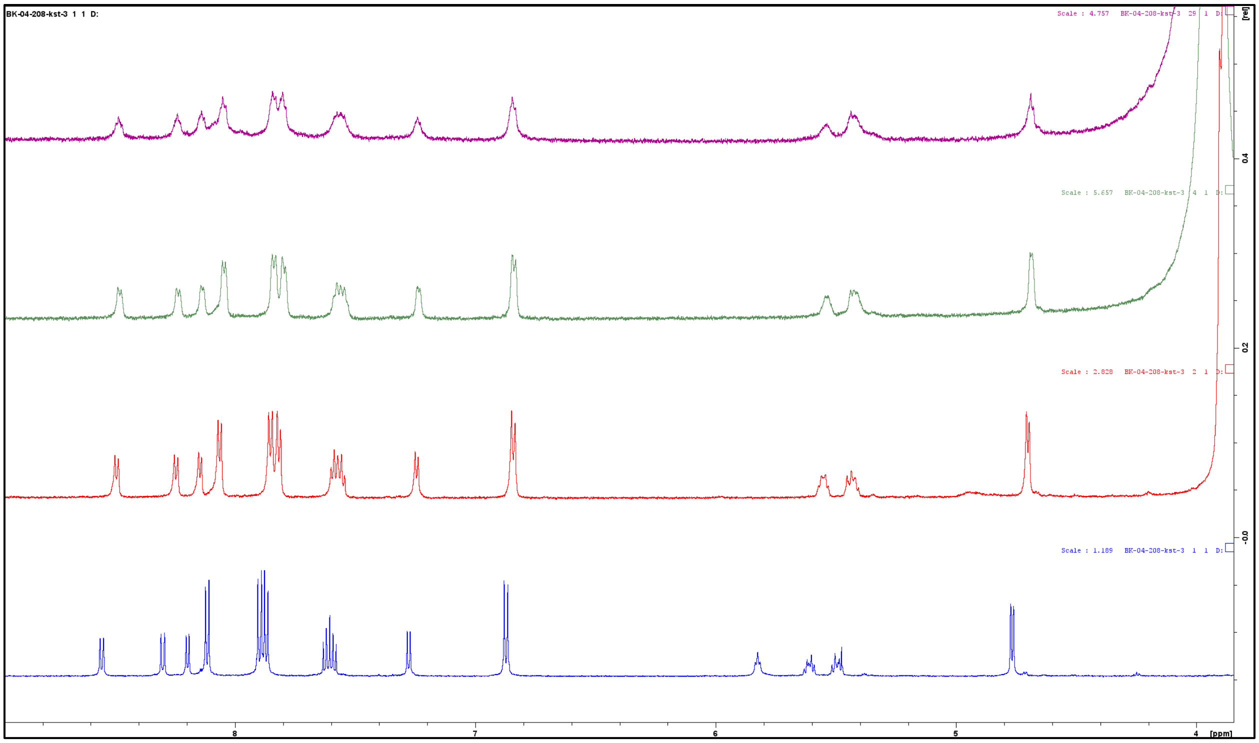

Figure S4. Downfield region of Figure S3. Blue: Spectrum of substrate **24** in d6-DMSO. Red: **24** upon the addition of buffer and D_2O. Green: Spectrum upon the addition of H_2O_2 after 15 min. Purple: addition of H_2O_2 after 1 h.

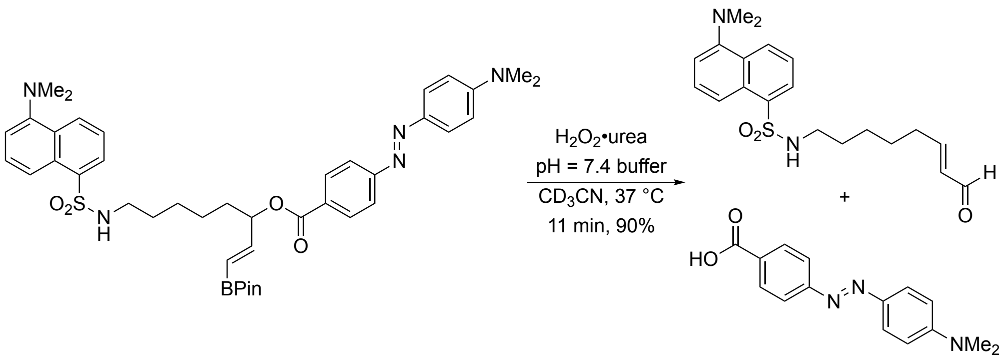

Scheme S2. Oxidative cleavage of **20**.

1,2-dimethoxyethane (internal standard, 5 μL) was added to boronic ester **20** (0.0012 mmol) in CD_3CN (500 μL). An initial ^1H NMR spectrum was taken to obtain baseline integration values relative to the internal standard. A solution of phosphate buffer (50 μL, 0.1 M in D_2O, pH = 7.4) in D_2O (100 μL) was added to the NMR tube. A second ^1H NMR spectrum was taken to observe hydrolysis of the boronic ester to the boronic acid without undesired release. A solution of H_2O_2•urea (0.0035 g, 0.037 mmol) in D_2O (150 μL) was added and the reaction was mixed by inversion twice. The release of enal **22** was then monitored by ^1H NMR spectroscopy over time, using integration values to quantity product formation and starting material consumption.

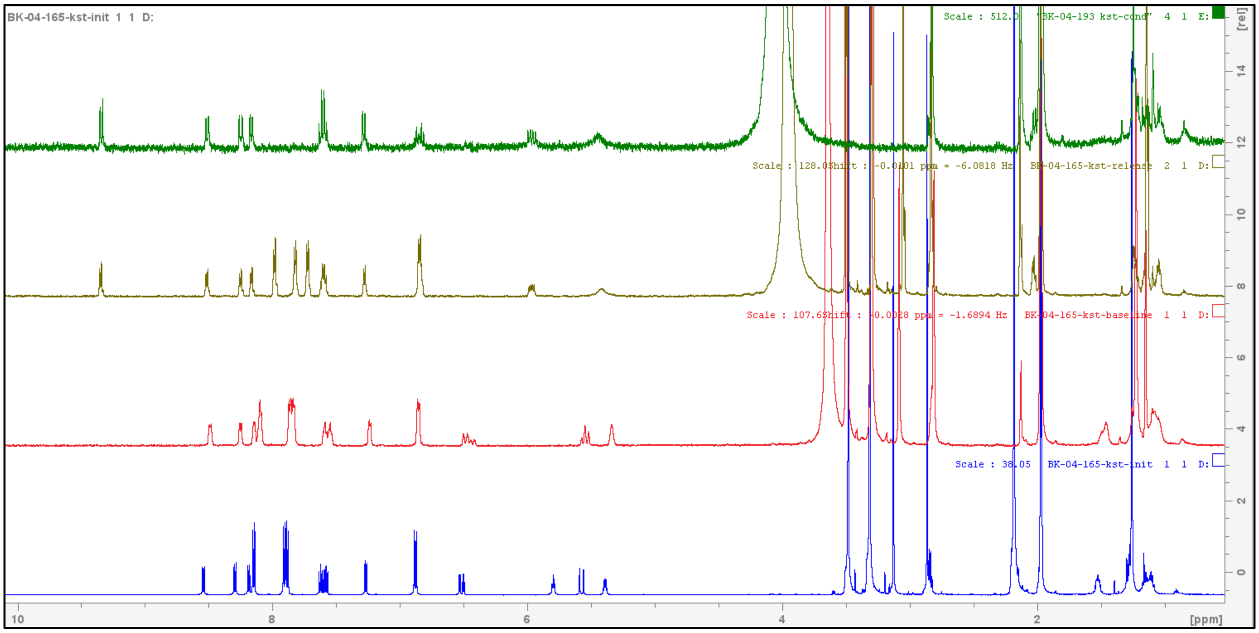

Figure S5. Full spectrum of the oxidative cleavage of **20**. Blue: spectrum of **20** and 1,2-dimethoxy ethane (internal standard) in CD_3CN. Red: **20** upon addition of buffer and D_2O. Yellow: spectrum after the addition of H_2O_2•urea after 11 min. Green: **23** in CD_3CN and buffer in the presence of H_2O_2•urea.

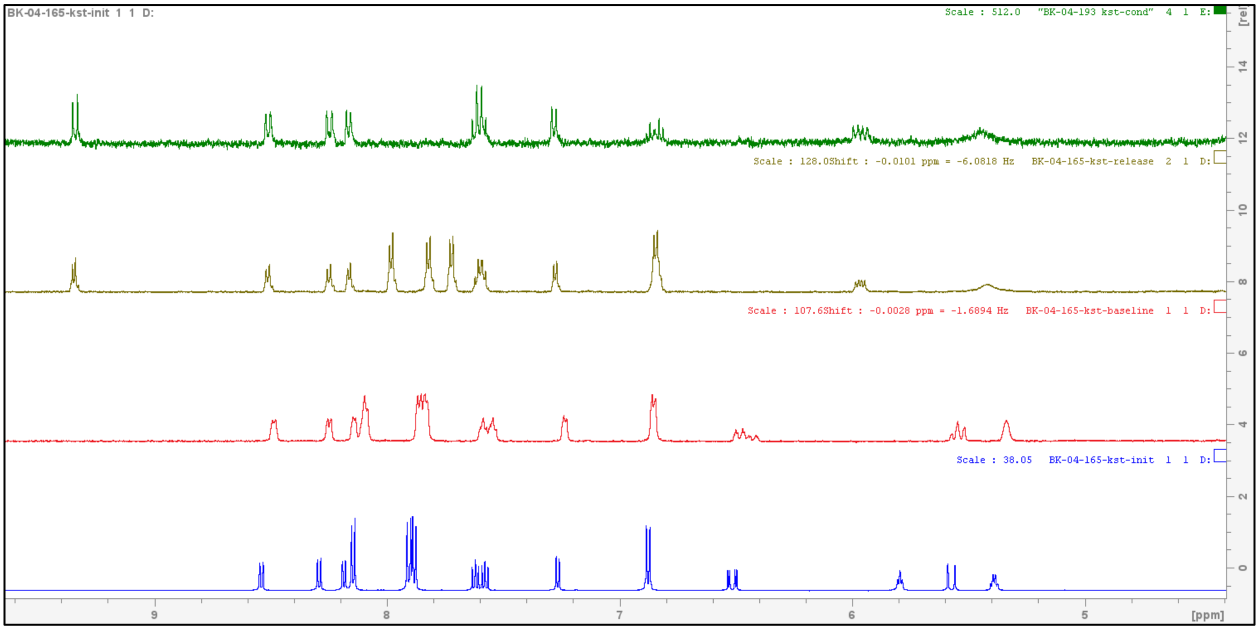

Figure S6. Upfield region of Figure S5. Blue: spectrum of **20** and 1,2-dimethoxy ethane (internal standard) in d6-DMSO. Red: **20** upon addition of buffer and D_2O. Yellow: spectrum after the addition of H_2O_2•urea after 10 min. Green: **23** in CD_3CN and buffer in the presence of H_2O_2•urea.

Fluorescence Assays

All substrates (boronate **16**, negative control **24**, enone **21**, DABCYL-acid **22**, boronate **20**, enal **23**, and epoxide **S6**) were prepared as 1 mM stock solutions in DMSO. Hydrogen peroxide was prepared as a 30 mM stock in PBS buffer (pH 7.4). All experiments were run in triplicate at 37 °C using a Tecan M1000 instrument in a Griener Flat Black 96-well plate.

General Procedure for Plate Reader-Based Assays

Wells were charged with a solution of substrate (40 µL, 1 mM in DMSO) and diluted with DMSO to a total volume of 128 µL. PBS buffer (32 µL) was then added, and the plate was warmed to 37 °C in the plate reader. H_2O_2•urea (40 µL, 30 mM in PBS buffer) or additional PBS buffer (40 µL) was added to each well for a final substrate concentration of 200 µM and total volume of 200 µL. Fluorescence intensity data was then collected at 1-minute intervals over 4 or 8 hours.

For the quenching experiment, wells were charged with a mixture of boronate **16** (21.8 µL, 1mM in DMSO) and enone **21** (18.2 µL, 1mM in DMSO) to mimic the respective concentrations at the time where the slope changes during the oxidation and the experiment continued as reported in the general procedure.

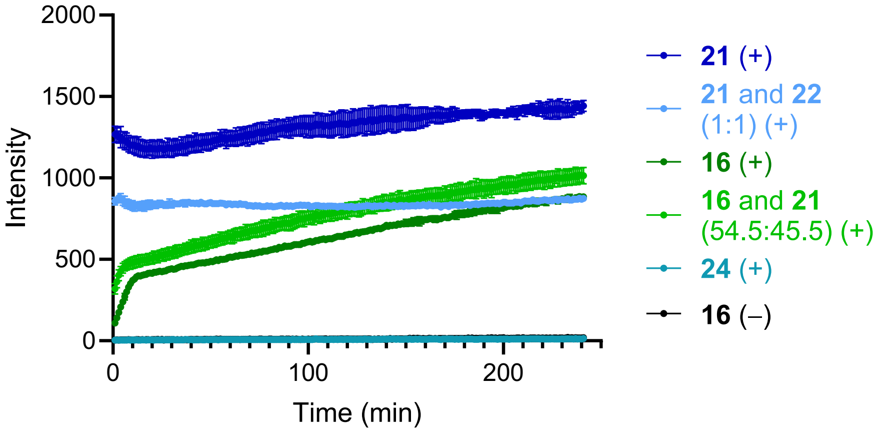

Figure S7. Oxidative breakdown of **16** with quenching experiment (excitation 351 nm, emission 538 nm).

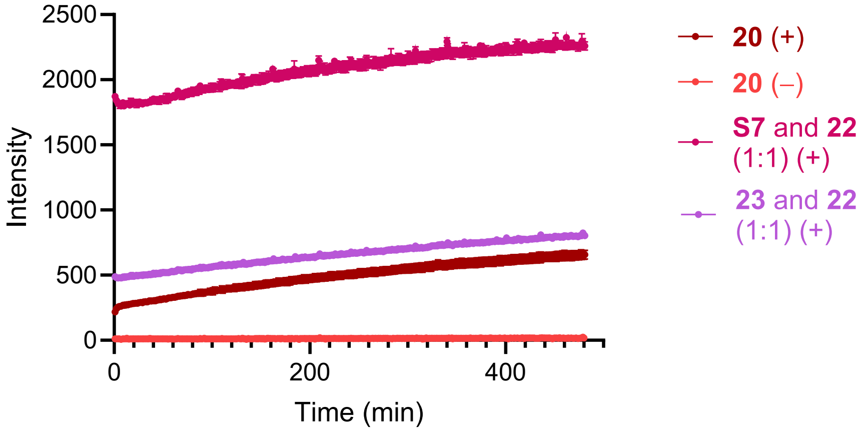

Figure S8. Oxidative breakdown of 2nd generation linker **20** (excitation/emission 335/534 nm).

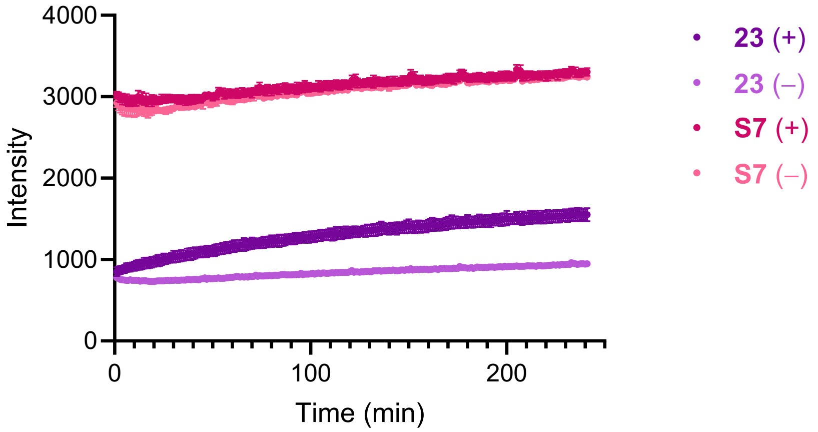

Figure S9. Reactivity of enal **23** and epoxide **S7** with H_2O_2 (excitation/emission 335/534 nm).

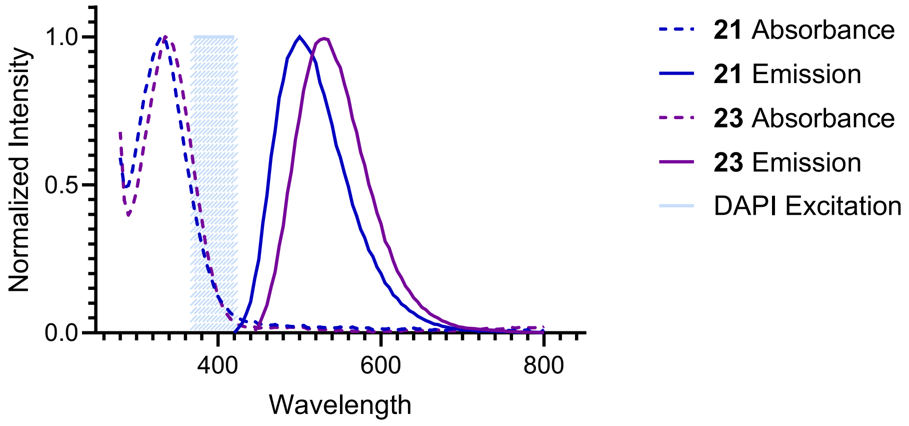

Figure S10. Enone **21** and enal **23** excitation and emission data at 200 µM.

Cellular Studies

Cell Culture Maintenance

All cell culture experiments were performed in a sterile laminar flow hood. HEK293T, U87, and B16 cells were purchased from ATCC, Manassas, VA USA. HEK and U87 cell lines were incubated at 37 °C and 5% CO_2 in Dulbecco's modified Eagle's medium (DMEM, Hyclone). B16 cell lines were grown at 37 °C and 5% CO_2 in Roswell Park Memorial Institute (RPMI) 1640 medium. Both types of media were supplemented with 10% (v/v) fetal bovine serum (FBS, Seradigm) and 1% (v/v) penicillin/streptomycin (p/s). Cells were used between passage number 4 and 28. Cell lines were tested for mycoplasma contamination using a PCR Mycoplasma detection kit (Thermo Scientific Chemicals) every 4 months. Dimethylsulfoxide (DMSO) was purchased from Sigma-Aldrich and hydrogen peroxide (30%) was purchased from Fisher Scientific.

Fluorescence Analysis in Noncancerous (HEK) and Cancerous (U87, B16) Cell Lines

Cell (10,000 per well) were seeded into a 96-well plate (Greiner Bio-One, 10,000 cells/well) in 100 µL of media (HEK and U87 in DMEM, B16 in RPMI) with 10% FBS and 1% p/s and incubated for 48 hours at 37 °C with 5% CO_2. All substrates were prepared as 1 mM solutions in DMSO and aq. H_2O_2 was prepared as a 1 mM solution in PBS buffer (pH 7.4). When cells reached 80-90% confluency, the media in each well was removed and replaced with 200 µL fresh media containing 20 mM of the indicated substrate (4 µL of 1mM). After a 45 minute incubation period, the media was removed and cells were washed twice with 180 µL of PBS buffer to remove excess substrate. Either PBS buffer with and without 100 µM H_2O_2 (20 µL of 1 mM) were added to the cells and incubated at 37 °C with 5% CO_2 for 45 minutes. Cells were then directly imaged using a Zeiss Axio Observer Z1 microscope with an Andor Zyla 4.2 camera, LD Plan-Neofluar 40x/0.6 objective and DAPI (Chroma filter 49028, ex. ET 395/25, em. 460/50) filter set. At least three fields of view (FOV) were recorded for each condition.

Images were exported in TIFF format using SlideBook. Image processing, analysis, and quantification was performed in ImageJ. Images were processed by using the subtract background function in ImageJ, then pseudocolored cyan to represent fluorescence observed under the DAPI filter. The "threshold" tool in ImageJ to select regions of interest (ROI, n = 3) and the mean fluorescent intensity (F) was calculated using the measure tool in ImageJ. ROIs containing no cells were used to calculate background mean fluorescent intensity and were subsequently background subtracted from the mean fluorescent intensities of the cellular ROIs. The background-subtracted fluorescent intensity was normalized against the mean fluorescent intensity calculated for the relevant negative control experiments to allow for comparison of endogenous and exogenous H_2O_2 treated cells. Statistical analysis was conducted via an unpaired t-test with Welch correction in the GraphPad Prism Software.

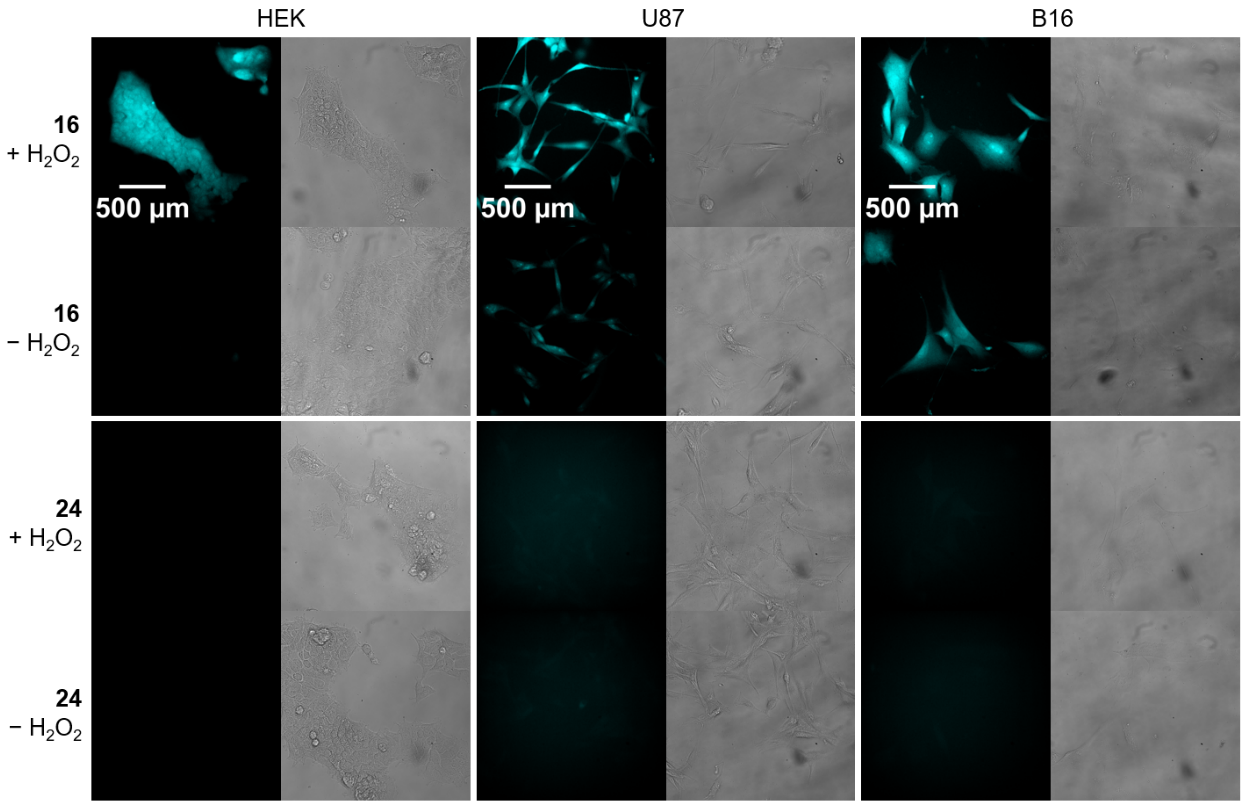

Figure S11. Microscopy images of cells treated with **16** or **24** in the presence of endogenous or exogenous H_2O_2.

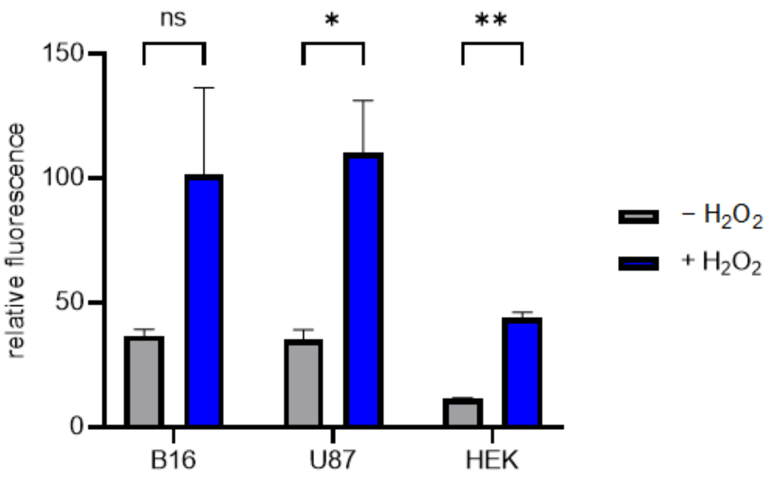

Figure S12. Relative fluorescence intensity across cell lines. Bars represent averages and error bars represent standard deviations, * = $P < 0.05$, ** = $P < .001$, ns = not statistically different.

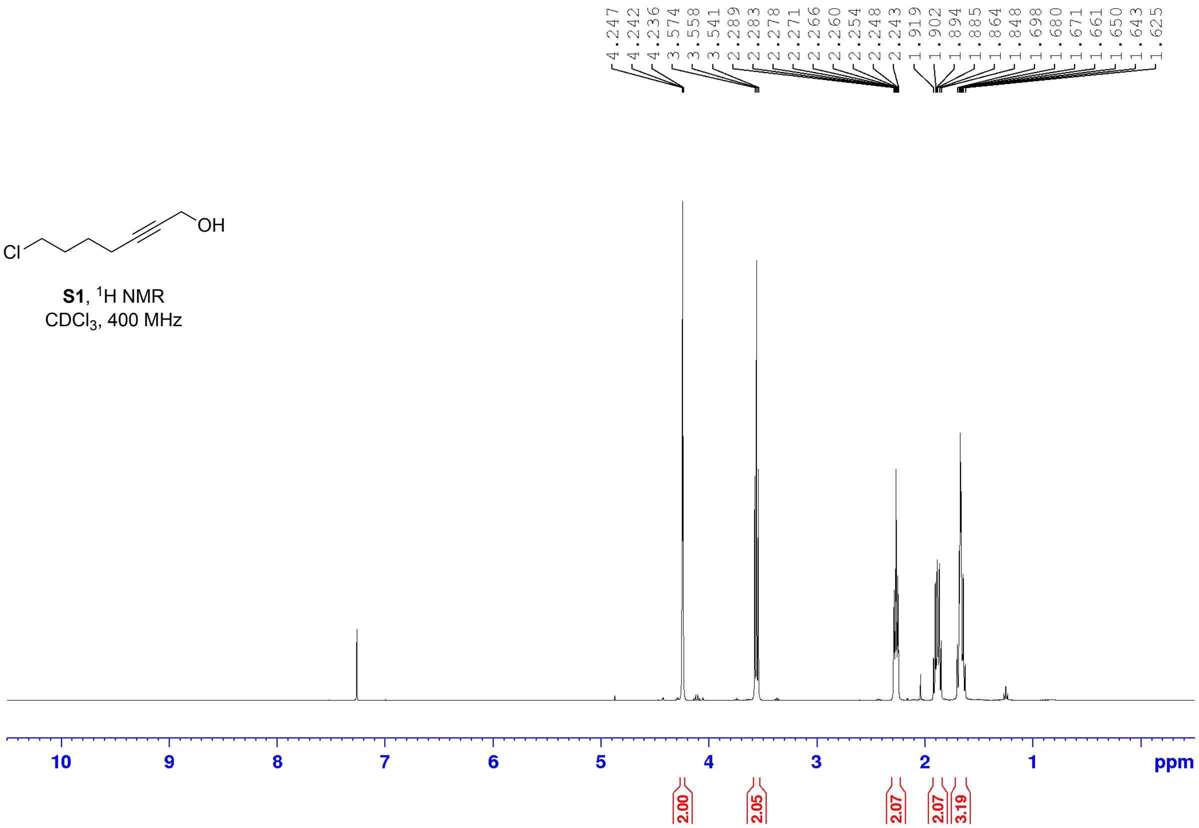
4.247
4.242
4.236
3.574
3.558
3.541
2.289
2.283
2.278
2.271
2.266
2.260
2.254
2.248
2.243
1.919
1.902
1.894
1.885
1.864
1.848
1.698
1.680
1.671
1.661
1.650
1.643
1.625
OH
Cl
S1, 1H NMR
CDCl3, 400 MHz
10 9 8 7 6 5 4 3 2 1 ppm
2.00
2.05
2.07
2.07
3.19

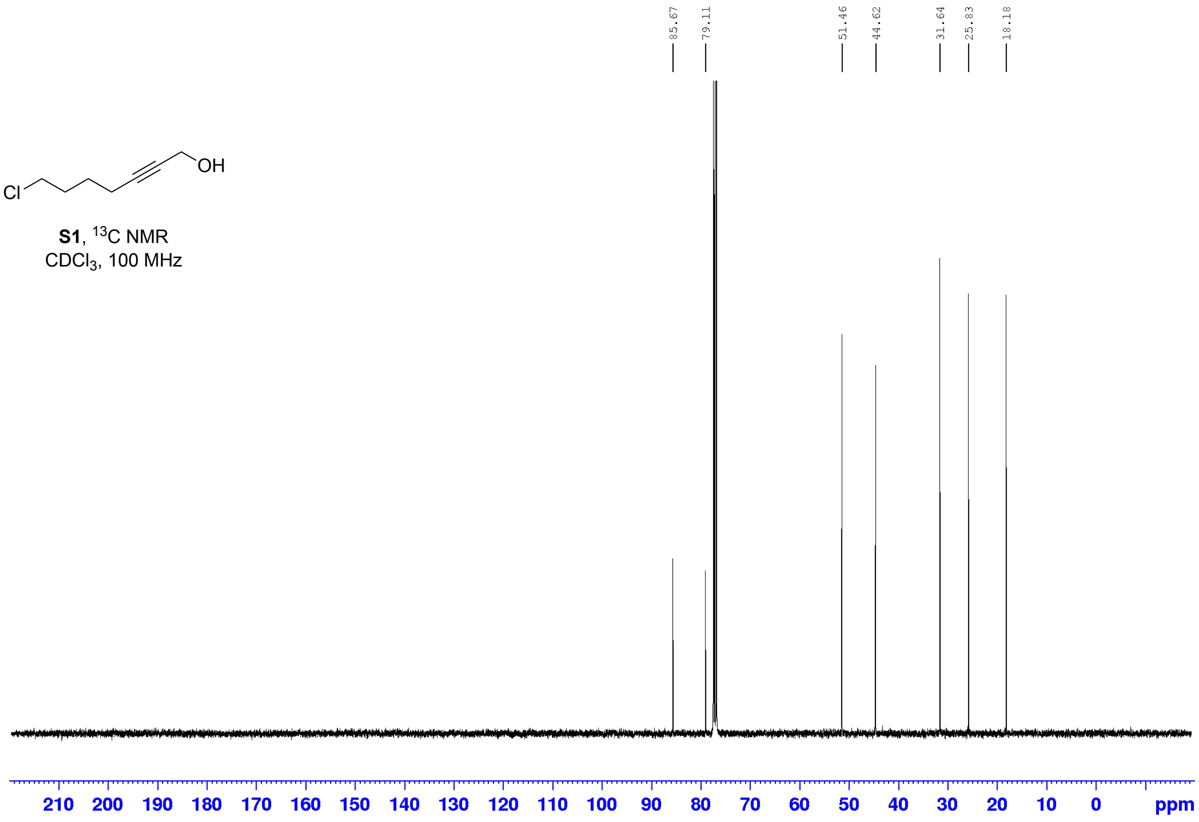
85.67
79.11
51.46
44.62
31.64
25.83
18.18
OH
Cl
S1, 13C NMR
CDCl3, 100 MHz
210 200 190 180 170 160 150 140 130 120 110 100 90 80 70 60 50 40 30 20 10 0 ppm

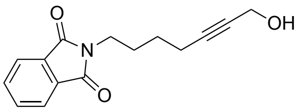

12, ^{1}H NMR
$CDCl_3$, 500 MHz

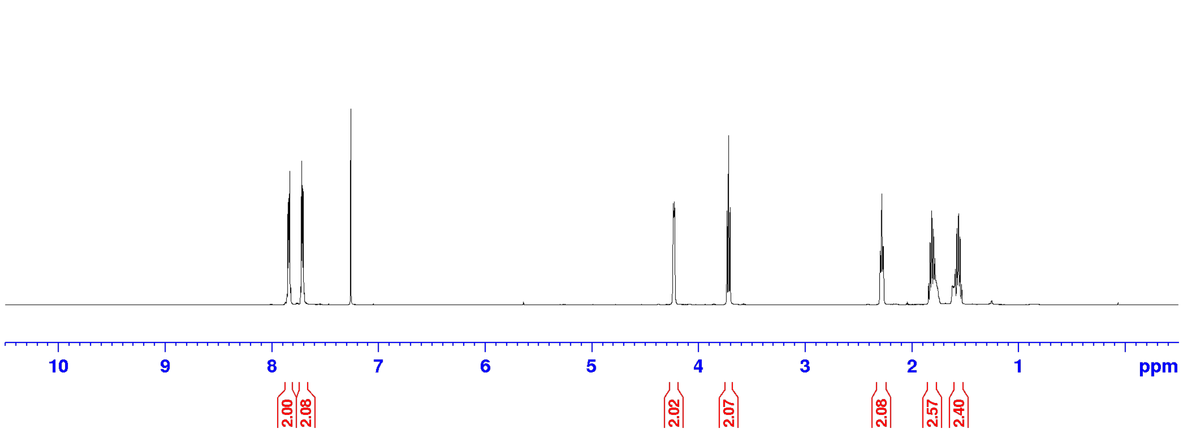

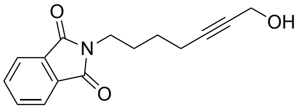

12, ^{13}C NMR
$CDCl_3$, 126 MHz

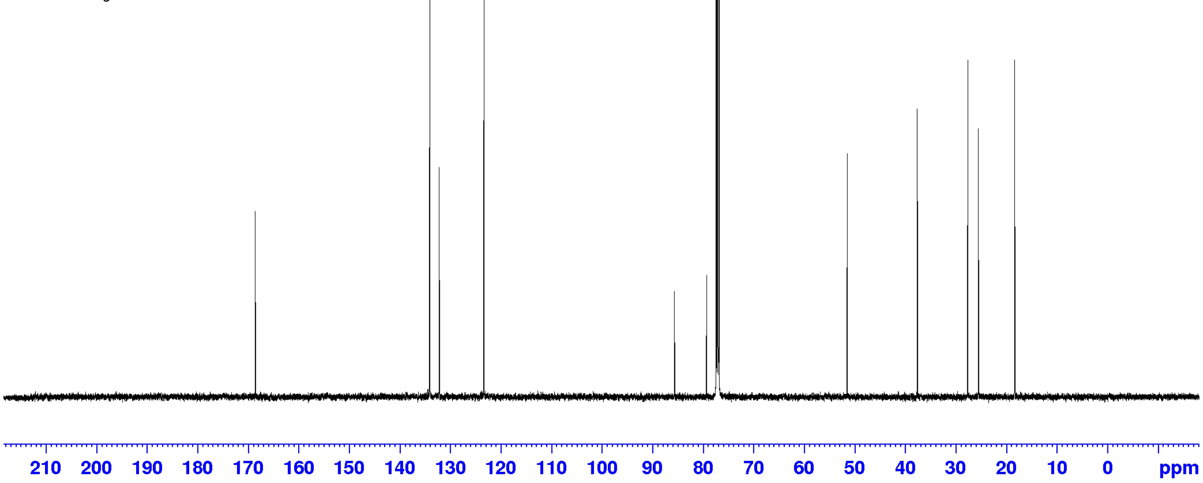

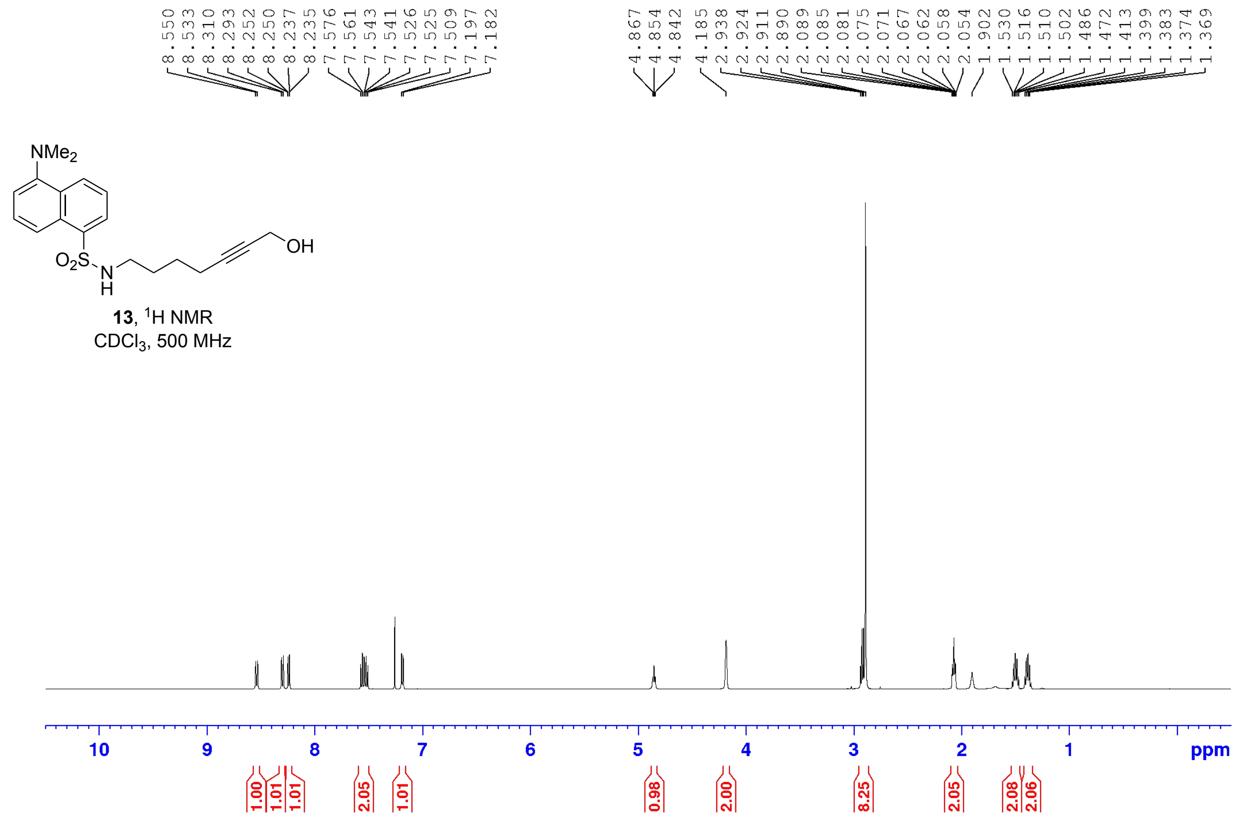

13, ^{1}H NMR
$CDCl_3$, 500 MHz

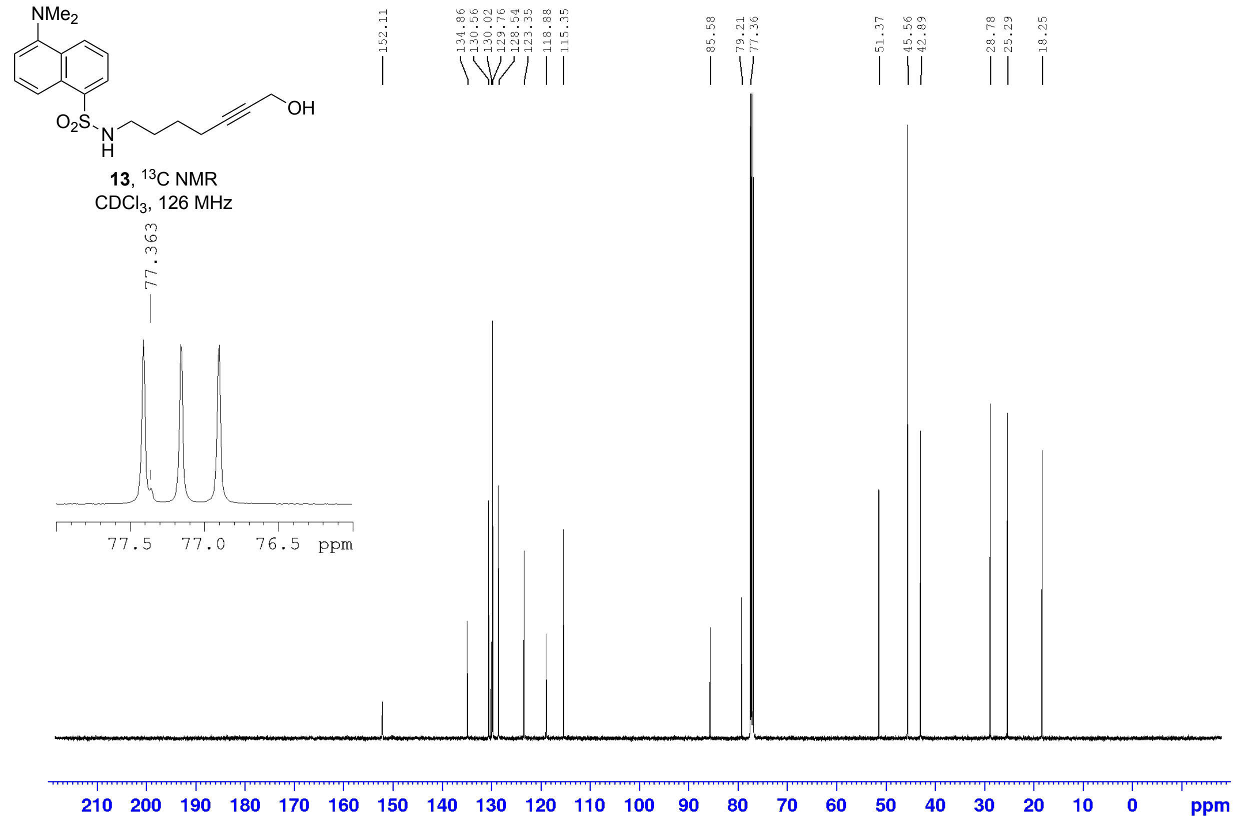

13, ^{13}C NMR
$CDCl_3$, 126 MHz

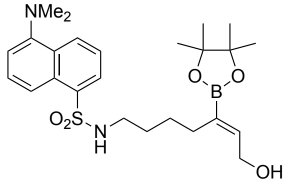

14, ^{1}H NMR
$CDCl_3$, 500 MHz

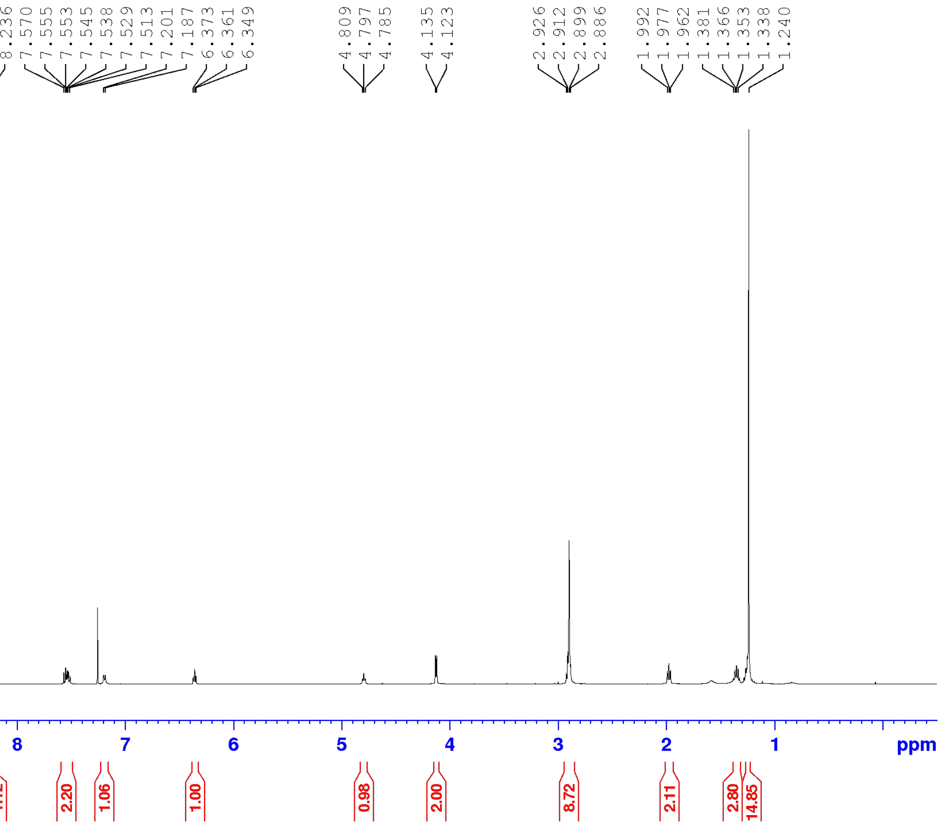

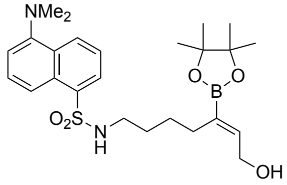

14, ^{13}C NMR
$CDCl_3$, 126 MHz

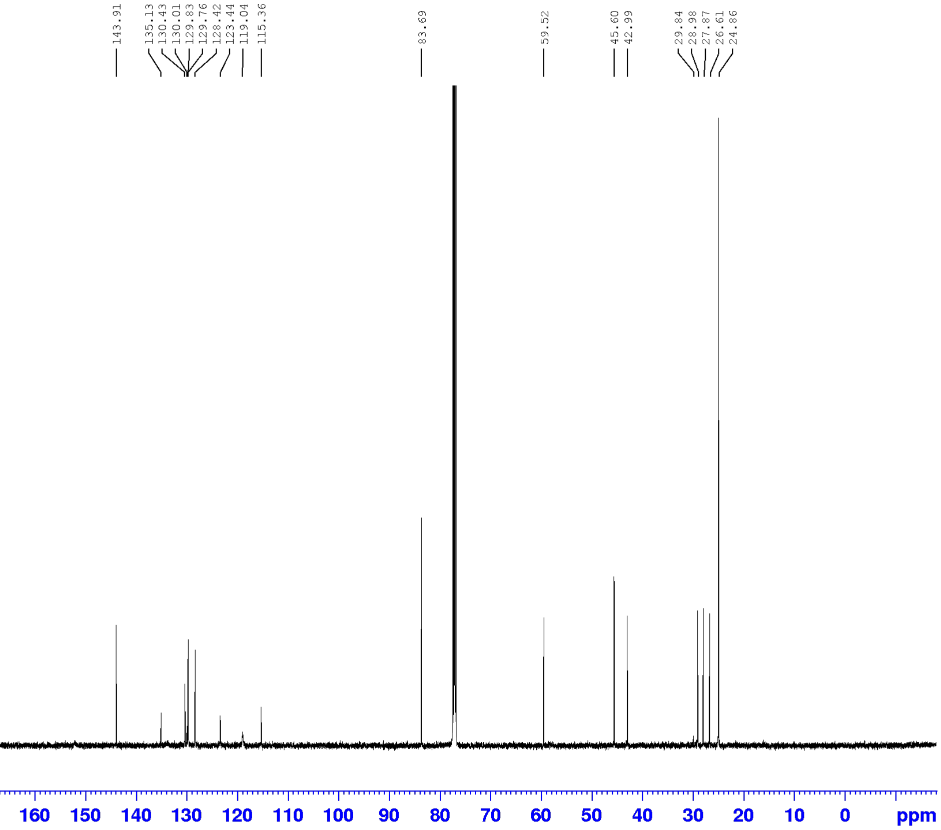

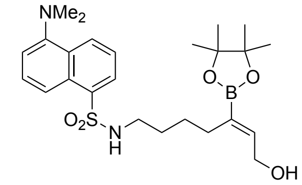

14, ^{11}B NMR
$CDCl_3$, 160 MHz

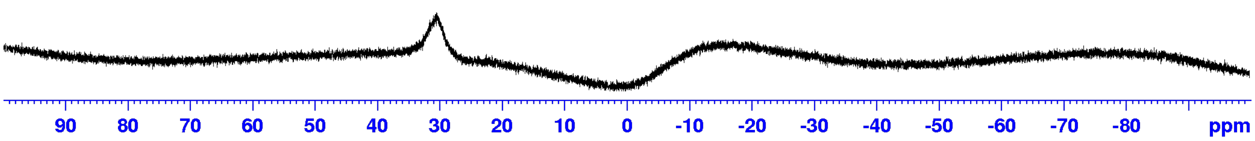

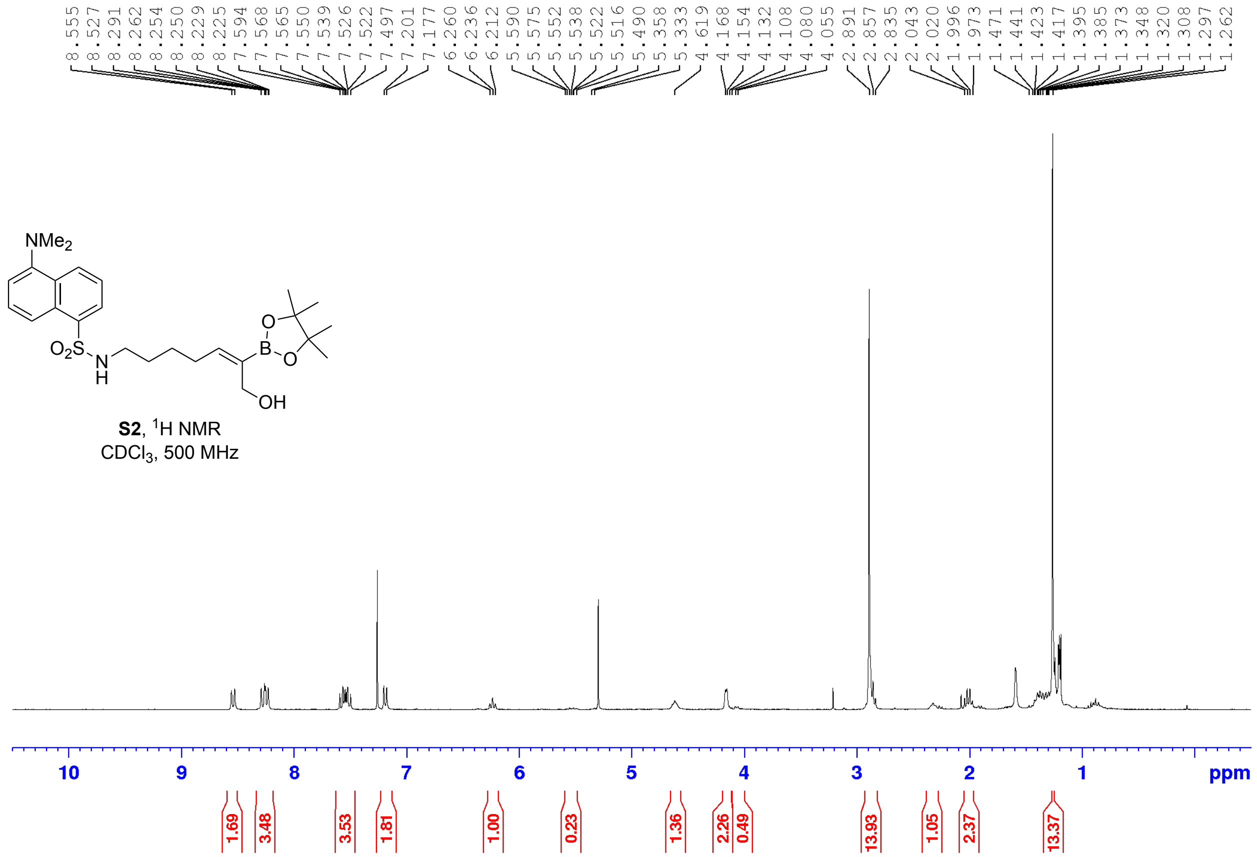
NMe2
O2S
N
H
B
O
O
OH
S2, 1H NMR
CDCl3, 500 MHz
ppm

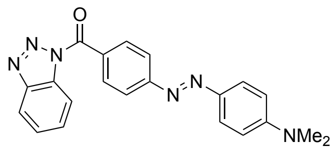

15, ^{1}H NMR
$CDCl_3$, 300 MHz

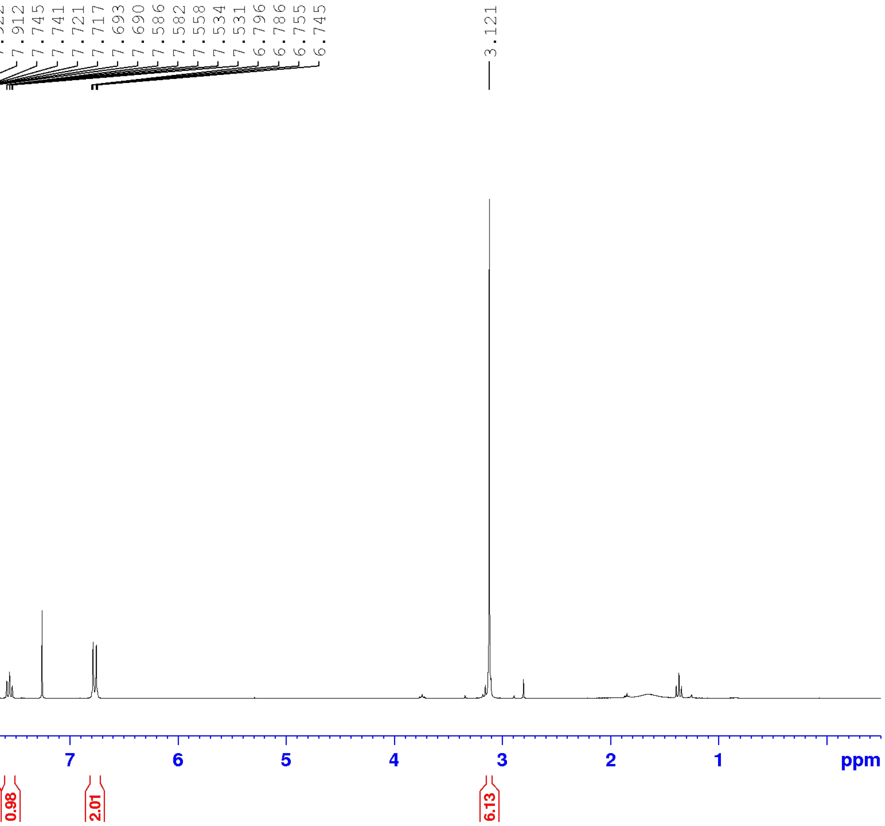

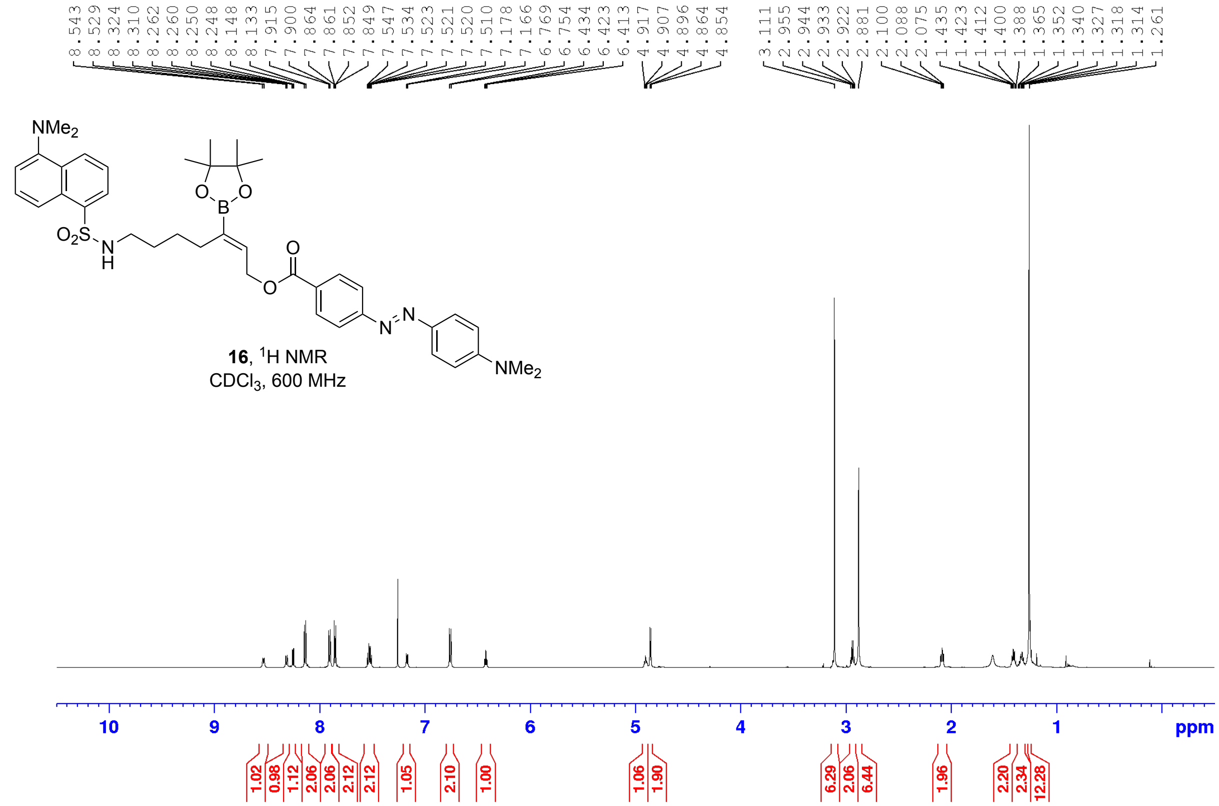
NMe2
O2S
N
H
O
B
O
O
O
N
N
NMe2
16, 1H NMR
CDCl3, 600 MHz
8.543 8.529 8.324 8.310 8.262 8.260 8.250 8.248 8.148 8.133 7.915 7.900 7.864 7.861 7.852 7.849 7.547 7.534 7.523 7.521 7.520 7.510 7.178 7.166 6.769 6.754 6.434 6.423 6.413 4.917 4.907 4.896 4.864 4.854 3.111 2.955 2.944 2.933 2.922 2.881 2.100 2.088 2.075 1.435 1.423 1.412 1.400 1.388 1.365 1.352 1.340 1.327 1.318 1.314 1.261
1.02 0.98 1.12 2.06 2.06 2.12 2.12 1.05 2.10 1.00 1.06 1.90 6.29 2.06 6.44 1.96 2.20 2.34 12.28
10 9 8 7 6 5 4 3 2 1 ppm

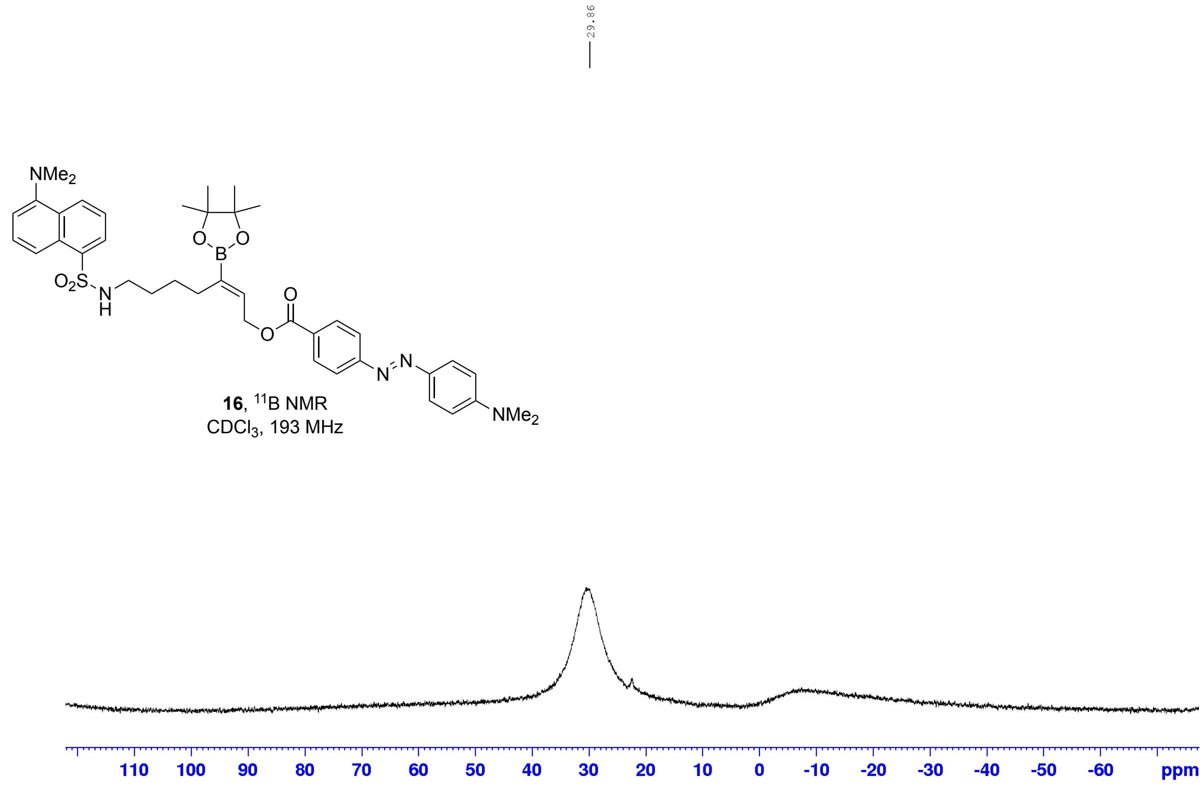
29.86
NMe2
O2S
N
H
O
B
O
O
O
N
N
NMe2
16, 11B NMR
CDCl3, 193 MHz
110 100 90 80 70 60 50 40 30 20 10 0 -10 -20 -30 -40 -50 -60 ppm

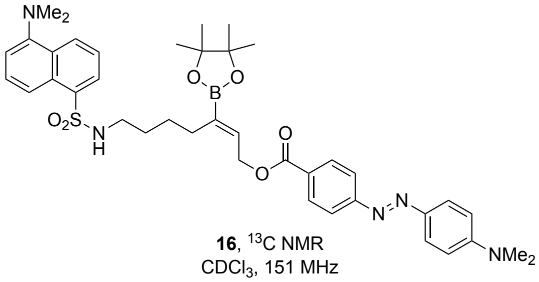

16, ^{13}C NMR
$CDCl_3$, 151 MHz

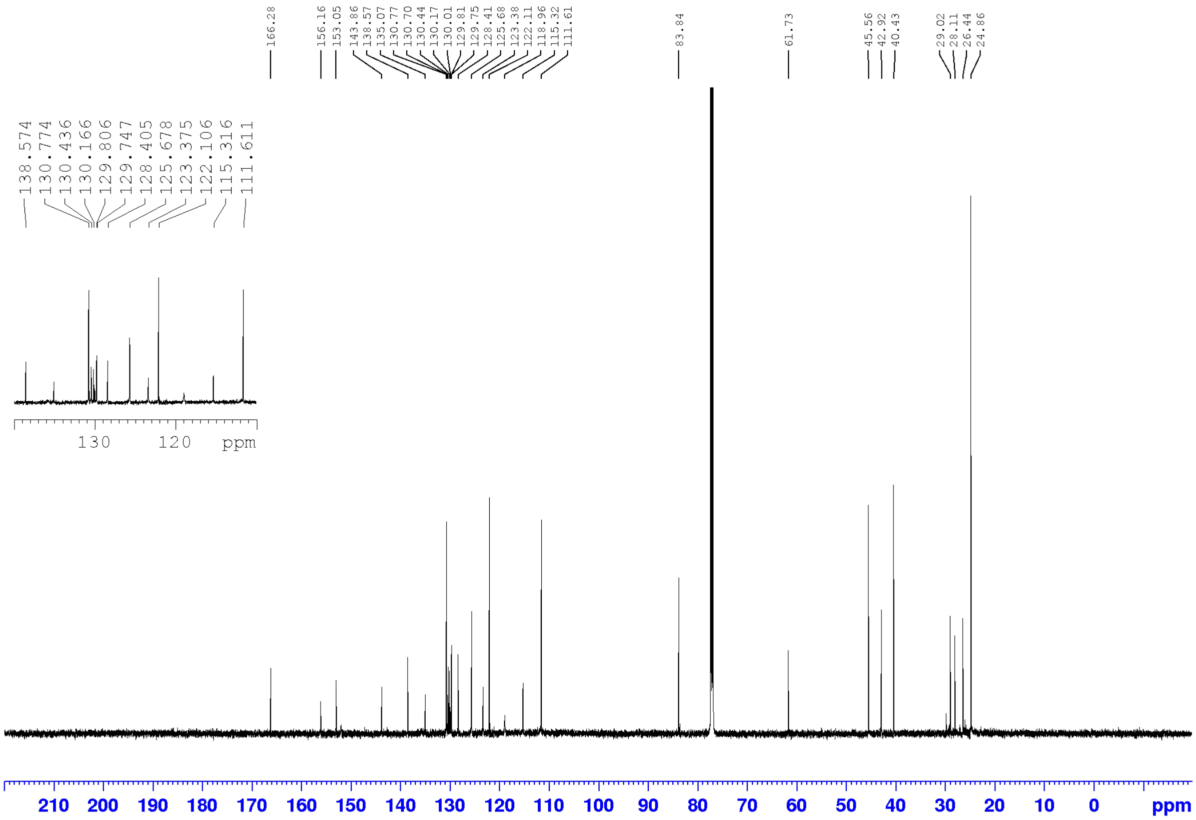
166.28
156.16
153.05
143.86
138.57
135.07
130.77
130.70
130.44
130.17
130.01
129.81
129.75
128.41
125.68
123.38
122.11
118.96
115.32
111.61
83.84
61.73
45.56
42.92
40.43
29.02
28.11
26.44
24.86
138.574
130.774
130.436
130.166
129.806
129.747
128.405
125.678
123.375
122.106
115.316
111.611
130
120
ppm
210
200
190
180
170
160
150
140
130
120
110
100
90
80
70
60
50
40
30
20
10
0
ppm

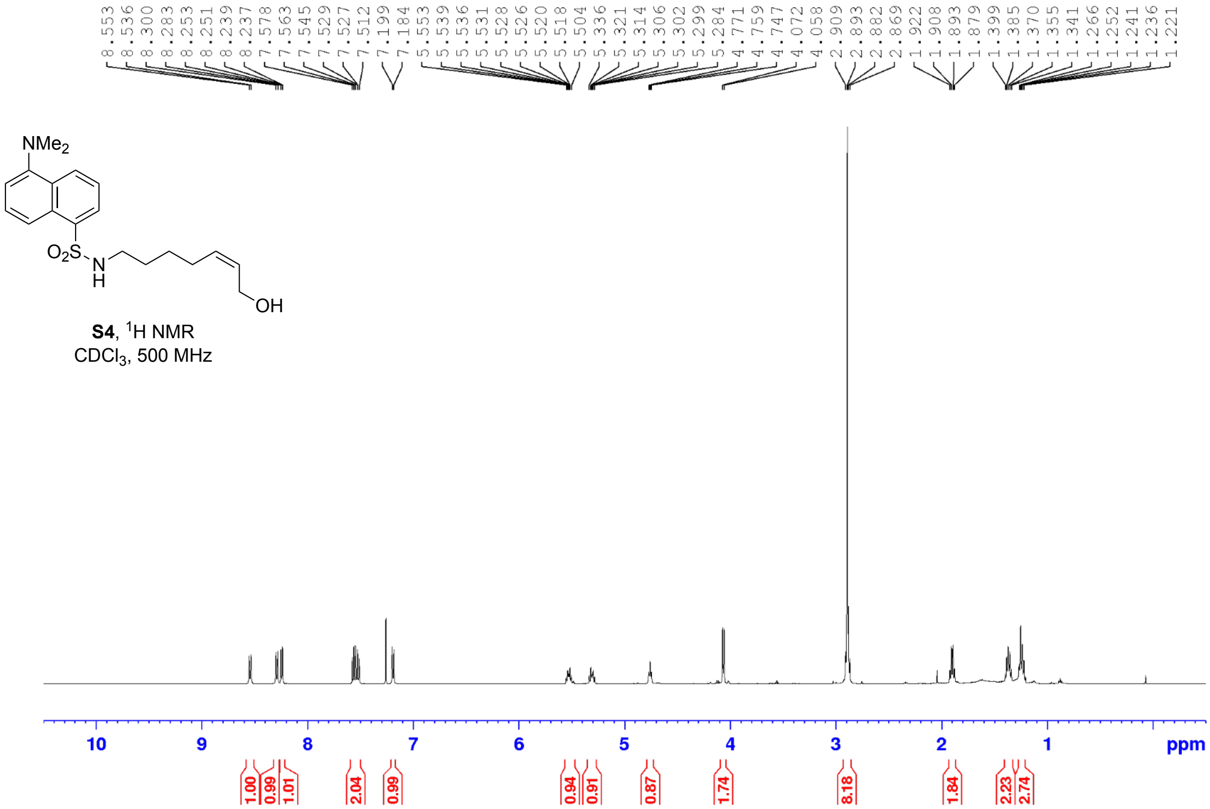

S4, ^{1}H NMR
$CDCl_3$, 500 MHz

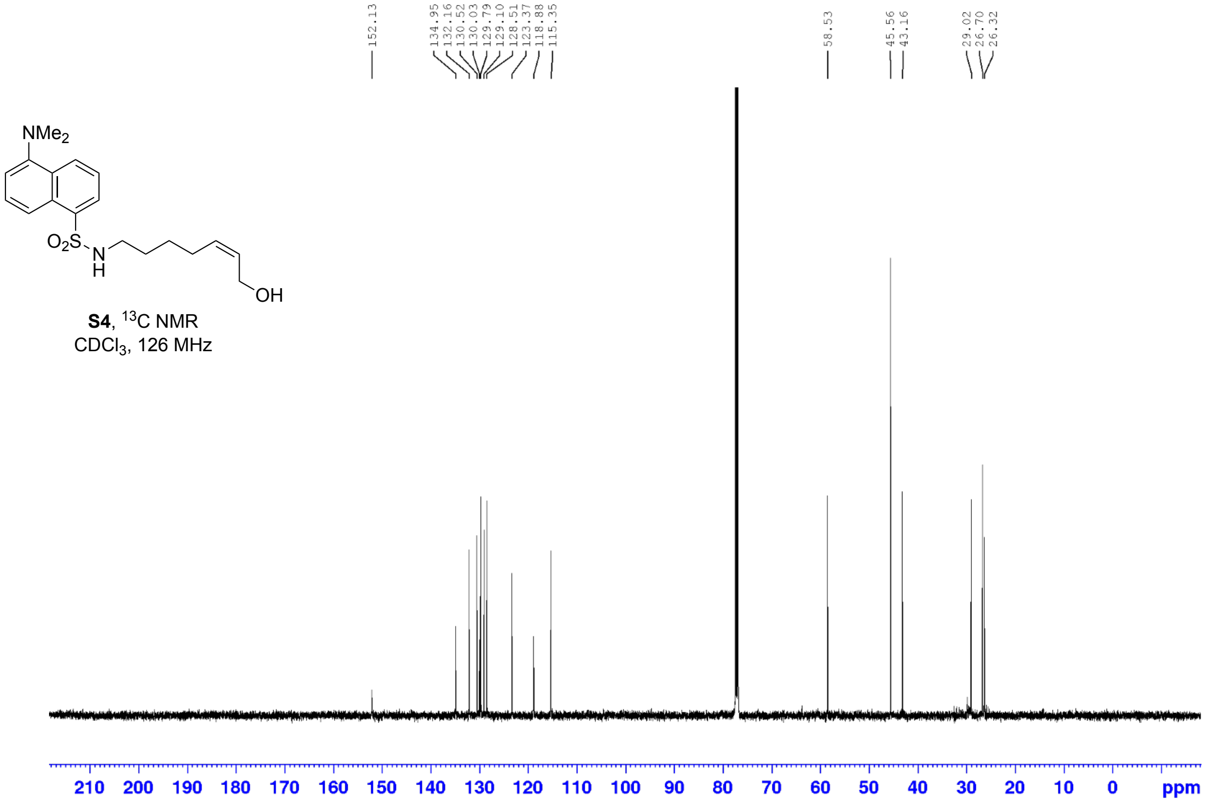

S4, ^{13}C NMR
$CDCl_3$, 126 MHz

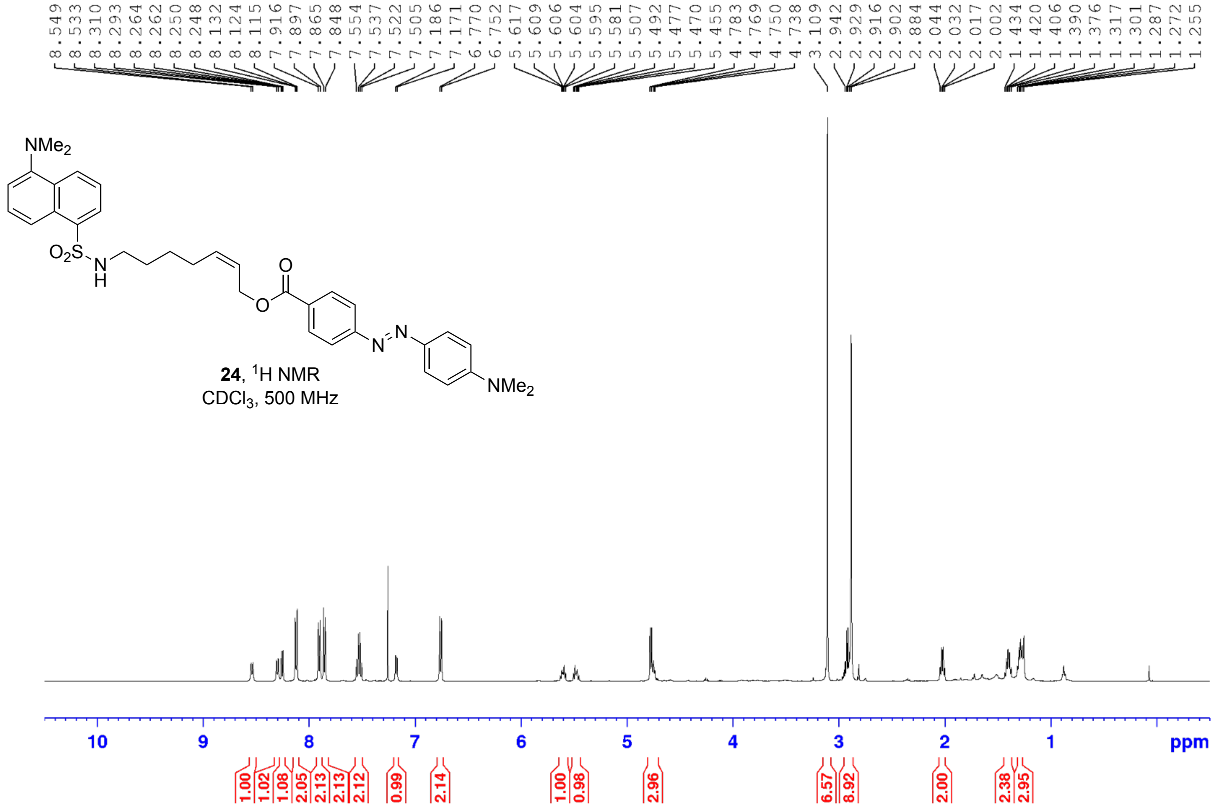

8.549
8.533
8.310
8.293
8.264
8.262
8.250
8.248
8.132
8.124
8.115
7.916
7.897
7.865
7.848
7.554
7.537
7.522
7.505
7.186
7.171
6.770
6.752
5.617
5.609
5.606
5.604
5.595
5.581
5.507
5.492
5.477
5.470
5.455
4.783
4.769
4.750
4.738
3.109
2.942
2.929
2.916
2.902
2.884
2.044
2.032
2.017
2.002
1.434
1.420
1.406
1.390
1.376
1.317
1.301
1.287
1.272
1.255
NMe2
O2S
N
H
O
O
N
N
NMe2
24, 1H NMR
CDCl3, 500 MHz
10
9
8
7
6
5
4
3
2
1
ppm
1.00
1.02
1.08
2.05
2.13
2.13
2.12
0.99
2.14
1.00
0.98
2.96
6.57
8.92
2.00
2.38
2.95

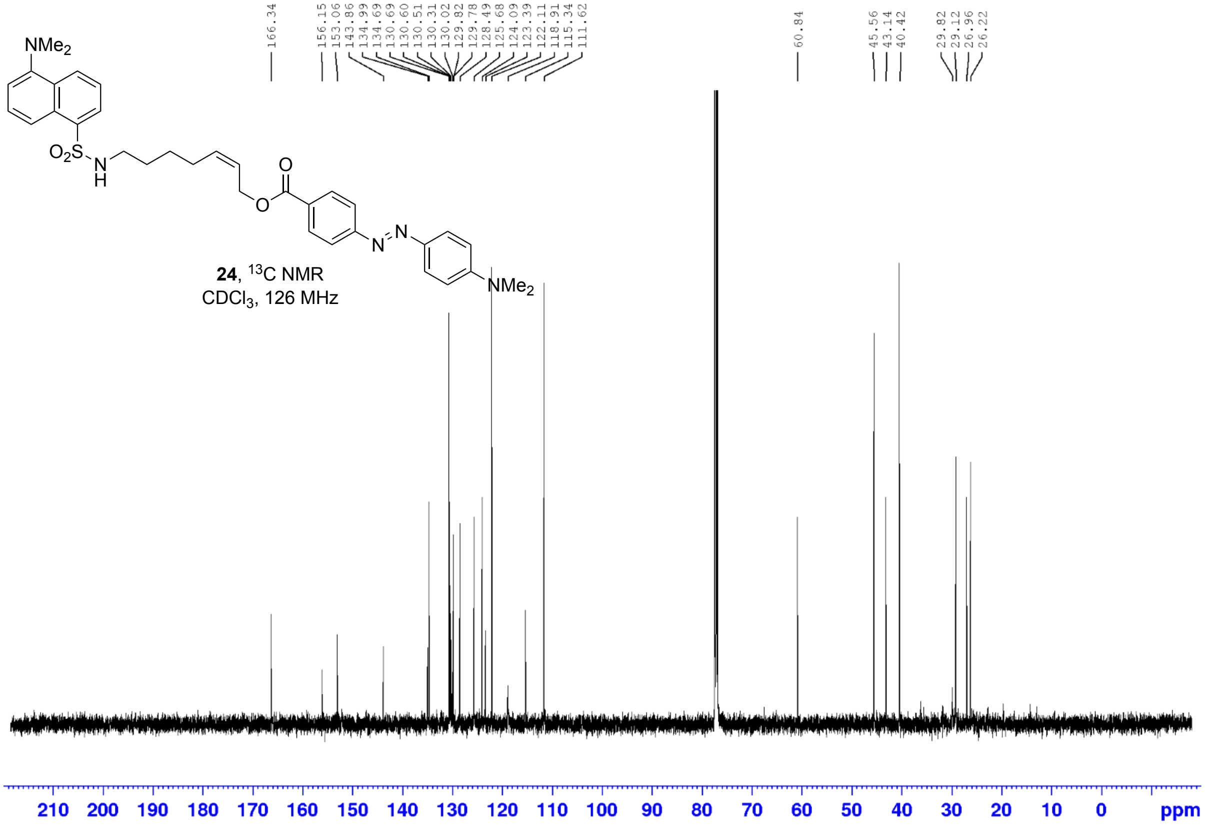

166.34
156.15
153.06
143.86
134.99
134.69
130.69
130.60
130.51
130.31
130.02
129.82
129.78
128.49
125.68
124.09
123.39
122.11
118.91
115.34
111.62
60.84
45.56
43.14
40.42
29.82
29.12
26.96
26.22
NMe2
O2S
N
H
O
O
N
N
NMe2
24, 13C NMR
CDCl3, 126 MHz
210
200
190
180
170
160
150
140
130
120
110
100
90
80
70
60
50
40
30
20
10
0
ppm

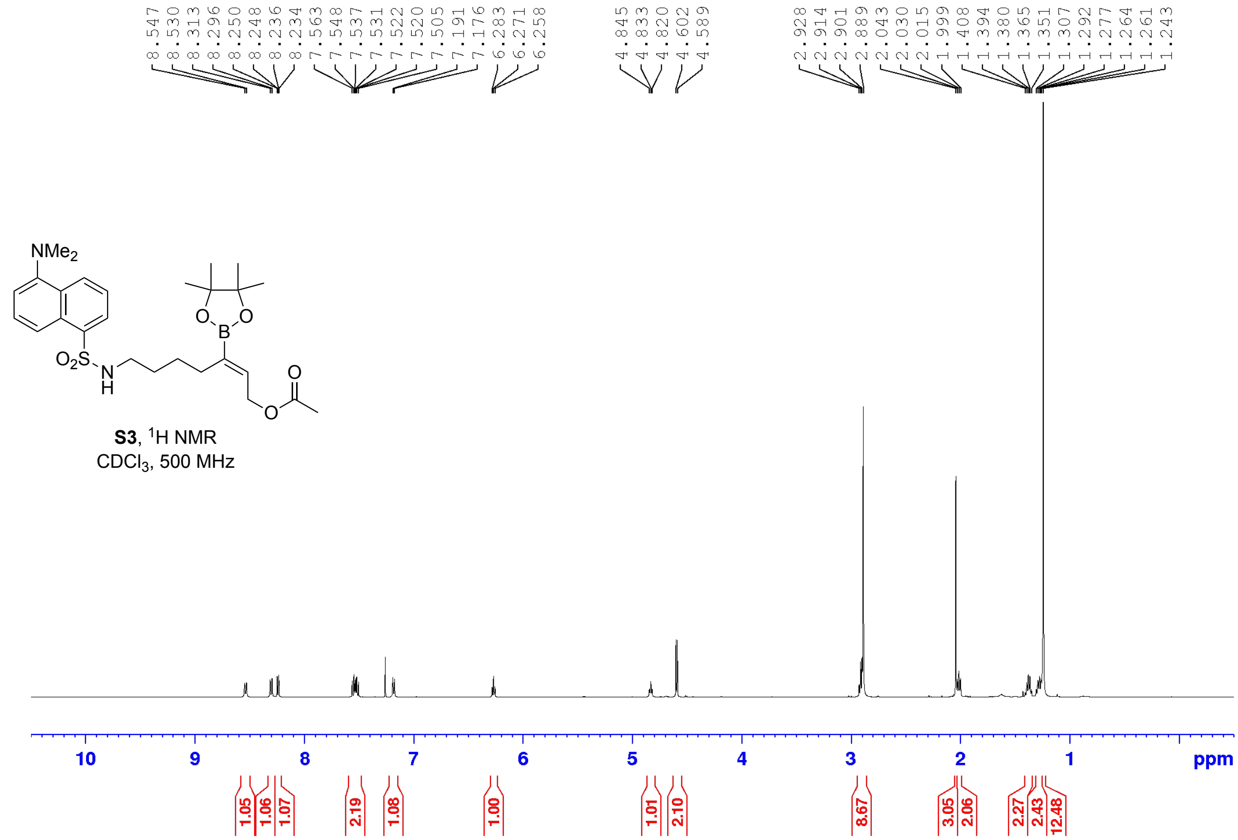

S3, ^{1}H NMR
$CDCl_3$, 500 MHz

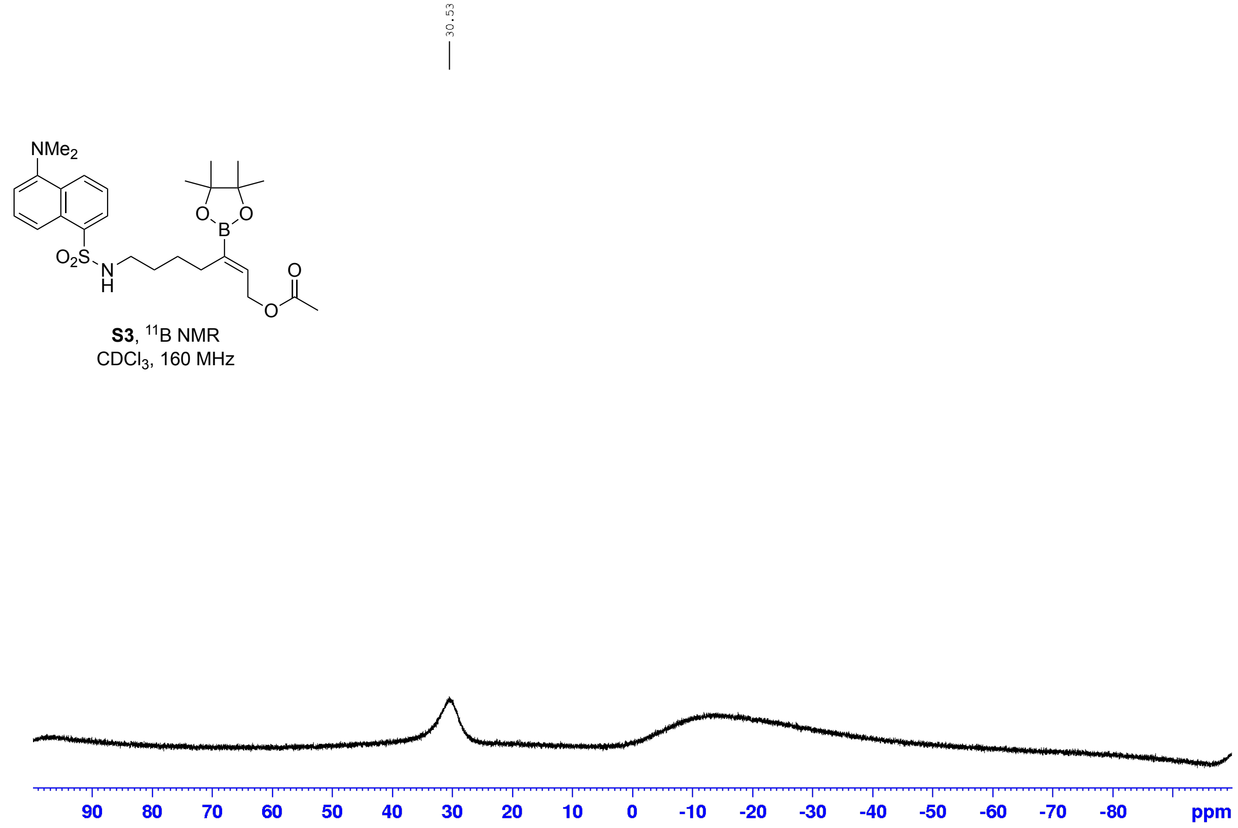

S3, ^{11}B NMR
$CDCl_3$, 160 MHz

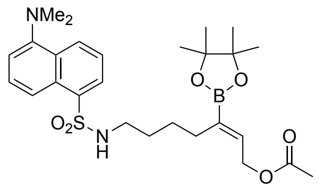

S3, ^{13}C NMR
$CDCl_3$, 126 MHz

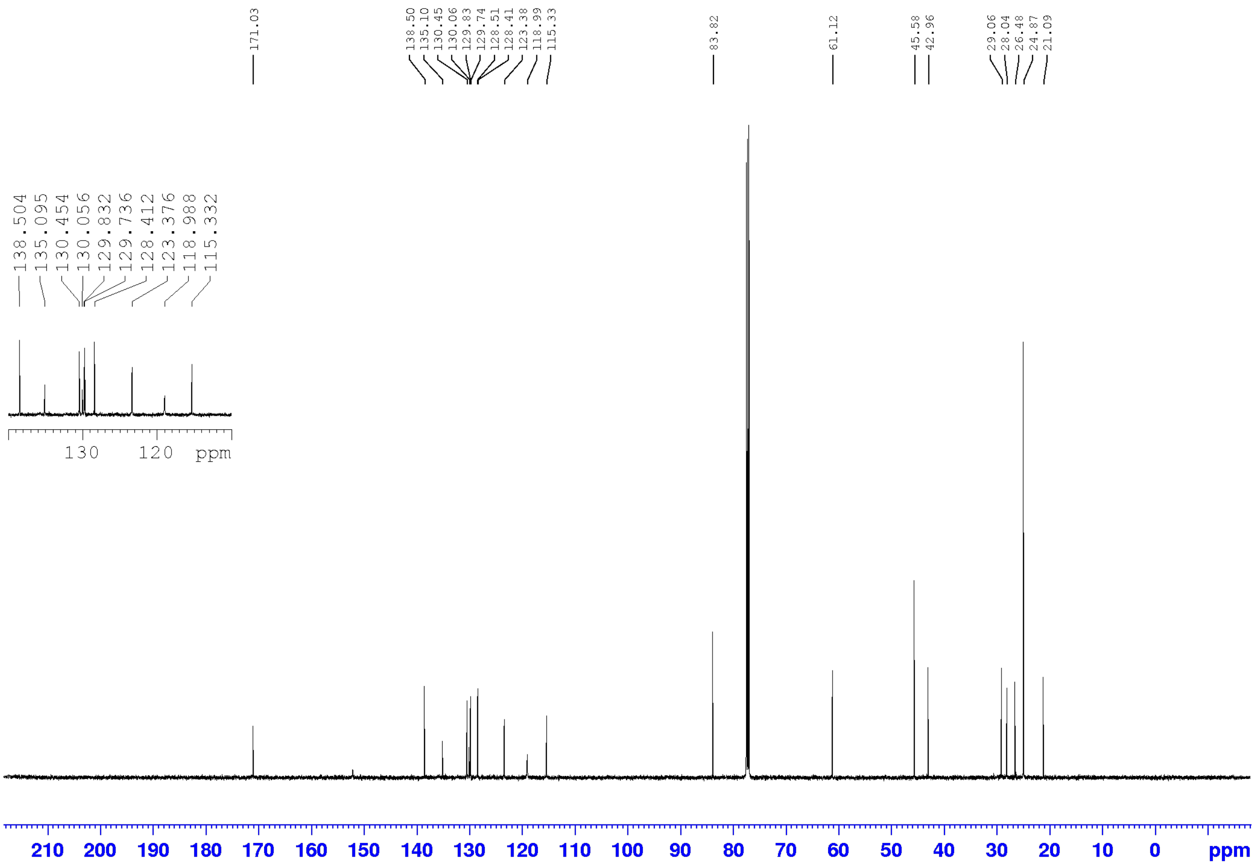

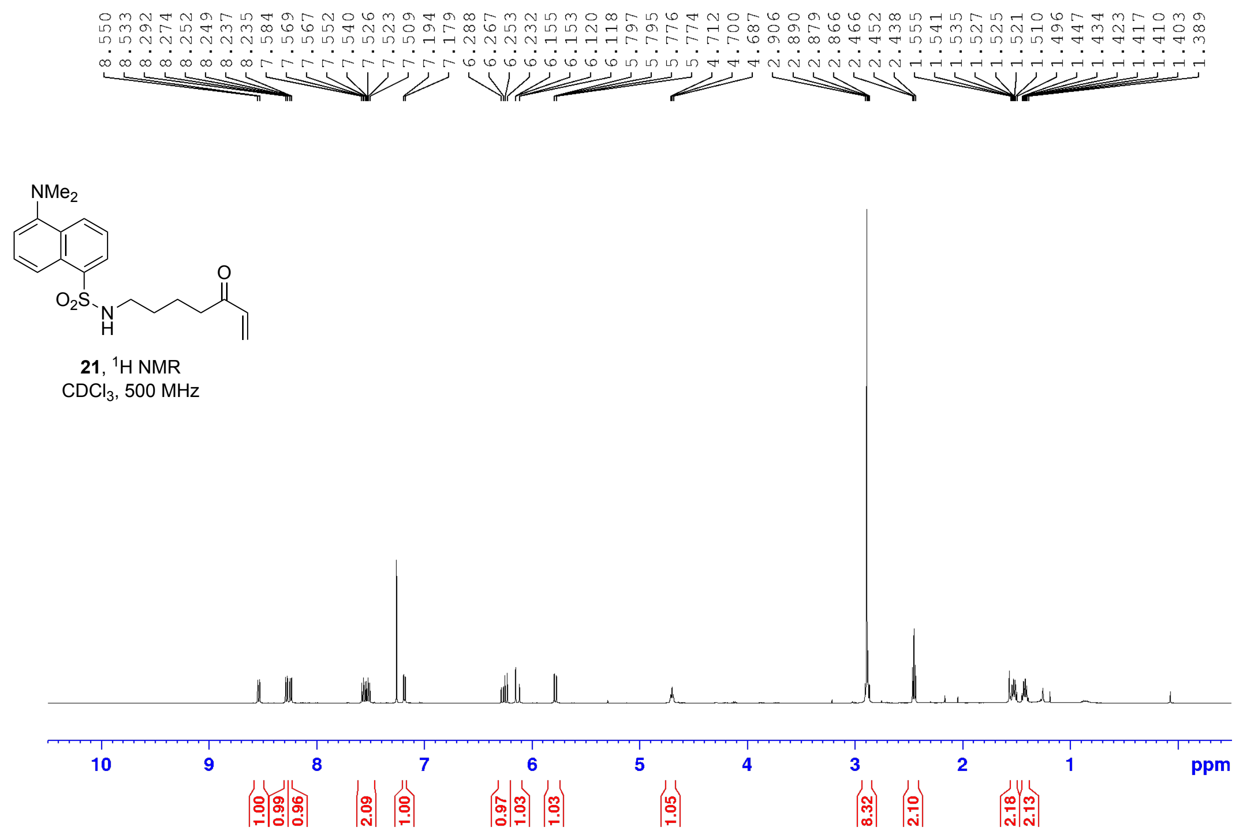
8.550 8.533 8.292 8.274 8.252 8.249 8.237 8.235 7.584 7.569 7.567 7.552 7.540 7.526 7.523 7.509 7.194 7.179 6.288 6.267 6.253 6.232 6.155 6.153 6.120 6.118 5.797 5.795 5.776 5.774 4.712 4.700 4.687 2.906 2.890 2.879 2.866 2.466 2.452 2.438 1.555 1.541 1.535 1.527 1.525 1.521 1.510 1.496 1.447 1.434 1.423 1.417 1.410 1.403 1.389
NMe2
O
O2S
N
H
21, 1H NMR
CDCl3, 500 MHz
10 9 8 7 6 5 4 3 2 1 ppm
1.00 0.99 0.96 2.09 1.00 0.97 1.03 1.03 1.05 8.32 2.10 2.18 2.13

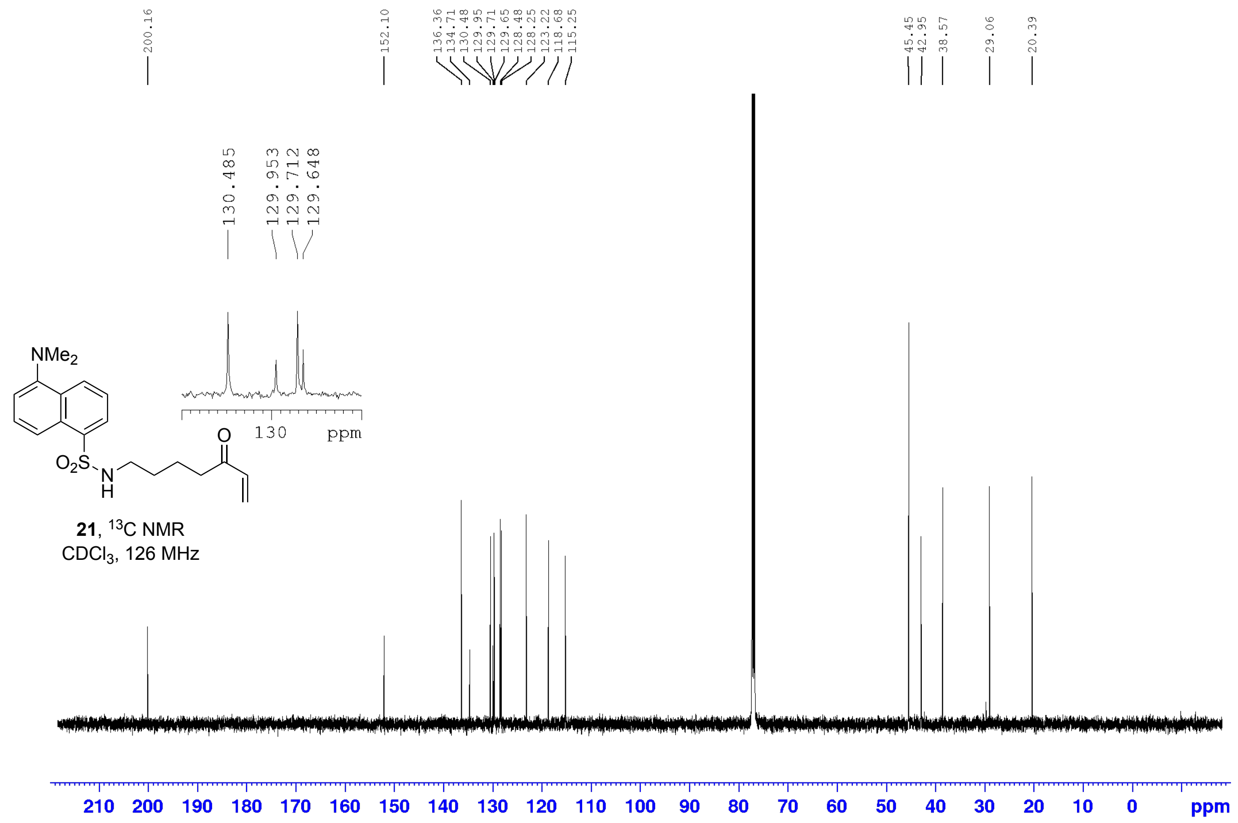
200.16 152.10 136.36 134.71 130.48 129.95 129.71 129.65 128.48 128.25 123.22 118.68 115.25 45.45 42.95 38.57 29.06 20.39
130.485 129.953 129.712 129.648
130 ppm
NMe2
O
O2S
N
H
21, 13C NMR
CDCl3, 126 MHz
210 200 190 180 170 160 150 140 130 120 110 100 90 80 70 60 50 40 30 20 10 0 ppm

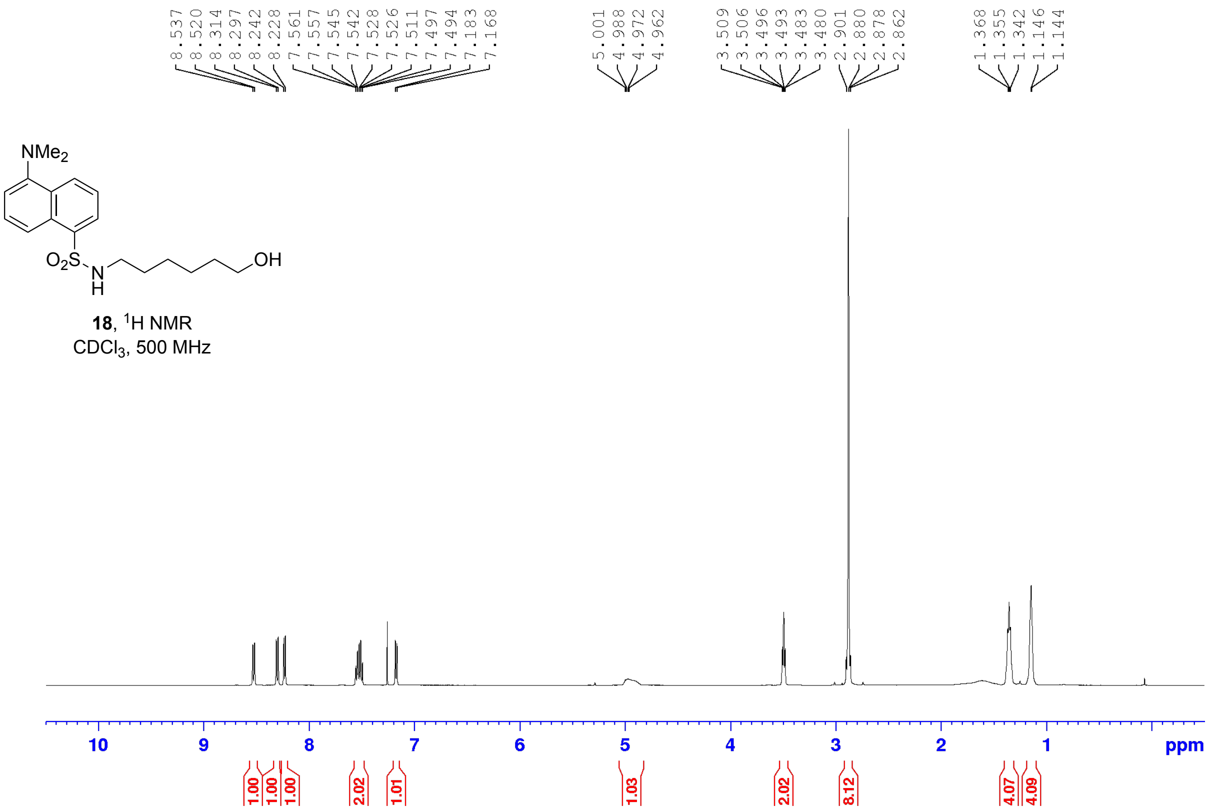
8.537
8.520
8.314
8.297
8.242
8.228
7.561
7.557
7.545
7.542
7.528
7.526
7.511
7.497
7.494
7.183
7.168
5.001
4.988
4.972
4.962
3.509
3.506
3.496
3.493
3.483
3.480
2.901
2.880
2.878
2.862
1.368
1.355
1.342
1.146
1.144
NMe2
O2S
N
H
OH
18, 1H NMR
CDCl3, 500 MHz
10
9
8
7
6
5
4
3
2
1
ppm
1.00
1.00
1.00
2.02
1.01
1.03
2.02
8.12
4.07
4.09

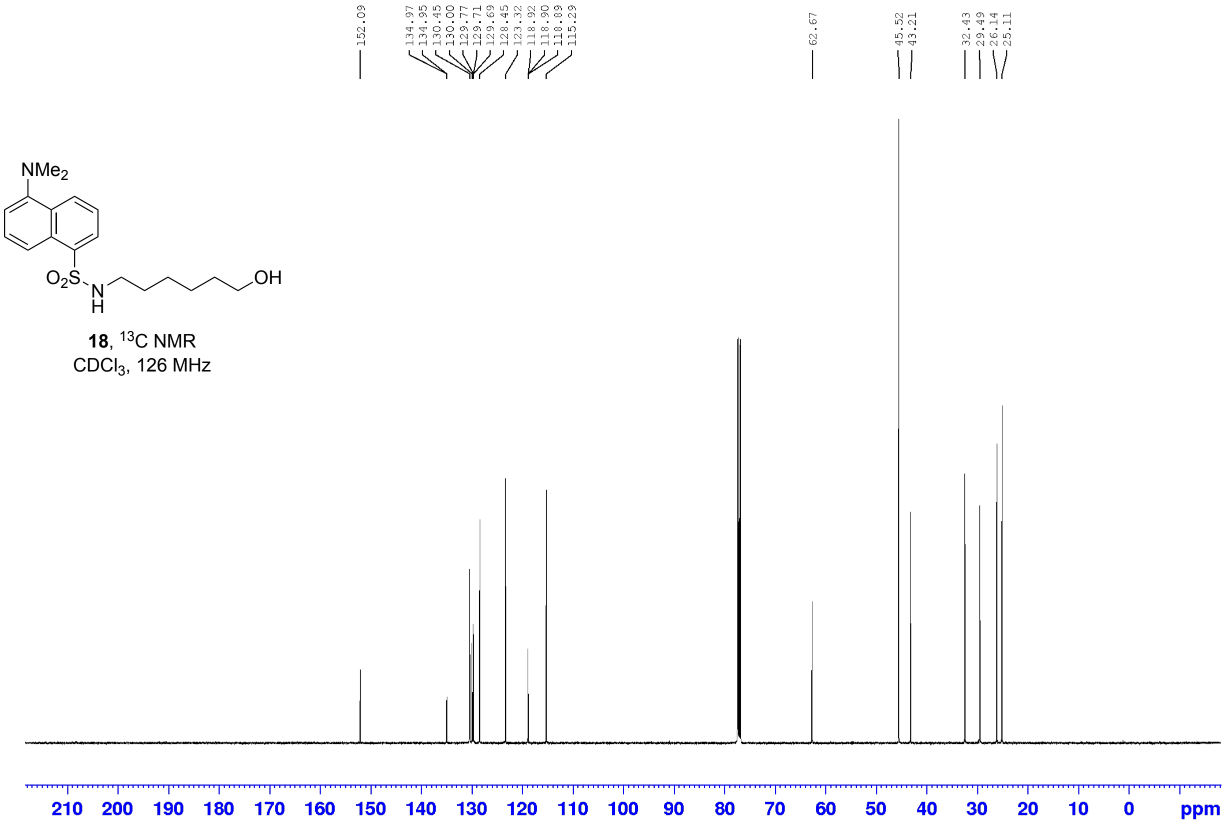
152.09
134.97
134.95
130.45
130.00
129.77
129.71
129.69
128.45
123.32
118.92
118.90
118.89
115.29
62.67
45.52
43.21
32.43
29.49
26.14
25.11
NMe2
O2S
N
H
OH
18, 13C NMR
CDCl3, 126 MHz
210
200
190
180
170
160
150
140
130
120
110
100
90
80
70
60
50
40
30
20
10
0
ppm

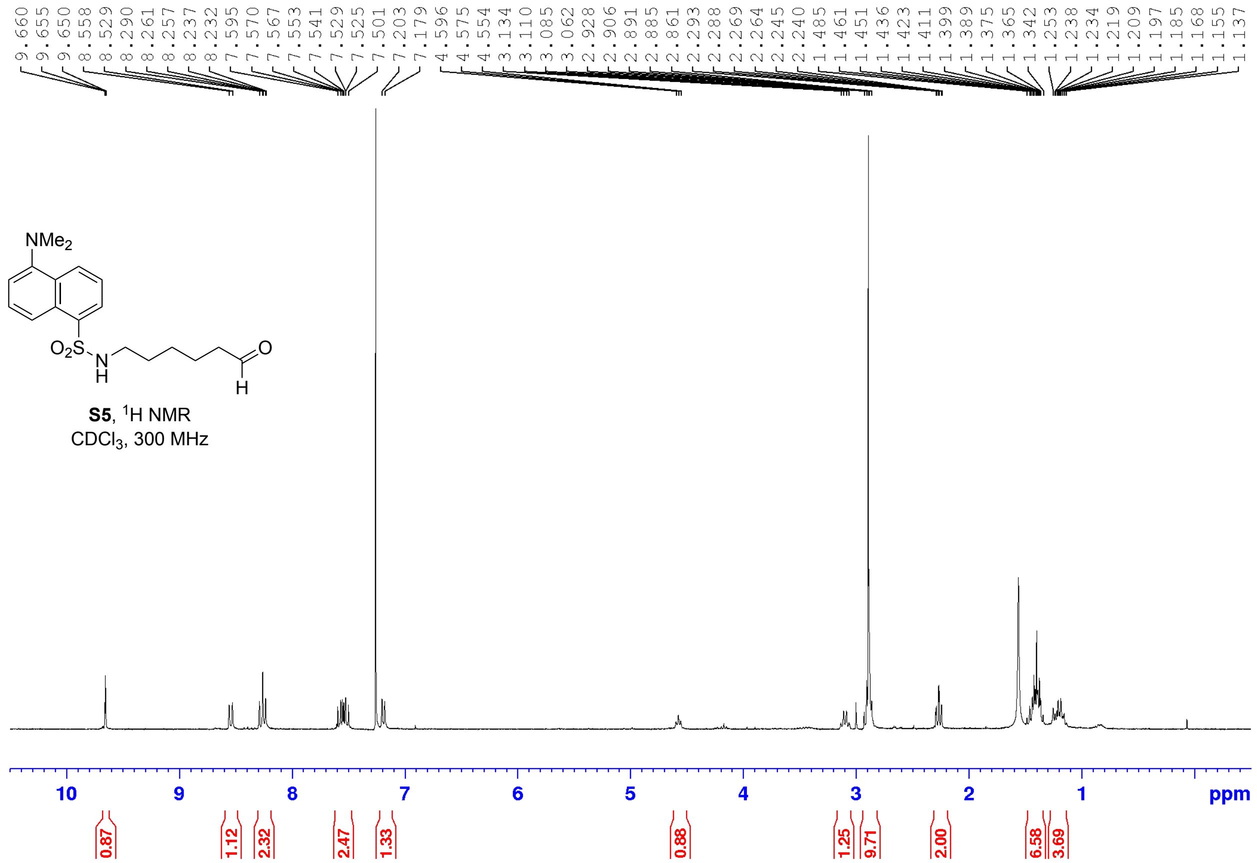

NMe2
O2S
N
H
O
H
S5, 1H NMR
CDCl3, 300 MHz
ppm

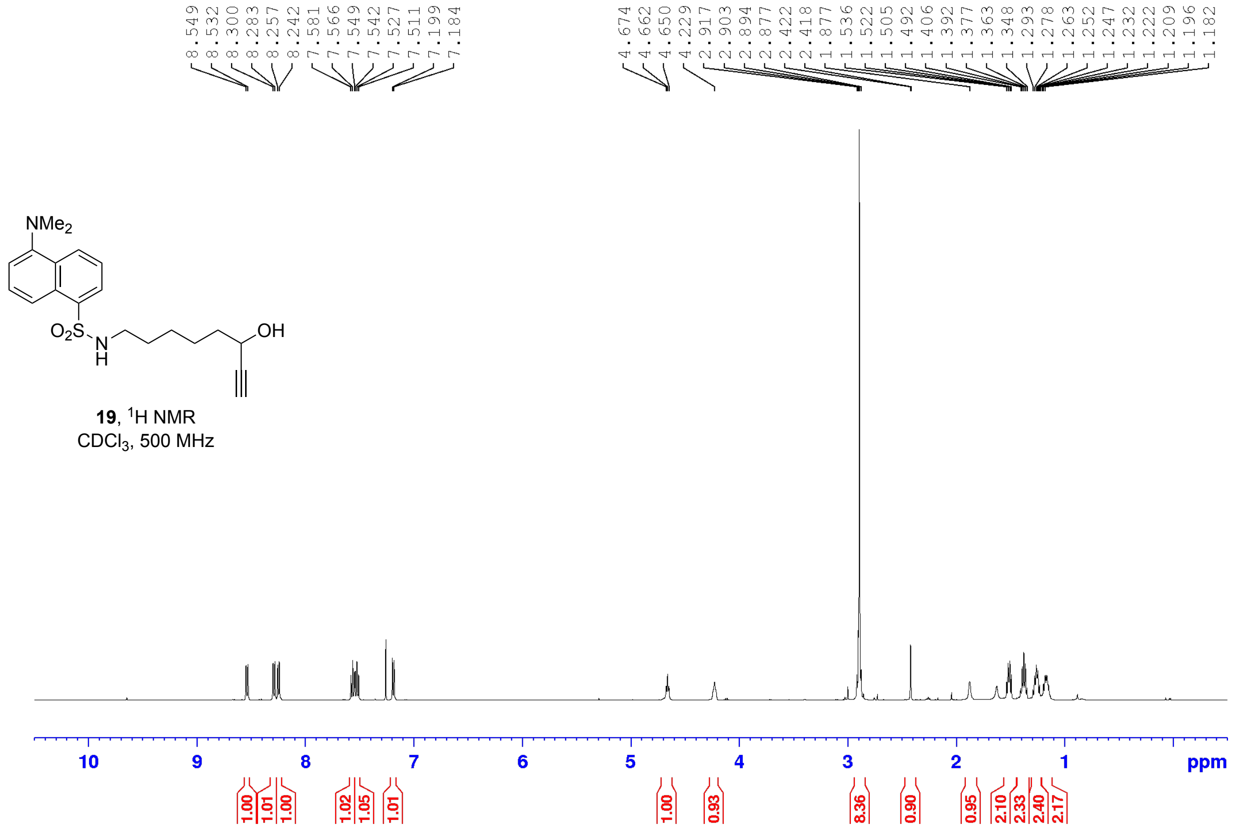
NMe2
O2S
N
H
OH
19, 1H NMR
CDCl3, 500 MHz
8.549 8.532 8.300 8.283 8.257 8.242 7.581 7.566 7.549 7.542 7.527 7.511 7.199 7.184
4.674 4.662 4.650 4.229 2.917 2.903 2.894 2.877 2.422 2.418 1.877 1.536 1.522 1.505 1.492 1.406 1.392 1.377 1.363 1.348 1.293 1.278 1.263 1.252 1.247 1.232 1.222 1.209 1.196 1.182
1.00 1.01 1.00 1.02 1.05 1.01 1.00 0.93 8.36 0.90 0.95 2.10 2.33 2.40 2.17
10 9 8 7 6 5 4 3 2 1 ppm

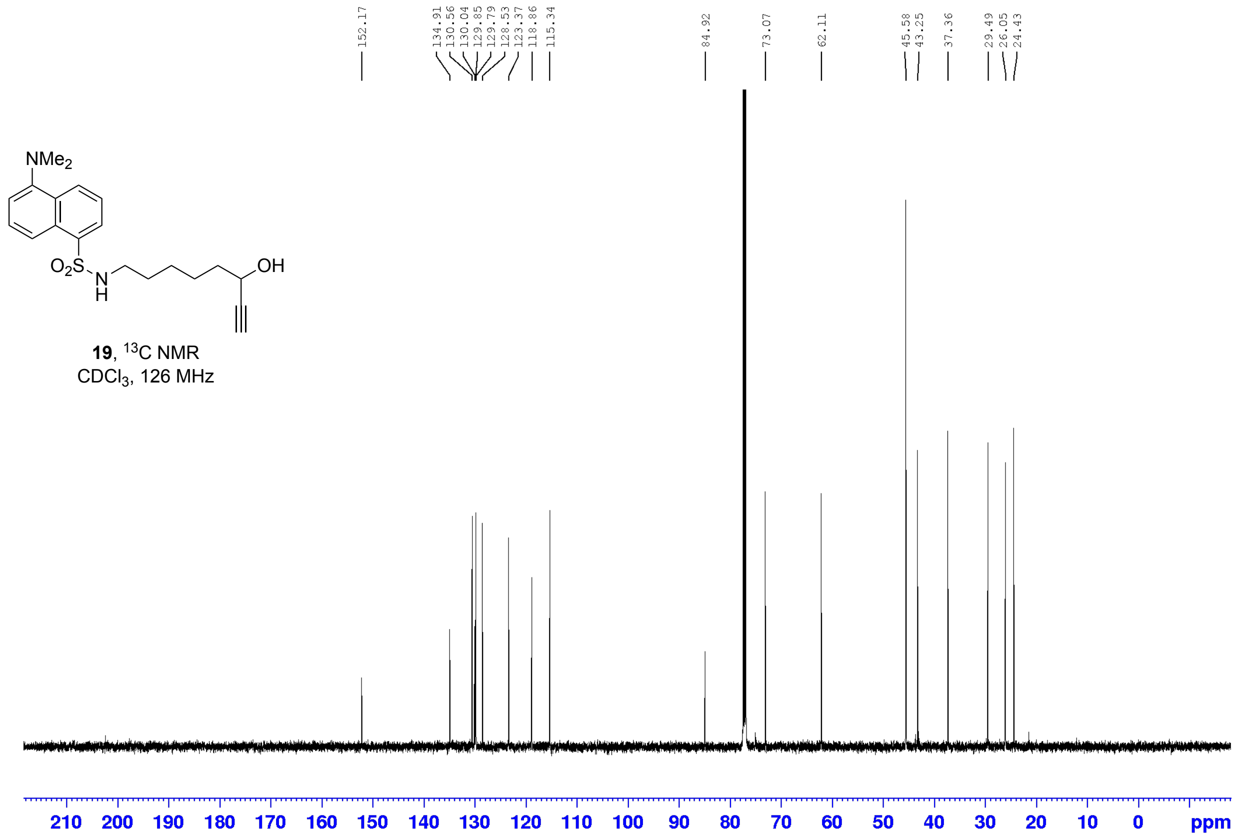
NMe2
O2S
N
H
OH
19, 13C NMR
CDCl3, 126 MHz
152.17
134.91 130.56 130.04 129.85 129.79 128.53 123.37 118.86 115.34
84.92
73.07
62.11
45.58 43.25
37.36
29.49 26.05 24.43
210 200 190 180 170 160 150 140 130 120 110 100 90 80 70 60 50 40 30 20 10 0 ppm

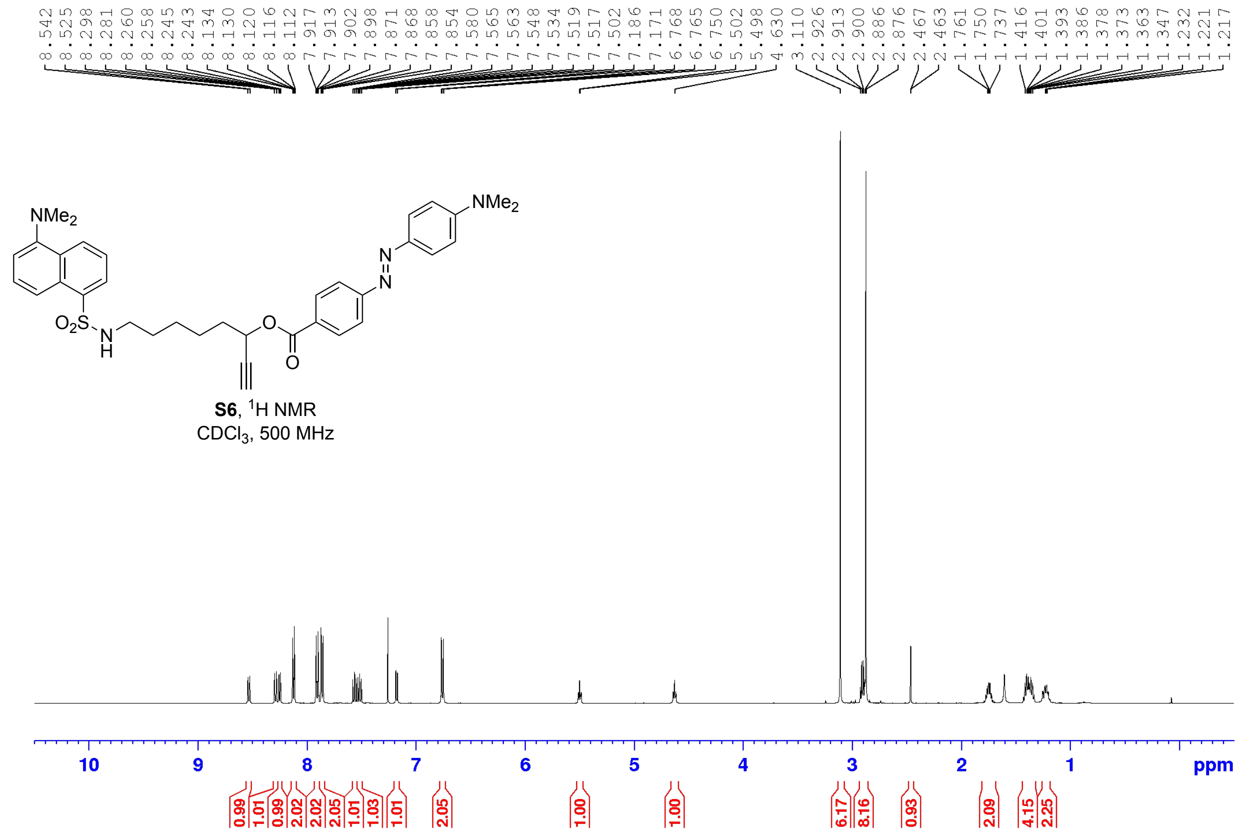
NMe2
N=N
O2S
N
H
O
O
NMe2
S6, 1H NMR
CDCl3, 500 MHz
8.542 8.525 8.298 8.281 8.260 8.258 8.245 8.243 8.134 8.130 8.120 8.116 8.112 7.917 7.913 7.902 7.898 7.871 7.868 7.858 7.854 7.580 7.565 7.563 7.548 7.534 7.519 7.517 7.502 7.186 7.171 6.768 6.765 6.750 5.502 5.498 4.630 3.110 2.926 2.913 2.900 2.886 2.876 2.467 2.463 1.761 1.750 1.737 1.416 1.401 1.393 1.386 1.378 1.373 1.363 1.347 1.232 1.221 1.217
0.99 1.01 0.99 2.02 2.02 2.05 1.01 1.03 1.01 2.05 1.00 1.00 6.17 8.16 0.93 2.09 4.15 2.25
10 9 8 7 6 5 4 3 2 1 ppm

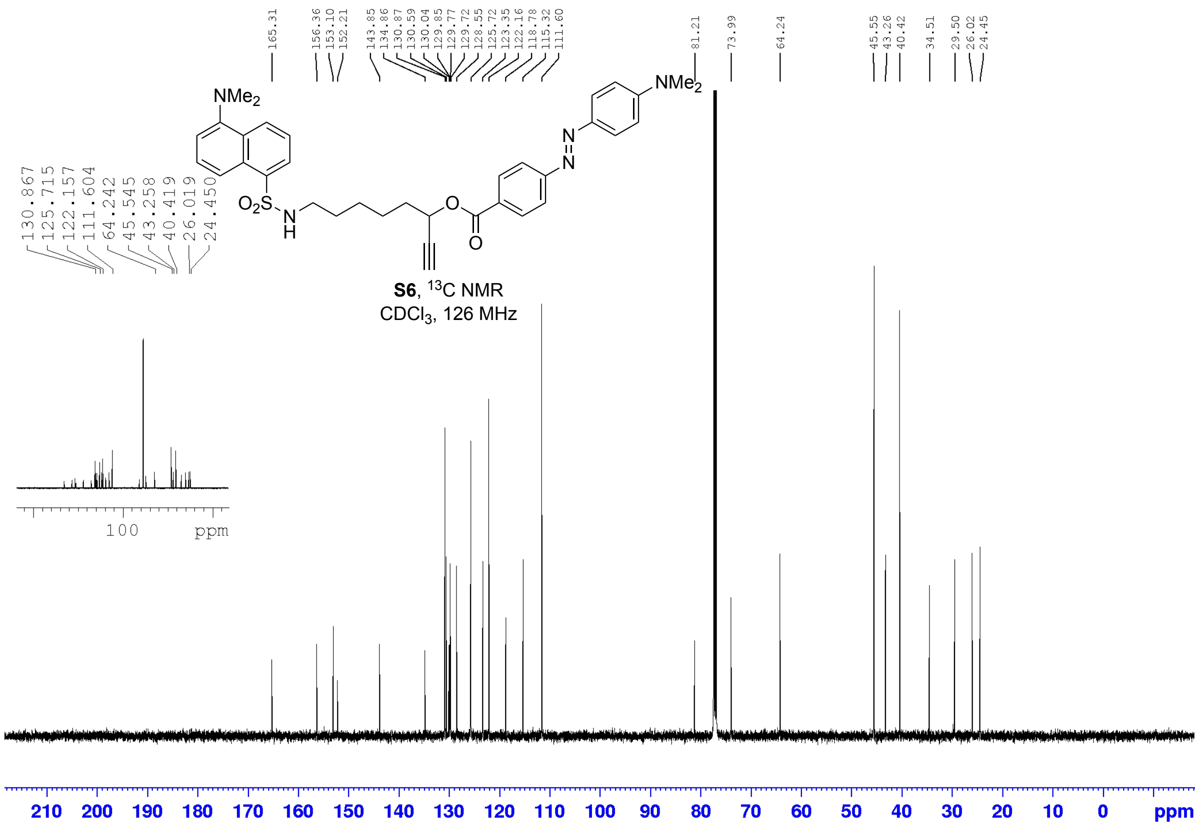
165.31 156.36 153.10 152.21 143.85 134.86 130.87 130.59 130.04 129.85 129.77 129.72 128.55 125.72 123.35 122.16 118.78 115.32 111.60 81.21 73.99 64.24 45.55 43.26 40.42 34.51 29.50 26.02 24.45
130.867 125.715 122.157 111.604 64.242 45.545 43.258 40.419 26.019 24.450
100 ppm
NMe2
N=N
O2S
N
H
O
O
NMe2
S6, 13C NMR
CDCl3, 126 MHz
210 200 190 180 170 160 150 140 130 120 110 100 90 80 70 60 50 40 30 20 10 0 ppm

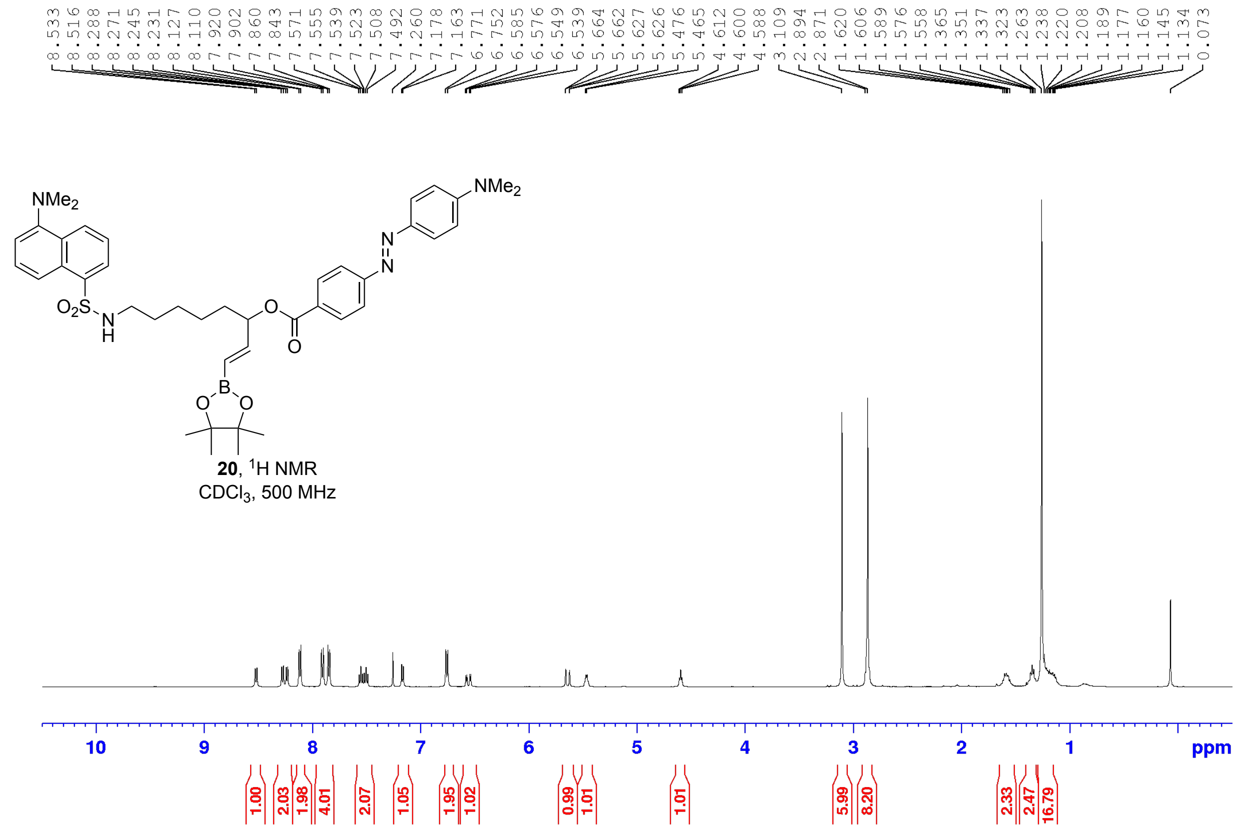

20, ^{1}H NMR
$CDCl_3$, 500 MHz

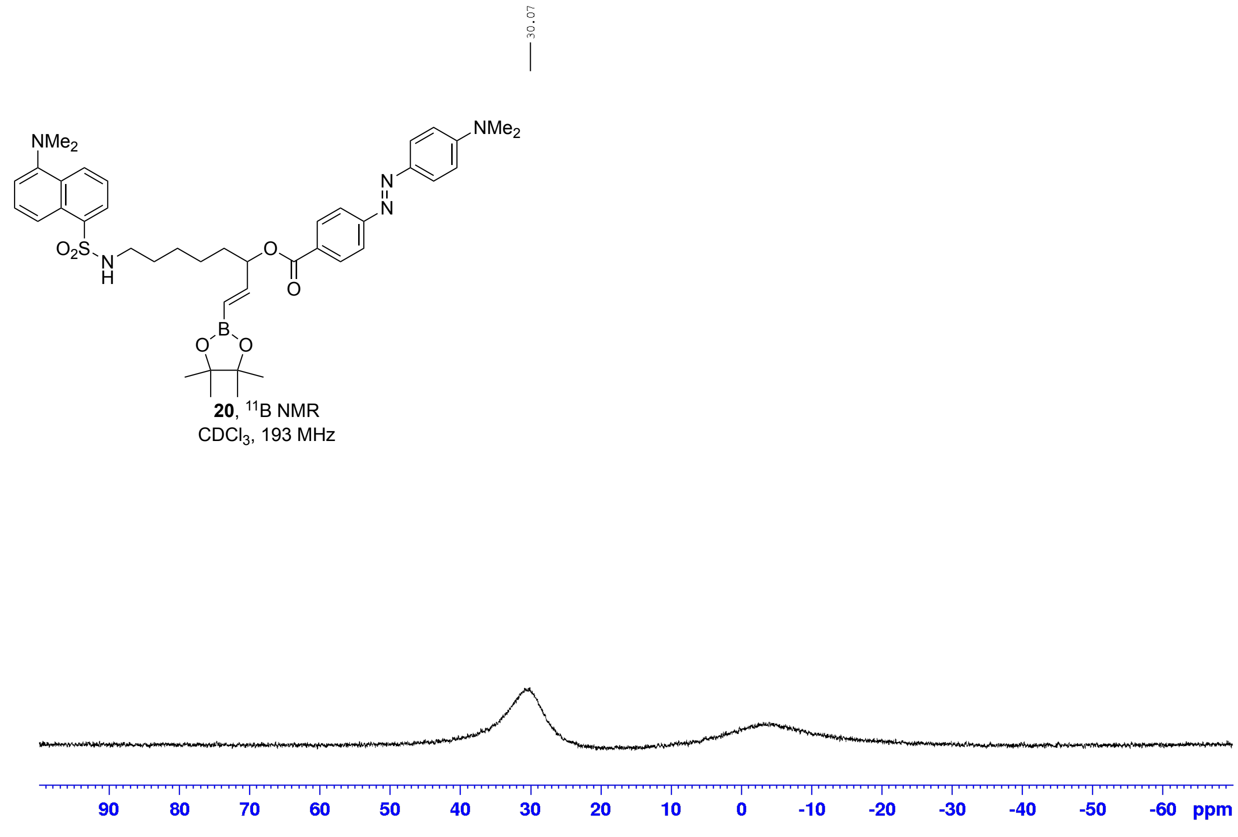

20, ^{11}B NMR
$CDCl_3$, 193 MHz

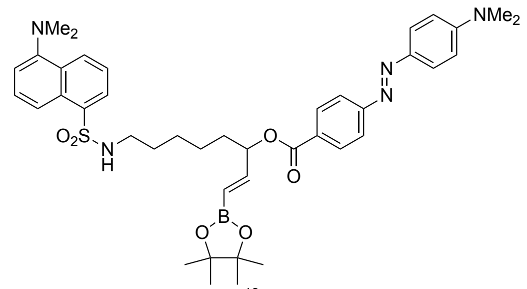

20, ^{13}C NMR
$CDCl_3$, 126 MHz

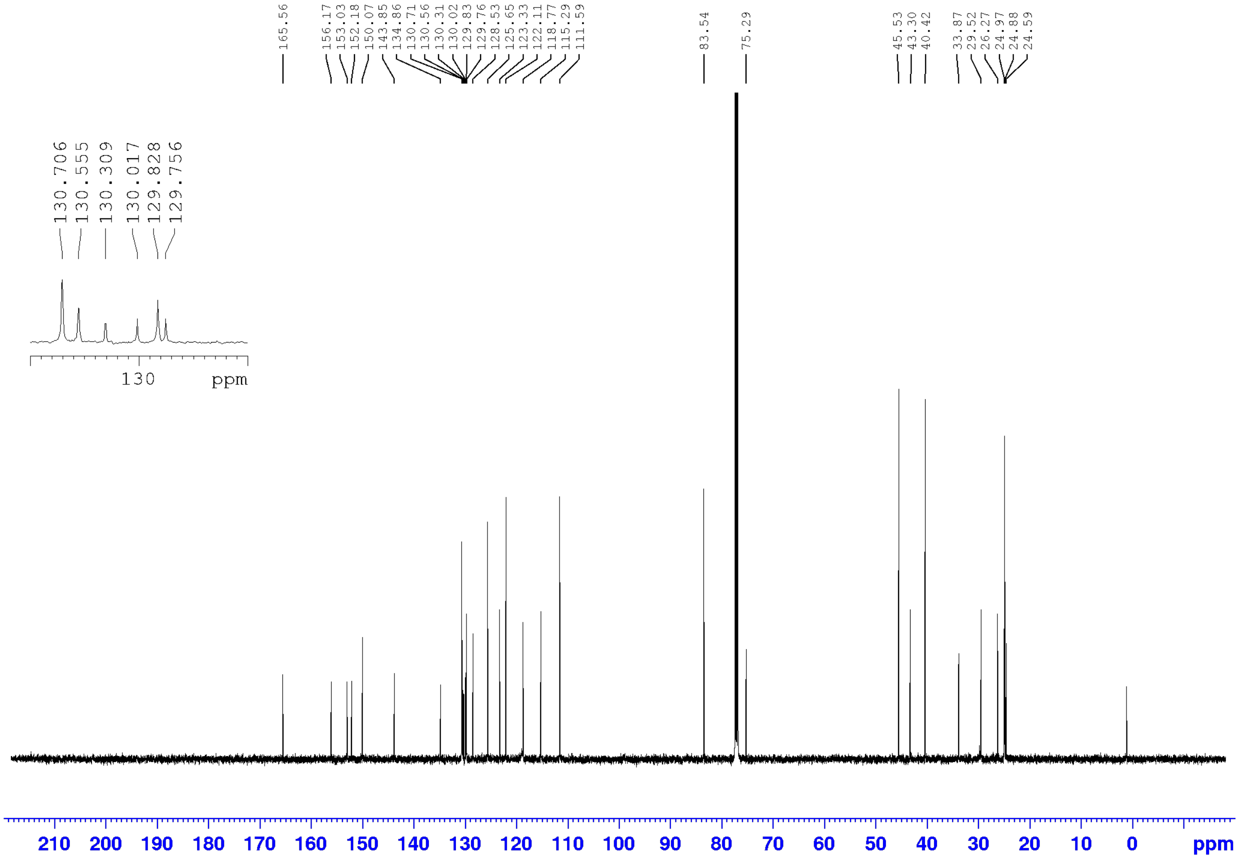

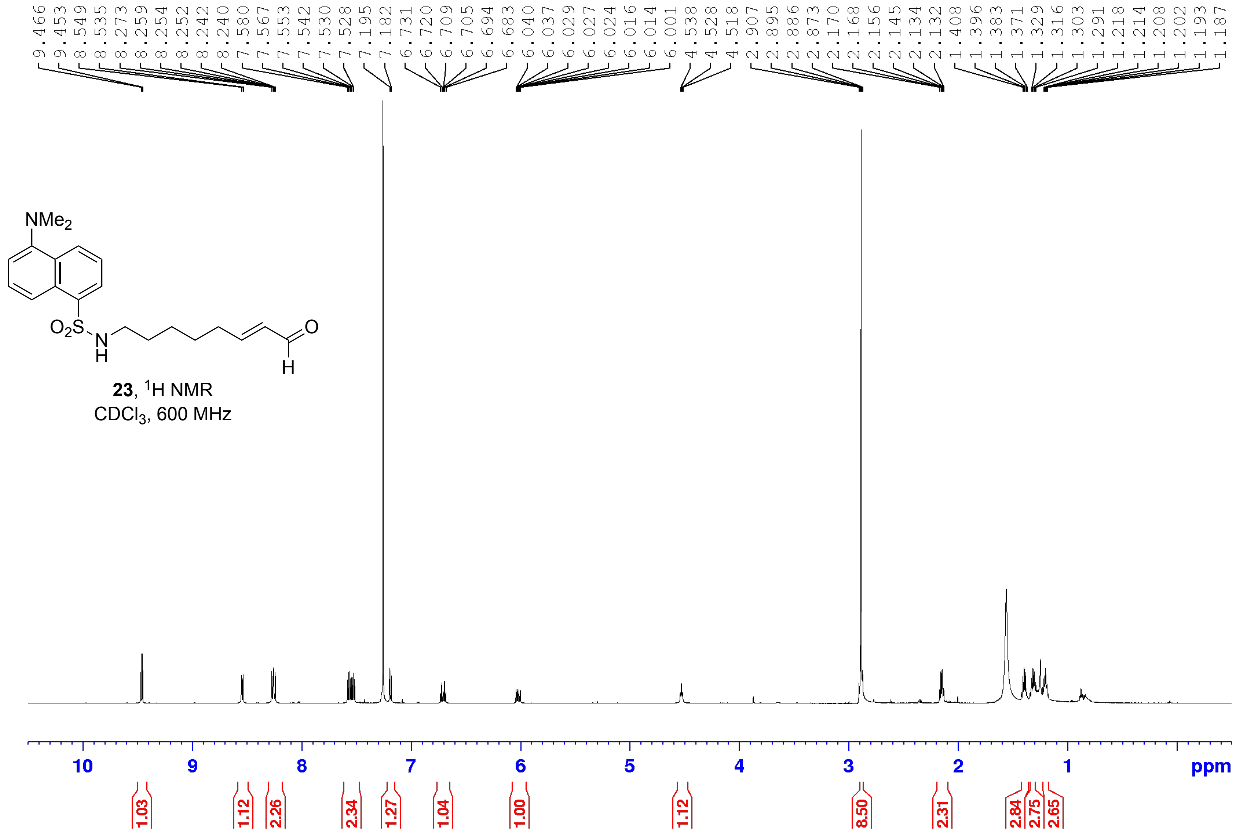
9.466 9.453 8.549 8.535 8.273 8.259 8.254 8.252 8.242 8.240 7.580 7.567 7.553 7.542 7.530 7.528 7.195 7.182 6.731 6.720 6.709 6.705 6.694 6.683 6.040 6.037 6.029 6.027 6.024 6.016 6.014 6.001 4.538 4.528 4.518 2.907 2.895 2.886 2.873 2.870 2.168 2.156 2.145 2.134 2.132 1.408 1.396 1.383 1.371 1.329 1.316 1.303 1.291 1.218 1.214 1.208 1.202 1.193 1.187
NMe2
O2S
N
H
O
H
23, 1H NMR
CDCl3, 600 MHz
10 9 8 7 6 5 4 3 2 1 ppm
1.03 1.12 2.26 2.34 1.27 1.04 1.00 1.12 8.50 2.31 2.84 2.75 2.65

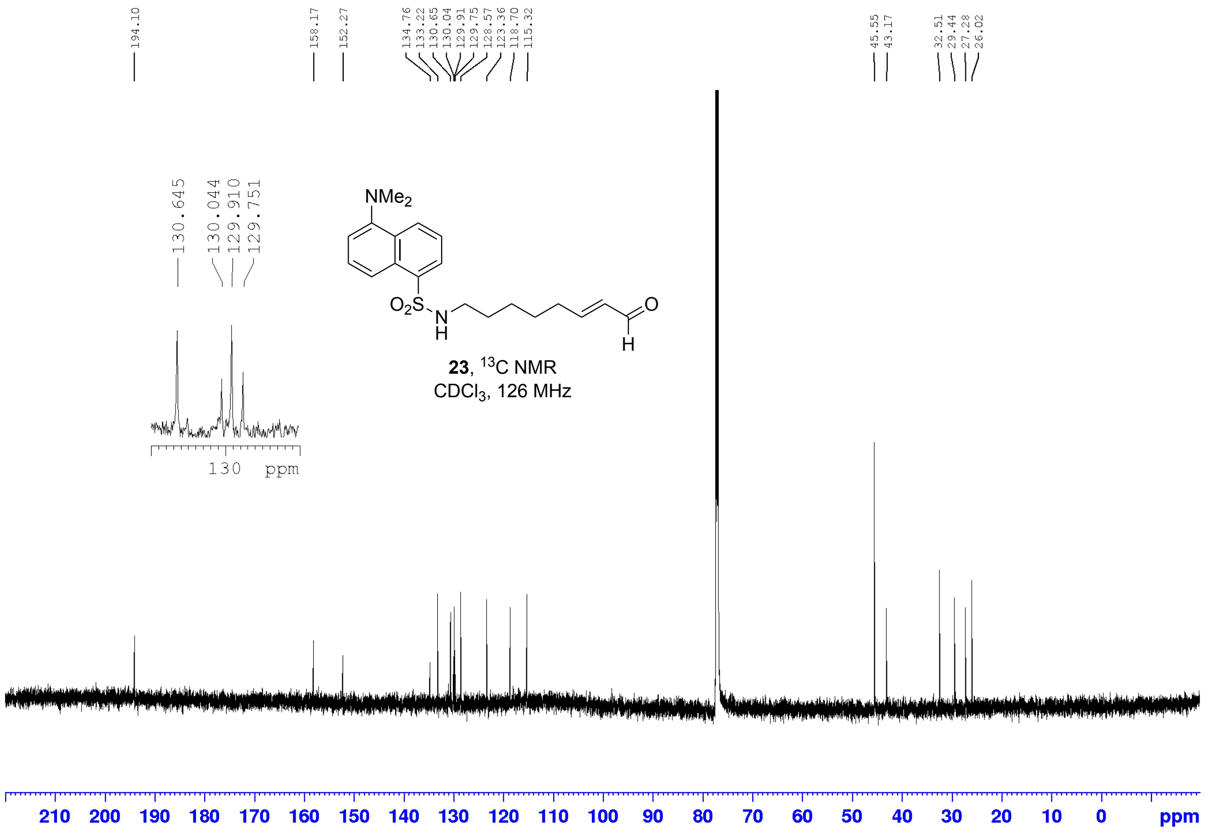
194.10 158.17 152.27 134.76 133.22 130.65 130.04 129.91 129.75 128.57 123.36 118.70 115.32 45.55 43.17 32.51 29.44 27.28 26.02
130.645 130.044 129.910 129.751
130 ppm
NMe2
O2S
N
H
O
H
23, 13C NMR
CDCl3, 126 MHz
210 200 190 180 170 160 150 140 130 120 110 100 90 80 70 60 50 40 30 20 10 0 ppm

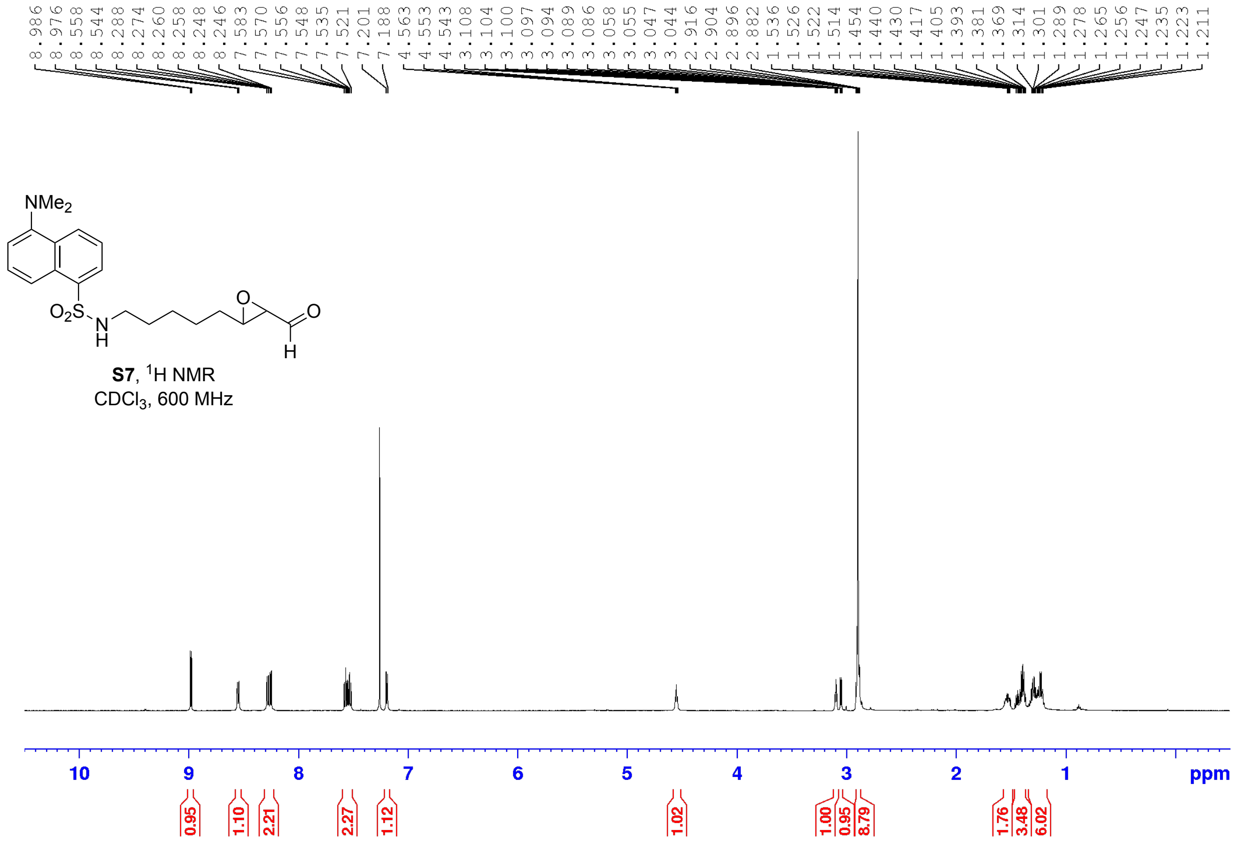
8.986 8.976 8.558 8.544 8.288 8.274 8.260 8.258 8.248 8.246 7.583 7.570 7.556 7.548 7.535 7.521 7.201 7.188 4.563 4.553 4.543 3.108 3.100 3.097 3.094 3.089 3.086 3.058 3.055 3.047 3.044 2.916 2.904 2.896 2.882 1.536 1.526 1.522 1.514 1.454 1.440 1.430 1.417 1.405 1.393 1.381 1.369 1.314 1.301 1.289 1.278 1.265 1.256 1.247 1.235 1.223 1.211
NMe2
O2S
N
H
O
O
H
S7, 1H NMR
CDCl3, 600 MHz
10 9 8 7 6 5 4 3 2 1 ppm
0.95 1.10 2.21 2.27 1.12 1.02 1.00 0.95 8.79 1.76 3.48 6.02

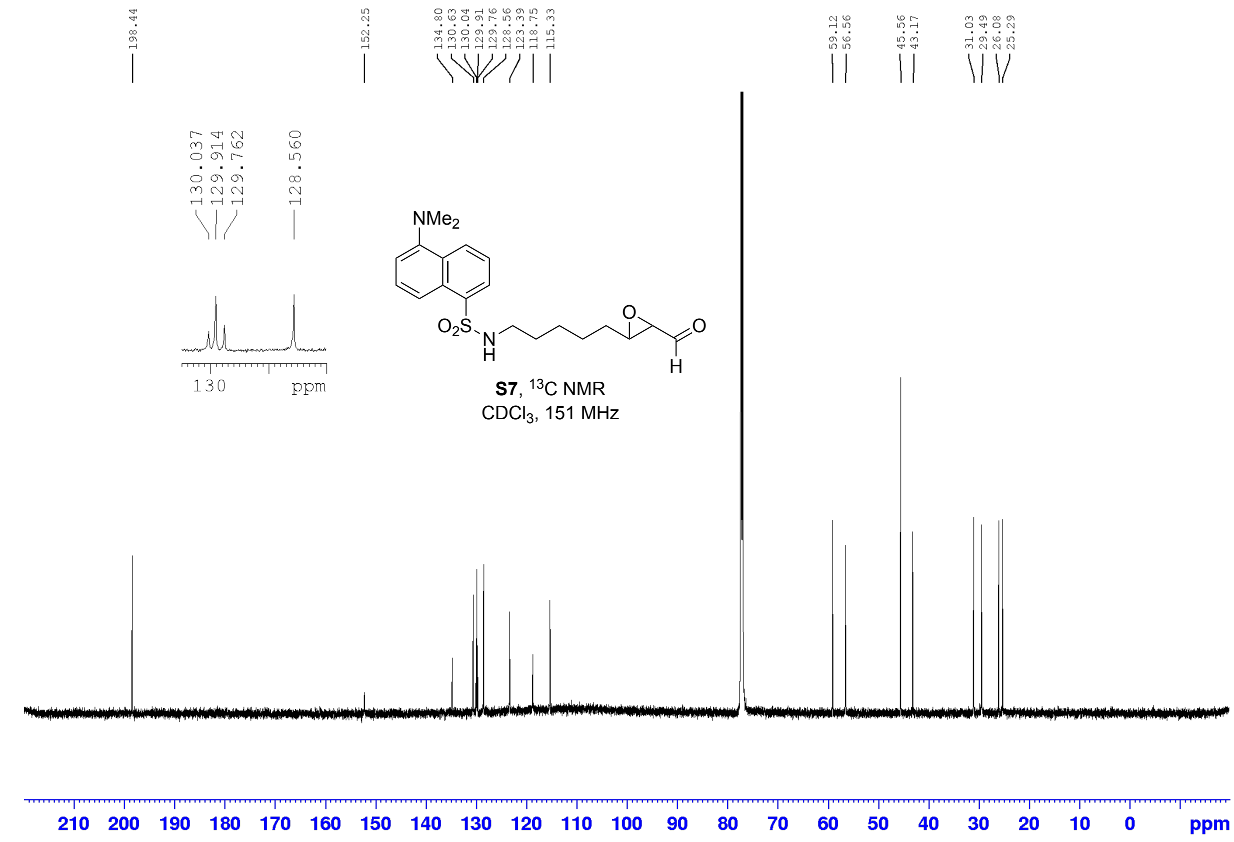
198.44 152.25 134.80 130.63 130.04 129.91 129.76 128.56 123.39 118.75 115.33 59.12 56.56 45.56 43.17 31.03 29.49 26.08 25.29
130.037 129.914 129.762 128.560
130 ppm
NMe2
O2S
N
H
O
O
H
S7, 13C NMR
CDCl3, 151 MHz
210 200 190 180 170 160 150 140 130 120 110 100 90 80 70 60 50 40 30 20 10 0 ppm

References

1. B. Wrackmeyer, *Prog. Nucl. Magn. Reson. Spectrosc.* 1979, **12**, 227-259.
2. R. K. Harris, E. D. Becker, S. M. Cabral De Menezes, P. Granger, R. E. Hoffman and K. W. Zilm, *Pure Appl. Chem*. 2008, **80**, 59-84.
3. S. Hitosugi, D. Tanimoto, W. Nakanishi and H. Isobe, *Chem. Lett.* 2012, **41**, 972-973.
4. A. R. Katritzky, Q.-Y. Chen and S. R. Tala, *Org. Biomol. Chem.* 2008, **6**, 2400-2404.
5. G. Gomori, in *Handbook of Biochemistry and Molecular Biology*, Fourth Edition, CRC Press: 2010; p 721.
6. R. D. Hanna, Y. Naro, A. Deiters and P. E. Floreancig, *J. Am. Chem. Soc*. **2016**, *138*, 13353-13360.
